# How loop lengths shape topological preferences of two-tetrad G-quadruplexes

**DOI:** 10.1093/nar/gkag590

**Published:** 2026-06-16

**Authors:** Amadeusz Woś, Karolina Zielińska, Karol Pasternak, Aleksandra Pawłowicz-Perczak, Zofia Gdaniec, Dorota Gudanis-Sobocińska, Witold Andrałojć

**Affiliations:** Institute of Bioorganic Chemistry, Polish Academy of Sciences, 61-704 Poznan, Noskowskiego 12/14, Poland; Institute of Bioorganic Chemistry, Polish Academy of Sciences, 61-704 Poznan, Noskowskiego 12/14, Poland; Institute of Bioorganic Chemistry, Polish Academy of Sciences, 61-704 Poznan, Noskowskiego 12/14, Poland; Institute of Bioorganic Chemistry, Polish Academy of Sciences, 61-704 Poznan, Noskowskiego 12/14, Poland; Institute of Bioorganic Chemistry, Polish Academy of Sciences, 61-704 Poznan, Noskowskiego 12/14, Poland; Institute of Bioorganic Chemistry, Polish Academy of Sciences, 61-704 Poznan, Noskowskiego 12/14, Poland; Institute of Bioorganic Chemistry, Polish Academy of Sciences, 61-704 Poznan, Noskowskiego 12/14, Poland

## Abstract

Smallest G-quadruplex (G4) structures comprise only two stacking G-tetrads. DNA sequences capable of forming such folds are extremely abundant in the human genome and a growing body of evidence shows that even these simplest G4 motifs play important roles in both gene regulation and in numerous pathological processes. However, our understanding of the sequence–structure relationship governing two-tetrad G4 formation remains limited. In the current work, we provide a systematic analysis of how loop length shapes the structural preferences of two-tetrad G4s by experimentally characterizing G4s formed by all 64 DNA sequences of the type 5′-GGT_*x*=1–4_GGT_*y*=1–4_GGT_*z*=1–4_GG-3′. Using a combination of biophysical methods, we were able to distinguish two-tetrad G4s from higher-order G4 assemblies and define several features in experimental data that are characteristic of this kind of structures. Through 2D NMR (nuclear magnetic resonance) analysis, we assigned specific loop topologies for 17 two-tetrad folds, identifying the *+l+l+l* (antiparallel-chair) topology as the most prevalent, followed by *−ld+l* (antiparallel-basket), with fewer examples of *d+pd* (antiparallel-basket) and −*l−l−l* (antiparallel-chair) G4s. Taken together, the gathered dataset delineates regions of sequence space occupied by G4 folds of different molecularities and loop topologies, revealing the length of the central loop as the main determinant of two-tetrad G4s’ structural preferences.

## Graphical Abstract

**Graphical Abstract:**
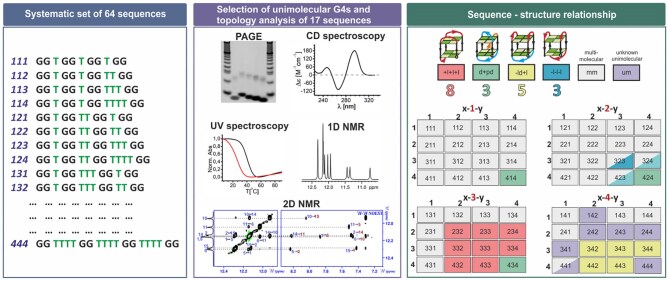

## Introduction

**Figure 1.**
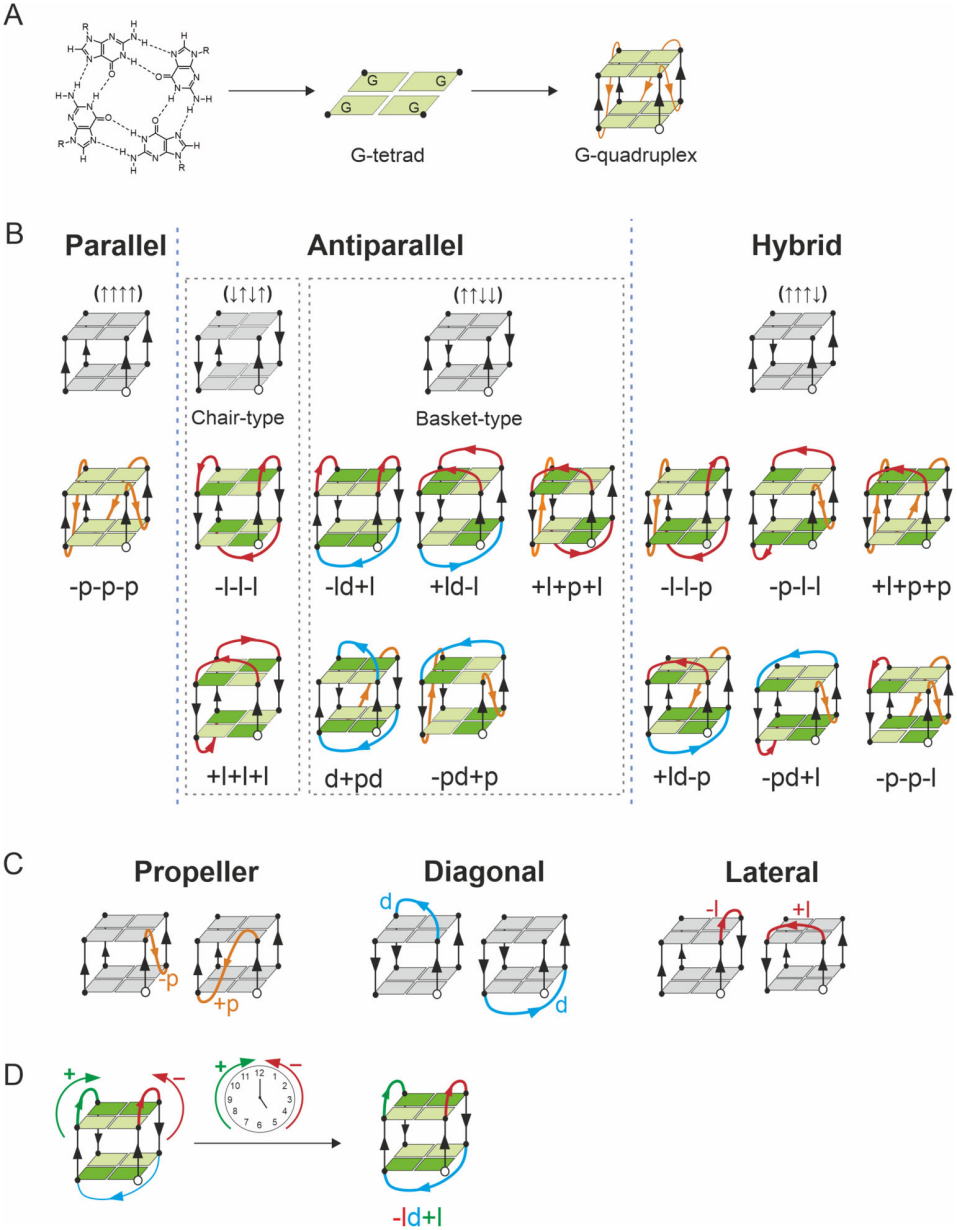
The topology of a G4 fold. (**A**) Chemical structure of a G-tetrad and illustration how tetrads stack to form a G4 core. (**B**) Fourteen loop topologies for canonical unimolecular G4s, grouped into “parallel,” “antiparallel,” and “hybrid” families. (**C**) The three loop types in canonical G4s; “+” or “−” descriptors are added to indicate a clockwise or anticlockwise loop directionality, respectively, for propeller and lateral loops, as defined in panel (D). (**D**) Scheme showing how topology symbols in panel (B) are derived from loop arrangements.

Nucleic acids rich in guanosine residues can form non-canonical structural motifs known as G-quadruplexes (G4s), whose main building blocks are planar arrangements of four guanosines held together by Hoogsteen-type hydrogen bonds, known as G-tetrads (Fig. 1A) [1]. Stacking of G-tetrads into a G4 core (Fig. 1A) requires coordination of metal ions, most commonly K^+^ or Na^+^, to screen the repulsive interactions between carbonyl atoms of guanine bases, making G4 folding metal ion dependent [2]. In the last decades these structures have become arguably the most studied family of non-canonical nucleic acid motifs, due to both the mounting evidence of their widespread biological importance and remarkable structural diversity associated with this seemingly simple structural motif. G4 structures were shown to be involved in translation regulation, replication, and telomere maintenance [3]. Importantly, G4 formation is also related to a series of pathological processes, as numerous G-rich sequences were identified in promoter regions of oncogenes, as well as in bacterial and viral genomes [4–6]. This fact, coupled with the intrinsic G4 structural heterogeneity, which increases the potential for selective targeting, makes these motifs particularly attractive targets for therapeutic interventions, and the search for G4-binding small molecules has become a vibrant field of research [4]. In parallel, G4s also attract much attention as building blocks of nanotechnological devices including sensors, molecular wires, and nanomachines [7]. However, both successful engineering of such G4-nanodevices and rational design of G4-binding ligands require a detailed understanding of three-dimensional structures assumed by these molecules and, in particular, how these structures are shaped by both the nucleic acid sequence and environmental factors. However, despite the determination of over 500 high-resolution G4 structures and numerous lower-resolution studies, the ability to predict G4 three-dimensional folds from DNA or RNA sequence remains limited owing to the sheer complexity of G4 conformational space.

Even for canonical G4-forming DNA sequences of the type 5′-G_*n*_N_*x*_G_*n*_N_*y*_G_*n*_N_*z*_G_*n*_-3′, composed of four guanine runs (*n* ≥ 2) separated by three stretches of arbitrary sequence, as many as 26 loop topologies of unimolecular G4 folds are theoretically possible [8]. Molecular dynamics studies have confirmed 14 of these arrangements to be sterically feasible [9] (listed in Fig. 1B). In this formalism the topology of a given G4 is described by the conformations of loop regions connecting the guanosine runs, each of which can assume a propeller, lateral, or diagonal arrangement (Fig. 1C). Figure 1D exemplifies how the information about loop progression unequivocally defines a G4’s topology. Alternatively, G4 folds can be classified into three families based on the relative orientations of the four strands forming the G4 core—parallel, antiparallel, and hybrid (Fig. 1B). These families are also often referred to as “G4 topologies” in the literature. However, as seen in Fig. 1B, several topologically distinct types of G4 structures can share the same strand orientations, making the loop-based description much more complete.

The conformational landscape of G4-forming molecules is further complicated by the fact that they can fold into bi-, tri-, or tetramolecular G4s, not just unimolecular ones. Regardless of their molecularity, G4 folds can also contain additional “non-canonical” elements such as bulges [10], V-loops [11], or tetrads composed of non-guanosine bases [12]. Additional structural elements can also form within G4 loops and flanking regions, including canonical and non-canonical base-pairs, base-triples, or entire DNA/RNA hairpins [13]. On top of this structural diversity, the energetics of G4 formation are also highly dependent on environmental factors [14], most notably the identity of metal cations present, but also strand concentration, presence of molecular crowding agents, or in some cases sample pH [15]. All these factors make successful prediction of G4 structures from oligonucleotide sequences a monumental task.

The influence of loop length and sequence on G4 topology and stability has been thoroughly investigated previously [16–20]. While not providing a definitive answer applicable to an arbitrary DNA molecule, these studies have allowed to elucidate several general rules. Shorter loops generally lead to more stable G4 structures and tend to favor parallel topologies often promoting intermolecular assemblies. Similarly, it was shown that among the three loops present, a longer central one is especially proficient at inducing stable non-parallel folds [20].

To date, the vast majority of G4 research has focused on systems with at least three G-tetrads, reflecting the perceived low stability of two-tetrad G4s. Some more conservative definitions even require a G4 core to contain at least three tetrads [20–22]. However, multiple stable two-tetrad G4s were already identified and characterized in both biological and biotechnological contexts. For example, the formation of two-tetrad G4s was unambiguously confirmed for oligonucleotides derived from both human [23, 24] and *Caenorhabditis elegans* [25] telomeric DNA and very early on for the thrombin-binding aptamer [26]. The question to what degree putative two-tetrad G4s can fold in biologically relevant conditions, and what their topological preferences are, becomes even more pressing when one considers that simple statistical arguments predict such sequences to be more abundant in the genomes than those forming G4s with a higher number of tetrads. This expectation was confirmed for the human genome by bioinformatic analyses [27, 28], which revealed a marked overrepresentation of such sequences in, for example, gene promoter regions [27], suggesting possible roles of two-tetrad G4s in transcription regulation. Further studies confirmed a prevalence of similar sequence motifs in the genomes of other organisms, including bacterial and viral pathogens [29, 30].

Systematic studies of two-tetrad G4s’ conformational preferences remain scarce, with the most comprehensive works examining only about a dozen sequences each [27, 31, 32]. The first paper, which focused specifically on loop-length effects in two-tetrad G4s, investigated only systems in which all three loops were of the same length and composed entirely of thymidine residues [27]. It concluded that G4 formation can be observed for loop lengths up to five nucleotides, which is significantly less than in three-tetrad G4s [18]. More recent work investigated the propensity of two-tetrad G4s to adopt a unimolecular, parallel fold and thus concentrated on sequences containing very short loops [31, 32]. It concluded that such folds are intrinsically unstable for both DNA [31] and RNA [32] molecules unless accompanied by additional flanking elements such as a base triples [33] or stacking interactions with another G4 unit [34]. These results also demonstrate that conclusions derived from the rich body of work on three-tetrad G4s cannot be directly applied to their two-tetrad counterparts. Similarly, research exploring the loop-length requirements for the preferential formation of a specific antiparallel fold (*−ld+l*, illustrated in Fig. 1B) has found them to be different for two- and three-tetrad G4 cores [35]. Taken together, these findings highlight the need for a comprehensive, systematic study focused specifically on two-tetrad G4 topological preferences and formation propensity.

In this paper, we investigate G4 structures formed by the complete set of DNA molecules of the type 5′-GGT_*x*=1–4_GGT_*y*=1–4_GGT_*z*=1–4_GG-3′ in the presence of K^+^ ions. Through a combination of biophysical techniques widely used for G4 characterization [36], i. e. UV, CD, and 1D ^1^H NMR (nuclear magnetic resonance) spectroscopies, as well as native gel electrophoresis, we assessed the number of G4 folds formed by each molecule, along with their thermal stability and molecularity. Importantly, for many sequences that predominantly folded into a single, unimolecular G4 structure, we were able to assign specific G4-topologies (as classified in Fig. 1B) through 2D NMR analysis. This analysis uncovered that the majority of unimolecular G4s belonged to just two topologies: *+l+l+l* and *−ld+l*, with fewer examples of *d+pd* and −*l−l−l*. Moreover, the systematic nature of the studied set of molecules allowed us to map regions of the sequence space that preferentially fold into G4 structures of specific topologies and molecularities, including “transition zones” in which multiple folds and molecularities coexist. The resulting dataset provides a rich source of information about the sequence–structure relationship for two-tetrad G4s, useful for both future basic studies of G4 conformational landscapes and for the practical design of two-tetrad G4s of specific structural preferences.

## Materials and methods

### DNA sample preparation

All DNA oligonucleotides were purchased from Metabion GmbH. The acquired material was purified from residual organic cation, triethylamine, remaining after high performance liquid chromatography by precipitation from 2% NaClO_4_/acetone. DNA concentrations were determined from absorbance at 260 nm using the corresponding extinction coefficients. Unless stated otherwise, oligonucleotides were dissolved and annealed in 10 mM potassium phosphate buffer (pH 7.6) containing 150 mM KCl and 0.1 mM ethylenediaminetetraacetic acid. After annealing, samples were incubated at 5°C for at least 2 weeks prior to measurements at DNA concentrations appropriate for each experimental technique (see below).

### Ultraviolet melting measurements

Ultraviolet (UV) melting curves were recorded using a JASCO V-750 spectrophotometer (Jasco Deutschland GmbH, Pfungstadt, Germany) equipped with a water-cooled thermoprogrammer and 5 mm (150 μl) quartz cells. Absorbance was monitored at 295 nm during a cycle consisting of heating from 5°C to 90°C at a rate of 0.2°C min^−1^, followed by cooling back to 5°C at the same rate. Samples were protected against evaporation with silicone oil. The DNA concentration in the melting analysis was ~1.7 × 10^−5^ M unless otherwise noted. Prior to the experiments, samples were equilibrated at 5°C for at least 2 weeks. T_m_ values were obtained from the first derivative of UV melting curves, recorded as molar absorbance (M^−1^ cm^−1^) at 295 nm as a function of temperature, using MeltWin 3.5.

### Circular dichroism spectroscopy

Circular dichroism (CD) spectra were recorded on a Jasco J‐815 spectropolarimeter (Jasco Deutschland GmbH, Pfungstadt, Germany) equipped with a Peltier temperature controller. Cuvettes with a path length of 0.5 cm were used (sample volumes 1300 μl). DNA oligonucleotides were dissolved in the same buffer as used for NMR studies, at a sample concentration of ~1.4 × 10^−5^ M. All experiments were conducted at least 2 weeks after sample preparation. Measurements were collected at 5°C in the range between 225 and 340 nm wavelength as a sum of three repetitions, and the buffer baseline was subtracted from each spectrum. CD spectra were expressed in the units of molar ellipticity Δε [M^−1^ cm^−1^].

The collected CD dataset was analyzed through principal component analysis (PCA) and hierarchical clustering. PCA was performed using in-house Python scripts through singular value decomposition of the 64 × 126 CD data matrix (constructed from 64 samples, with spectra containing 126 datapoints; 215–340 nm range). First three principal components were found to explain 98.7% of the variance in the data. Hierarchical clustering was performed as implemented in the SciPy-cluster Python package, with Ward’s method selected as the cluster definition criterion.

### Native polyacrylamide gel electrophoresis

DNA oligonucleotides were dissolved in 10 mM potassium phosphate buffer containing 150 mM KCl to a final concentration of 8 × 10^−6^ M and incubated at 5°C for at least 2 weeks prior to electrophoresis. Before loading, 2 μl of 50% glycerol was added to each sample. Thermo Scientific O’RangeRuler 5-bp DNA ladder was used as reference. Native polyacrylamide gel electrophoresis (PAGE) was performed on 20% polyacrylamide gels (acrylamide:bisacrylamide, 29:1) 0.25× TBE buffer supplemented with 25 mM KCl. Electrophoresis was carried out in an ice bath at 100 V for 4 h using an XCell SureLock Mini-Cell system (Thermo Fisher Scientific). Gels were stained with SYBR Gold (17.5 μl in 150 ml of 0.25× TBE) for 10 min and destained for 2 min in 0.25× TBE. Fluorescence images were acquired using a Typhoon phosphorimager (Cytiva) and analyzed with MultiGauge software (version 3.0, Fujifilm).

### NMR spectroscopy

NMR spectra were acquired mostly on a Bruker AVANCE III 700 MHz spectrometer equipped with a QCI-P CryoProbe. Only the ^31^P-observed ^1^H-^31^P-COSY spectra were recorded on a 500 MHz Bruker Avance III spectrometer with a room-temperature BBO probe instead. The spectra were acquired using Bruker Topspin 3.6.5 software and processed with Bruker TopSpin 4.1.3 software. For the primary set of 1D ^1^H NMR spectra, DNA concentrations of 4 × 10^−4^ M were used and the experiments were conducted after at least 2 weeks sample incubation at 5°C. For 2D NMR analysis of selected systems, new samples with concentrations >1 × 10^−3^ M were prepared. These measurements were generally conducted in the standard buffer conditions at 20°C unless stated otherwise. Resonance assignment of non-exchangeable protons and phosphorus atoms was achieved using standard procedures [37, 38] through the analysis of NOESY and ^1^H-^31^P-COSY spectra, while the imino proton resonances were assigned using ^1^H-^13^C HMBC spectra [39]. G4 topology assignment was achieved using long-range NOE connectivities involving the imino and aromatic protons of guanosine residues, as described in more detail in the Supplementary data.

## Results

### Design of the study

To systematically investigate how loop length influences the conformational preferences of two-tetrad DNA G4s, we experimentally characterized the entire set of 64 DNA molecules of the type 5′-GGT_*x*=1–4_GGT_*y*=1–4_GGT_*z*=1–4_GG-3′ in presence of K^+^ ions. For brevity, each sequence will be referred to by the number of thymidine residues in its loops as an identifier. Thus, for example, the DNA sequence 5′-GG**TT**GG**TTT**GG**T**GG-3′ will be referenced as **231**. In some figures, a numbering scheme (1–64) corresponding to column 1 of Table 1 is used instead (e.g. **111** = 1, **143** = 15).

**Table 1.** Summary of the main experimental observations for the 64 studied systems, including G4 melting temperature, type of CD profile, number and mobility of gel bands, and molecularity derived from NMR spectra

Nos 1–64	ID	T_m_ (°C)	Type of CD profile^a^	Number of bands in PAGE^b^				Number of species in NMR^c^				Loop topology^f^
				s	m	f	vf	Unimolecular		Multimolecular		
								Major	Minor	Major	Minor	
1	111	61.6*	Parallel	1	-	-	-	-	-	a	-	-
2	112	46.5*	Parallel	2	-^e^	-^e^	-	-	-	a	-	-
3	113	43.1*	Mostly parallel	1	1	1	1	-	-	m/a	-	-
4	114	40.6*	Mixture	-	1	1	-	-	-	m	-	-
5	121	46.0*	Parallel	1	1	-^e^	-	-	-	a	-	-
6	122	38.6*	Parallel	2	-^e^	-^e^	-	-	-	m/a	-	-
7	123	40.0*	Parallel	-	1	1	-	-	-	1	-	-
8	124	33.1*	Mixture	-	1	1	-	-	-	m	-	-
9	131	42.0*	Mixture	-^e^	1	1	1	-	-	1	a	-
10	132	34.6*	Mixture	-^e^	-	1	-	-	-	m	-	-
11	133	40.4*	Mostly parallel	-	1	1	-	-	-	m	-	-
12	134	29.9*	Mostly antiparallel	-	1	1	-	-	-	m	-	-
13	141	41.4*	Mixture	-	-	1	-	-	1	m	-	-
14	142	33.9*	Antiparallel 1	-	-	1	-	1	-	-	a	-
15	143	23.6	Mostly antiparallel	-	-	-	1	-	1	2	-	-
16	144	16.7	Mostly antiparallel	-	1	1	-	-	-	m	-	-
17	211	65.6*	Parallel	3	-	-	-	-	-	m/a	-	-
18	212	60.7*	Parallel	2	-	2	-	-	-	1	-	-
19	213	46.5*	Mixture	-	1	2	-	-	-	m	-	-
20	214	36.5*	Mixture	-	1	1	-	-	-	m	-	-
21	221	41.6*	Parallel	2	1	-	-	-	-	m	-	-
22	222	43.9*	Mixture	-	1	-	-	-	1	1/a	1	-
23	223	37.6*	Mostly antiparallel	-	1	1	-	-	1	m	m	-
24	224	31.5*	Mostly antiparallel	-	2	1	-	-	1	m	m	-
25	231	31.0*	Mixture	-	-	1	-	-	-	m	-	-
26	232	46.4	Antiparallel 2	-	-	1	-	1	-	-	-	*+l+l+l*
27	233	45.8	Antiparallel 2	-	-	1	-	1	-	-	-	*+l+l+l*
28	234	38.9	Antiparallel 2	-	-	1	-	1	-	-	-	*+l+l+l*
29	241	31.5*	Mixture	-	-	2	-	-	1	m	-	-
30	242	39.7	Antiparallel 1	-	-	1	-	1	2	-	-	-
31	243	43.6	Antiparallel 1	-	-	-	1	2	1	-	-	-
32	244	36.9	Antiparallel 1	-	-	1	-	1	2	-	-	-
33	311	62.5*	Mostly parallel	1	1	-	-	-	-	m/a	-	-
34	312	58.2*	Mixture	-	1	-	-	-	-	1	-	-
35	313	44.9*	Mostly antiparallel	-	1	-	-	-	-	m	-	-
36	314	42.1*	Mixture	-	2	-	-	-	-	m	-	-
37	321	42.6*	Mostly parallel	-	1	-	-	-	-	1	-	-
38	322	39.7*	Mixture	-	2	-	-	-	-	1	-	-
39	323	39.0	Antiparallel 2	-	1	-	-	1	1	1	-	*−l−l−l*
40	324	36.6	Antiparallel 2		1	-	-	1	1	1/2^f^		*−l−l−l* (?)
41	331	29.4*	Mostly antiparallel	-	1	-	-	-	1	m	-	-
42	332	45.8	Antiparallel 2	-	-	1	-	1	-	-	-	*+l+l+l*
43	333	48.8	Antiparallel 2	-	-	1	-	1	-	-	-	*+l+l+l*
44	334	43.2	Antiparallel 2	-	-	1	-	1	-	-	-	*+l+l+l*
45	341	24.1	Antiparallel 1	-	-	1	-	1	1	-	a	-
46	342	47.8	Antiparallel 1				1	1	-	-	-	*−ld+l*
47	343	50.0	Antiparallel 1	-	-	-	1	1	1	-	-	*−ld+l*
48	344	44.1	Antiparallel 1	-	-	-	1	1	1	-	-	*−ld+l*
49	411	61.7*	Mostly parallel	-	1	1	-	-	-	m/a	-	-
50	412	56.1*	Mixture	-	1	1	-	-	-	1	-	-
51	413	43.2*	Mixture	-	2	1	-	-	-	1	1	-
52	414	33.7	Antiparallel 1	-	-	-	1	1	-	-	1	*d+pd*
53	421	41.1*	Mostly parallel	-	2	-	-	-	-	1/2^f^	1	-
54	422	38.3*	Mostly parallel	-	2	-	-	-	-	2	1	-
55	423	33.1	Antiparallel 2	-	2	-	-	1	1	1	-	*−l−l−l* (?)
56	424	41.6	Antiparallel 1	-	-	-	1	1	-	-	-	*d+pd*
57	431	40.5*	Mixture	-	1	-	-	-	-	m	-	-
58	432	37.3	Antiparallel 2	-	-	1	-	1	1	-	-	*+l+l+l*
59	433	41.4	Antiparallel 2	-	-	1	-	1	1	-	-	*+l+l+l*
60	434	40.7	Antiparallel 1	-	-	-	1	1	-	-	-	*d+pd*
61	441	22.5*	Antiparallel 2	-	1	-	-	m	-	m	-	-
62	442	43.1	Antiparallel 1	-	-	-	1	1	-	-	-	*−ld+l*
63	443	45.0	Antiparallel 1	-	-	-	1	1	1	-	-	*−ld+l*
64	444	40.5	Antiparallel 1	-	-	-	1	2	-	-	-	-

* UV melting curves with hysteresis

^a^According to classification outlined in Fig. 3

^b^Bands migration in PAGE: s—slow; m—medium; f—fast; and vf—very fast; see Fig. 5

^c^At 150 mM KCl and 0.4 mM DNA concentrations; uni- and multimolecular G4s are counted separately (according to arguments outlined in the article text); m—multiple G4 forms (yielding sharp imino resonances, but uncountable to overlap), a—G4 aggregates (yielding broad imino proton envelopes)

^d^Loop topology of the dominant unimolecular form (if present), according to the classification provided in Fig. 1.

^e^Multiple weak bands

^f^Imino protons of multimolecular form(s) likely overlap with amino and/or thymidine imino protons making counting the forms uncertain

The goal of this investigation is to assess for each molecule (i) its capability to assume a G4 fold and its thermal stability; (ii) the number of G4 species formed; and (iii) the molecularity and, whenever possible, topology of each species (according to Webba da Silva classification; Fig. 1B). Such a dataset would provide an invaluable resource for understanding the sequence–structure relationship for two-tetrad G4s. To collect all these diverse types of information, we employed a combination of four complementary biophysical techniques: UV, CD, and NMR spectroscopies, as well as native gel electrophoresis. In principle, this set of methods can provide all the necessary data outlined above and the roles of each technique in G4 structural characterization were recently reviewed [36].

As G4 folding depends on multiple factors besides the oligonucleotide sequence—especially the type and concentration of metal cations—great care was taken in selecting proper experimental conditions. A phosphate buffer of pH 7.6 containing 150 mM K^+^ was selected for the main set of experiments, both because these conditions are close to the in-cell salt and pH values and because relatively high potassium concentration was assumed to be necessary to fold many of the inherently less stable two-tetrad G4s. For the same reason, 5°C was used as the default experimental temperature to maximize the number of cases in which two-tetrad G4 structures form quantitatively. Because G4 folding can be slow and the formation of metastable states lingering for many days was often reported, all samples were incubated at 5°C for 2 weeks prior to measurements in the hope of approaching conformational partitioning close to thermodynamic equilibrium. The basic set of experimental data collected for each studied sequence is gathered in Supplementary Fig. S1 (panels 1–64).

The discussion of the obtained results is organized as follows. First, we provide a general overview of the data collected with each method across all studied molecules (summarized in Table 1). Next, we focus on individual DNA sequences to determine molecularity and, for intramolecular G4s, assign topology wherever a dominant, well-resolved form is present. Finally, we return to the complete dataset to map regions of sequence space associated with different G4 molecularities and topologies and to define their boundaries.

### UV melting analysis

**Figure 2.**
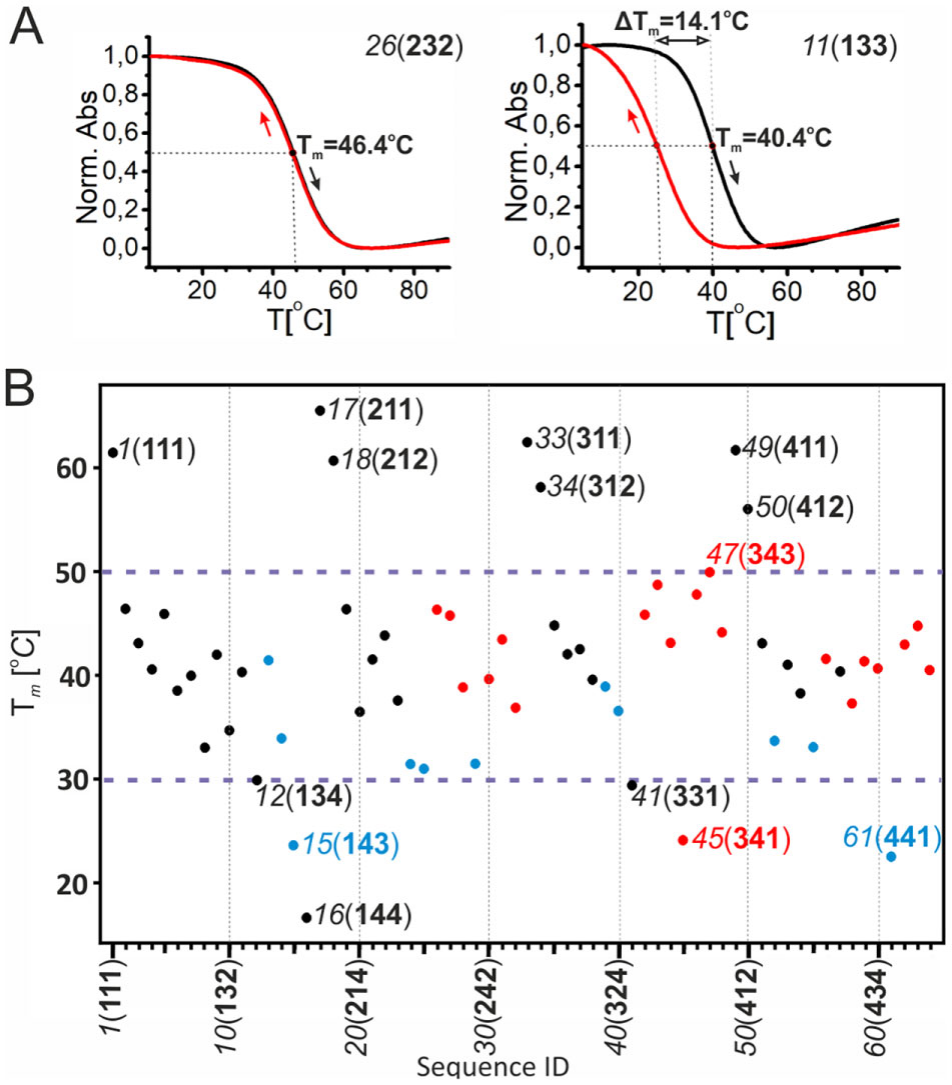
UV melting experiments. (**A**) Examples of melting curves observed at *λ*=295 nm with (right panel) and without (left panel) hysteresis. Each cycle consisted of heating from 5°C to 90°C (black curve), followed by cooling back to 5°C (red curve). (**B**) The measured set of T_m_ values. The points are color coded according to the molecularity of the dominant G4 form(s) observed in NMR (at higher DNA concentrations). Red dots denote systems that fold uniquely into unimolecular G4s, blue dots those featuring a significant fraction of unimolecular G4s (likely further amplified at UV melting concentrations), and black dots systems for which multimolecular G4s dominate.

UV melting curvesf measured at 295 nm showed the characteristic inverted profiles expected for G4s for all 64 studied DNAs (example profiles in Fig. 2A and all melting curves in Supplementary Fig. S2), suggesting that each sequence forms a G4 fold, a conclusion later confirmed by NMR spectroscopy (see below). However, the thermal stabilities varied significantly within the set (Table 1 and Fig. 2B): most melting temperatures (51/64) were between 30°C and 50°C, with outliers ranging from 16.7°C to 65.6°C (for sequences **144** and **211**, respectively). To interpret these data correctly, it is crucial to consider the molecularity of the dominant G4 structure in each sample—information that will be obtained later in this study from NMR and electrophoretic analyses. When considering only the 31 systems that fold either uniquely or predominantly into unimolecular two-tetrad G4s in UV melting conditions (red and blue dots in Fig. 2B, respectively), the range of observed T_m_ values reduces to 22.5°C–50.0°C (with an average at 38.8°C). This makes the studied set of two-tetrad G4s generally less stable in the presence of K^+^ ions than their three-tetrad counterparts (with T_m_ values usually >50°C under similar conditions) [17, 20], as expected. On the other hand, the observed thermal stabilities are in general still sufficient for most two-tetrad G4s to remain at least partially folded at physiologically relevant temperatures.

Thirty-eight of the studied samples showed hysteresis in their melting profiles (up to >20°C), as indicated by a star in Table 1. Subsequent structural studies revealed that this phenomenon occurred only in systems containing multimolecular G4s within the ensemble of sampled structures.

### CD spectroscopy

For G4 structures composed of three or more tetrads, CD spectroscopy is often used to differentiate parallel, antiparallel, and hybrid topologies (Fig. 1B, top), because each produces a characteristic spectral shape. However, the profile of the CD spectrum of a G4 is not directly dictated by its strand directionality, but by the geometry of G-tetrad stacking within its core [40, 41]. Two stacking arrangements between a pair of tetrads are possible: (i) homopolar (also known as head-to-tail) stacking, producing a CD profile often associated with parallel G4s; and (ii) heteropolar (or, alternatively, head-to-head/tail-to-tail) stacking, which gives rise to a CD spectrum characteristic of antiparallel G4s (Supplementary Fig. S3A). This correspondence occurs because in parallel G4s all tetrads stack in the homopolar fashion, while in antiparallel ones exclusively heteropolar stacking is present (Supplementary Fig. S3B). The distinct CD profile often associated with hybrid topologies is a combination of these two basic spectral shapes, as hybrid G4s with three or more tetrads contain at least one occurrence of each stacking type (Supplementary Fig. S3B).

However, in two-tetrad G4s only a single stacking interaction between G-tetrads is present. This interaction assumes a homopolar geometry for parallel G4s and a heteropolar one for both antiparallel and hybrid topologies (Supplementary Fig. S3C). Consequently, antiparallel and hybrid two-tetrad G4s produce similar CD spectra, thus CD can only distinguish the parallel topology. A CD profile typically associated with hybrid folds may appear only if (i) mixture of parallel and antiparallel/hybrid folds coexists in the sample, or (ii) higher-order structures containing more than two G-tetrads are present.

**Figure 3.**
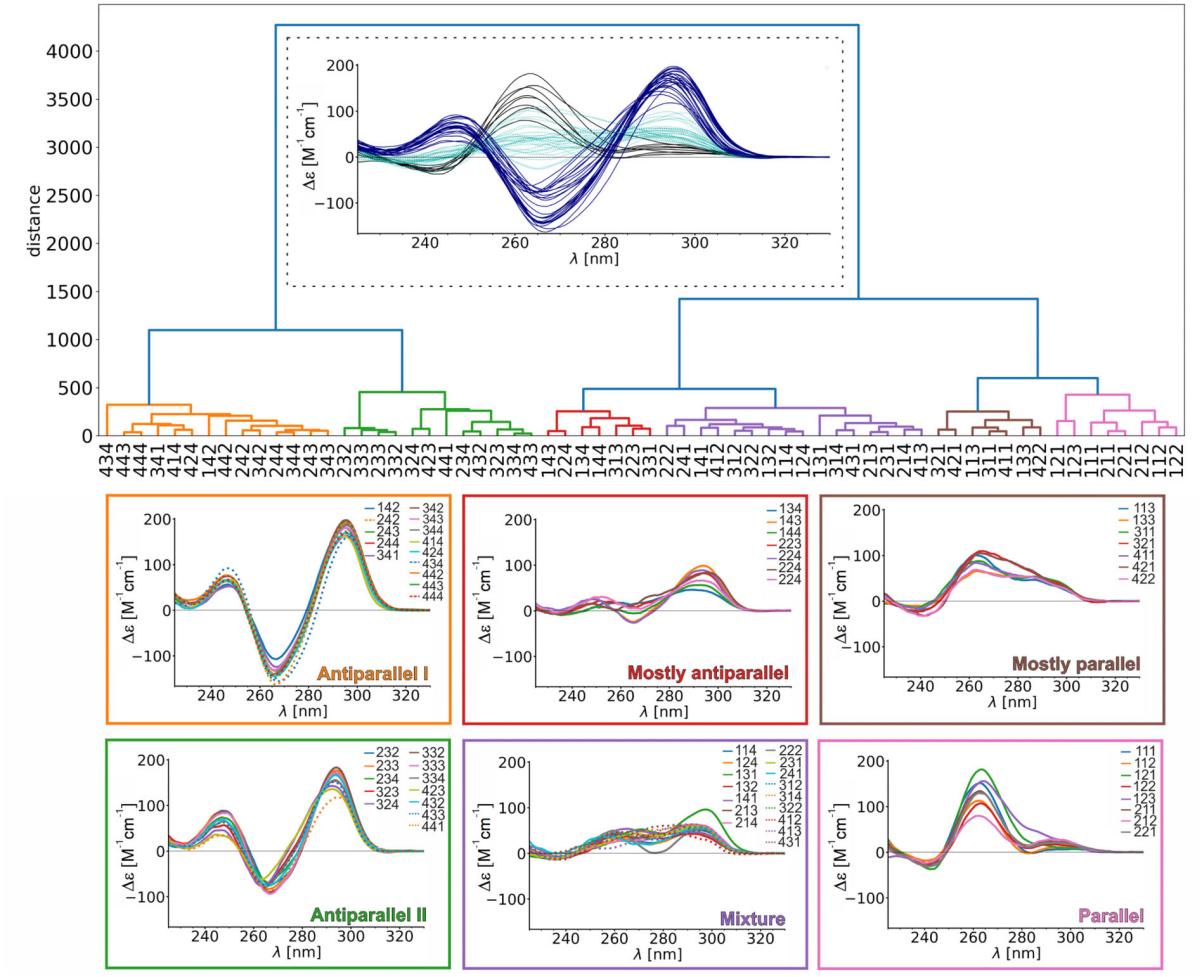
CD spectra. The complete set of spectra is shown in the inset at the top, colored according to a visual classification of the spectral profiles (see text). The rest of the figure depicts the classification of the spectra into six distinct subtypes achieved by hierarchical clustering analysis (see text).

Visual inspection of CD spectra measured for the 64 studied sequences shows that 26 samples produce profiles characteristic of heteropolar tetrad stacking (navy in Fig. 3, top inset; each spectrum shown separately in Supplementary Fig. S4), with a positive peak around 295 nm and a negative around 265 nm. In turn, eight samples give rise to a profile akin of the parallel one with a single maximum around 260 nm (black in Fig. 3, top inset). The remaining molecules exhibit various spectral shapes in between these two extremes (cyan in Fig. 3, top inset).

The collected CD dataset was further analyzed using hierarchical clustering and PCA. Based on the clustering results, the “cyan” subset of spectra from Fig. 3 was further divided into three categories: “predominantly antiparallel,” “mixed,” and “predominantly parallel” (Fig. 3). Interestingly, both hierarchical clustering (Fig. 3) and PCA (Supplementary Fig. S5) also revealed two subgroups among samples producing the antiparallel CD profile (navy in Fig. 3): the “antiparallel 1” subgroup with a more pronounced negative peak around 265 nm and “antiparallel 2” with a weaker band in that region. Subsequent NMR analysis showed that many samples in both groups contained only a single structural form and that the “antiparallel 1”/“antiparallel 2” distinction correlated with G4 topologies adopted by these molecules (Fig. 8). The resulting classification of CD data into six groups, depicted in Fig. 3 and summarized in Table 1, will be used throughout the rest of the discussion.

### 1D ^1^H NMR spectroscopy

**Figure 4.**
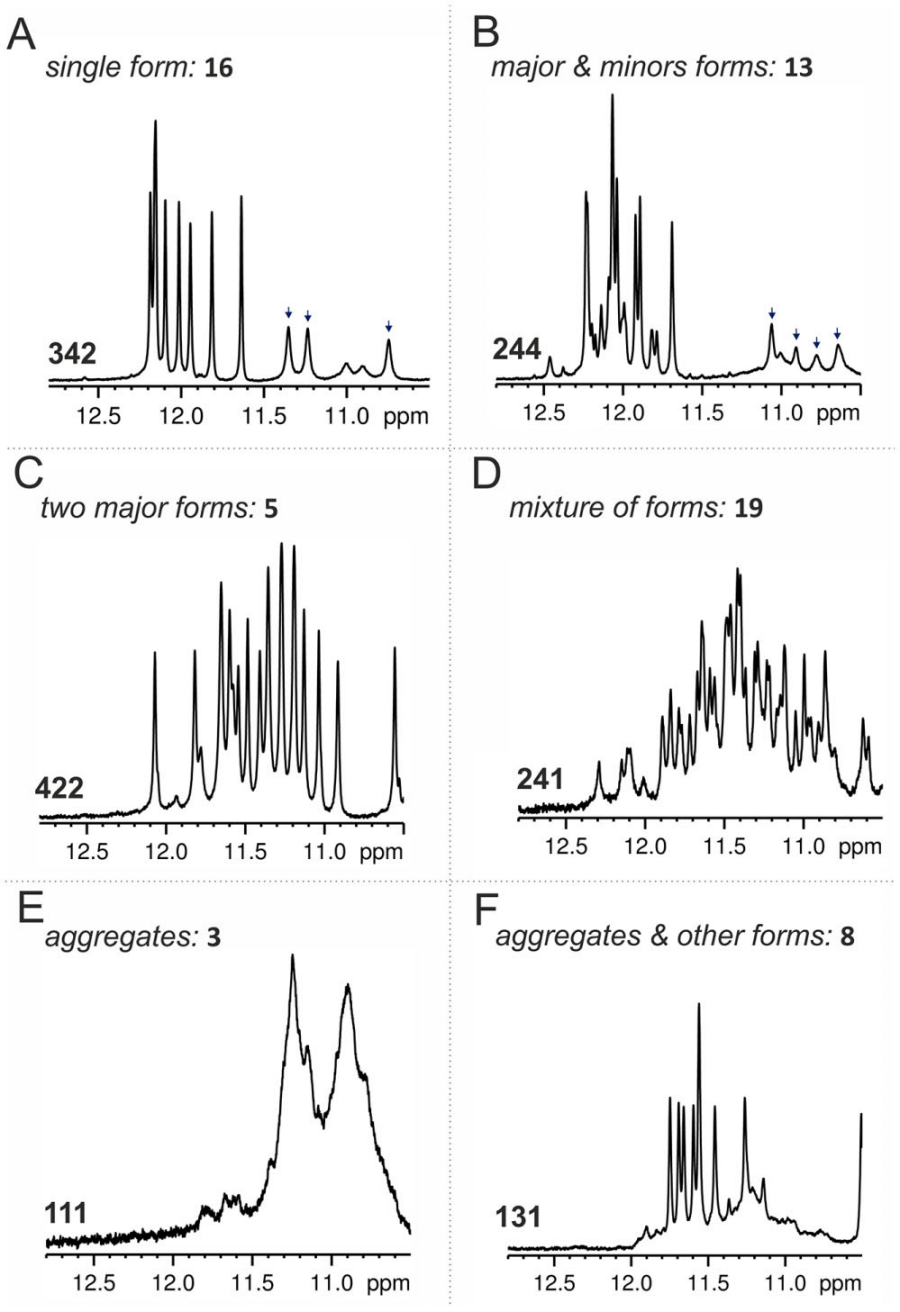
Representative 1D ^1^H NMR spectra (imino proton region) illustrating the diverse conformational equilibria. (**A**) a single dominant G4 fold; (**B**) a dominant G4 fold accompanied by a minor form; (**C**) two or three abundant G4 folds in equilibrium; (**D**) a complex mixture of multiple G4 folds; (**E**) G4 aggregates or misfolded/kinetically unstable species characterized by broad imino proton envelopes; and (**F**) a mixture of well-resolved G4 folds and other forms. Arrows indicate amino proton resonances. A full list of sequences assigned to each category is given in Supplementary Table S1.

All 1D ^1^H NMR spectra recorded at 5°C showed imino proton signals exclusively in the 10.5–12.5 ppm region, confirming the formation of G4 structures (all spectra shown in Supplementary Fig. S6). Each two-tetrad G4 should give rise to eight imino proton resonances in this region. A single set of such resonances was observed for 16 of the studied molecules, indicating that these sequences adopt a single G4 form (Fig. 4A and Supplementary Table S1). Furthermore, for 13 more systems a single dominant G4 form coexisted with one or more minor states (Fig. 4B). Five samples featured the coexistence of two or three G4 structures, present in comparable populations (Fig. 4C). As the number of imino proton resonances increased, overlap made accurate estimation of the number of coexisting G4 folds impossible and these samples were labeled as containing “multiple forms” (Fig. 4D). Finally, in some cases only a broad envelope of imino proton signals was observed without discernable peaks (Fig. 4E). This behavior can be ascribed either to the formation of G4 aggregates or to the presence of kinetically unstable or misfolded forms. In several samples, such broad imino proton envelopes were also observed to coexist with one or more sets of well resolved resonances (Fig. 4F).

Overall, the 1D ^1^H NMR data confirmed G4 formation for all studied oligonucleotides and provided an estimate of the number of different G4 folds coexisting in equilibrium.

### Native gel electrophoresis

**Figure 5.**
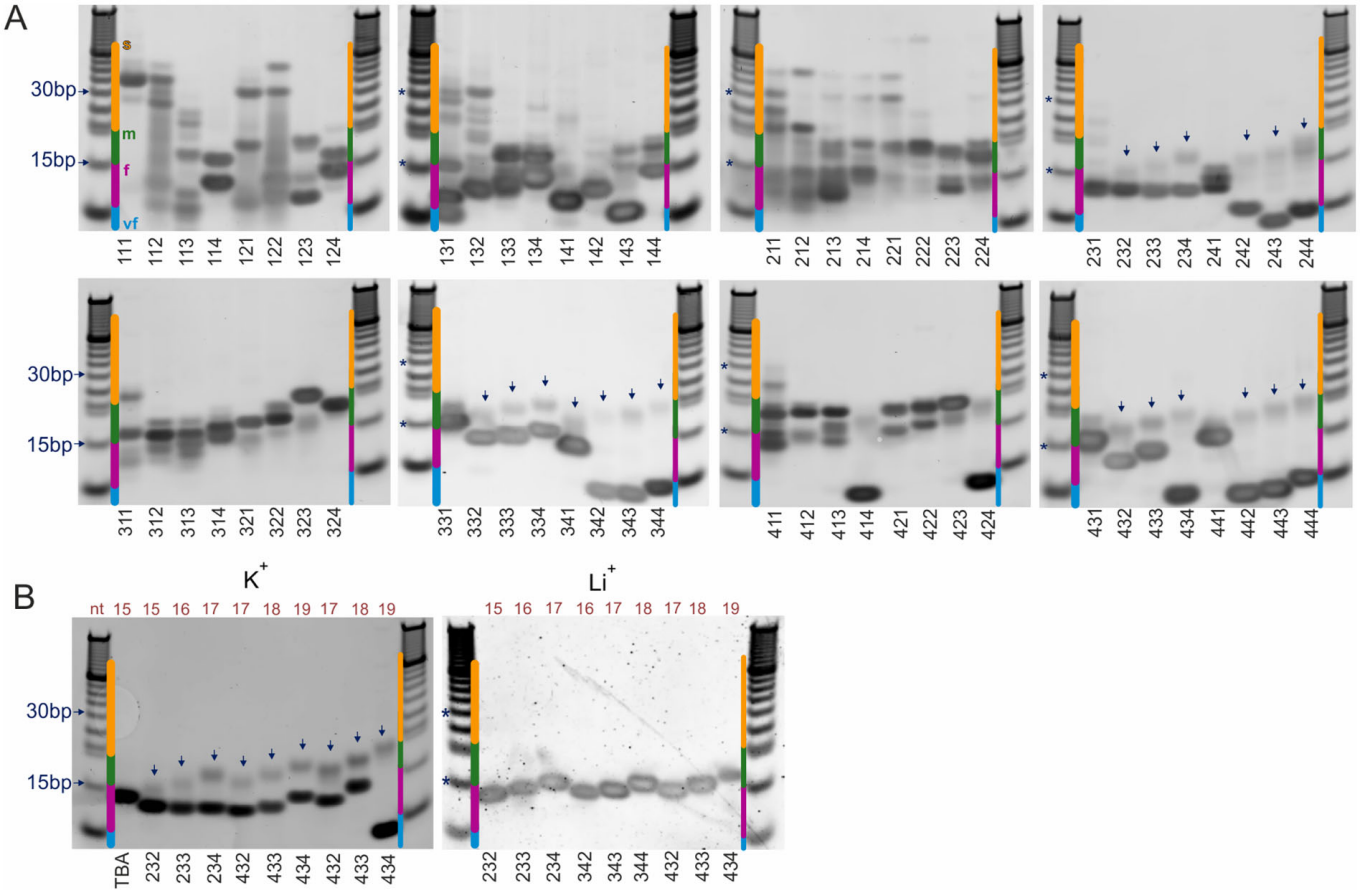
Native PAGE analysis of the full set of 64 DNA sequences. (**A**) DNA samples incubated in the presence of K^+^ ions. (**B**) Comparison of electrophoretic mobilities of selected sequences in the presence of K⁺ and Li⁺ ions. TBA denotes the thrombin-binding aptamer. Migration rates are classified into four color-coded categories: slow (orange, s), medium (green, m), fast (magenta, f), and very fast (blue, vf). Arrows indicate bands corresponding to unstructured single strands. The 15 bp and 30 bp markers of the reference ladder are explicitly named to guide the reader.

Electrophoreticf methods are often used to elucidate the molecularity of nucleic acid structures, including G4s. When the method was applied to the studied set of molecules, as shown in Fig. 5A, a remarkably wide range of mobilities was observed, with the fastest bands migrating ahead of the first marker of the reference ladder (10 bp) and the slowest ones displaying mobilities similar to the seventh marker (40 bp). Importantly, many samples gave rise to several distinct bands with markedly different migration rates, suggesting coexistence of G4s of different molecularities. Moreover, when considering the entire dataset almost a continuum of migration speeds was observed, making it impossible to draw clear boundaries between regions corresponding to G4 folds of specific molecularities. To facilitate the interpretation of the obtained data, we estimated the electrophoretic mobility of the unstructured, single-stranded forms of our oligomers, by performing the PAGE experiment using samples incubated in the presence of Li^+^ cations. In these conditions each oligomer produced a single band with a migration speed comparable with or slightly faster than the second marker of the ladder (15 bp), with a moderate amount of variability depending on sequence length (Fig. 5B, right). Interestingly, also in the presence of K^+^ ions many oligomers produce weak bands in this region that follow the same sequence length-dependent pattern (Fig. 5B, left), suggesting the presence of a small population of unstructured single strands. Their migration speed, slightly slower than in the presence of Li^+^ and falling between the second and third markers (dubbed “medium” in Fig. 5 and Table 1), will be used as a reference point when classifying other bands present in the gel.

When considering the bands migrating above the third marker of the ladder (marked “slow” in Fig. 5 and Table 1), it is apparent that they generally arise for DNA oligomers with short loop regions, which also exhibit the highest UV melting temperatures, CD spectral profiles indicative of a parallel G4, and broad or crowded NMR imino proton regions. Taken together, these observations support the formation of predominantly higher-order G4 aggregates by these sequences.

In turn, among the bands migrating faster than unstructured single strands two main groups can be distinguished, each with characteristic migration speeds: one already mentioned and faster than the first marker and another slightly faster than the second marker. Their migration speeds fall into regions marked in Fig. 5 as “very fast” and “fast”, respectively. Migrating faster than unstructured oligomers these bands likely correspond to unimolecular G4 structures. Interestingly, in most samples featuring them they were the only major bands present and coincidently only a single dominant form was observed also in 1D NMR. These molecules were thus selected for further 2D NMR analysis of their G4 topologies (see below).

However, many observed bands displayed mobilities similar to or slightly slower than unstructured single strands (“medium” in Fig. 5 and Table 1). In this range, almost a continuum of migration speeds was observed throughout the set making it impossible to draw a clear boundary between regions corresponding to uni- and bimolecular folds. Moreover, these regions may possibly overlap i.e. some compact (sphere-like) bimolecular folds could migrate faster than certain more extended (e.g. disc-like) unimolecular ones.

Another complication that became apparent when analyzing the electrophoretic data was that for many molecules the number of bands differed significantly from the number of spectral forms seen in 1D ^1^H NMR. While in some cases two electrophoretic bands might overlap, this discrepancy is more likely related to the very different oligomer concentrations used by the two methods (400 versus 8 µM). Given the frequency of such discrepancies, multimolecular forms not present in electrophoresis conditions must be often populated in NMR samples and an independent method for their identification was needed to reconcile NMR and native PAGE data.

### G4 molecularity studied by variable-concentration NMR and H-to-D exchange

Symmetric bimolecular G4s are indistinguishable from their unimolecular counterparts in 1D ^1^H NMR spectra based on the number of imino proton signals alone. However, it is well established that G4s with three or more tetrads are usually kinetically stable enough that, upon exchange from an H_2_O- to a D_2_O-based buffer, only the imino protons of their outer tetrads readily exchange to deuterons, while those of central tetrad(s) can remain observable for days or weeks [42]. We therefore examined whether this property could be used to identify multimolecular G4s in 1D NMR spectra of the studied samples. For a unimolecular two-tetrad G4 all imino resonances should disappear upon solvent exchange to D_2_O, while in its bimolecular counterpart (with four stacked G-tetrads) some imino resonances are expected to remain visible.

**Figure 6.**
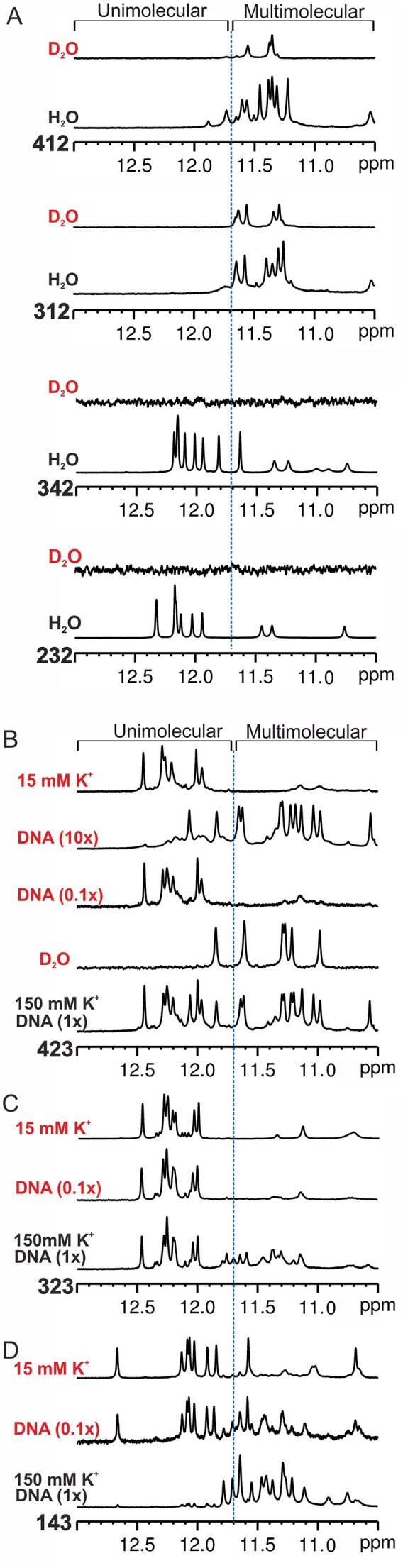
Representative ^1^H NMR spectra collected to determine the molecularity of the studied G4s by H-to-D exchange and by varying DNA and K⁺ concentrations. (**A**) H-to-D exchange experiments for four sequences: for **232** and **342** all imino proton resonances disappear immediately after solvent exchange, whereas in **312** and **412** several persist after 24 h, indicating the presence of uni- and multimolecular G4s, respectively. (**B**–**D**) Representative spectra illustrating the influence of DNA and K⁺ concentrations on the equilibrium between multi- and unimolecular G4s for **423, 323**, and **143**. The vertical line marks the observed chemical shift threshold (11.7 ppm), enabling rapid assessment of molecularity: unimolecular two-tetrad G4s display imino resonances mainly above this line, whereas multimolecular forms resonate predominantly below.

We first applied the H-to-D exchange experiment to the 16 samples that showed a single dominant form in 1D NMR (single set of imino proton resonances; Fig. 4A). In 10 of these samples—**232, 233, 234, 332, 333, 334, 342, 424, 434**, and **442—**all imino resonances disappeared immediately after solvent exchange. In contrast, the remaining six—**123, 212, 312, 321, 322**, and **412—**retained several resonances even the next day (representative examples in Fig. 6A and all data in Supplementary Fig. S7). Reassuringly, all samples in the first group also produced a single fast or very fast migrating PAGE band, consistent with unimolecular G4 structures both at NMR and PAGE concentrations. In the second group, the exchange-protected protons pointed toward a multimolecular fold at NMR concentrations. Under PAGE conditions most of these samples showed multiple bands, including both slow and fast migrating species, suggesting that at lower DNA concentrations some equilibration with folds of lower molecularity must occur (only **312** produces a single band with medium migration speed, suggesting the retention of a single structural form).

Having validated the H-to-D exchange approach, we applied it to the remaining samples that displayed multiple spectral forms in ^1^H NMR (Supplementary Fig. S7). In several cases, only a small fraction of imino protons persisted upon H-to-D exchange (for example **423**, shown in Fig. 6B), suggesting that both uni- and multimolecular forms coexist under NMR conditions. To test this, NMR samples of several such systems were prepared at 10-fold lower and/or higher DNA concentrations (Supplementary Table S2 lists all the tested sequences; all data shown in Supplementary Fig. S1). In many cases, this was sufficient to significantly shift the relative population of the forms (examples in Fig. 6B and C, and all data in Supplementary Fig. S1) and to identify which imino signals correspond to unimolecular folds.

Since G4 structures are metal complexes, and multimolecular forms require higher metal-to-DNA ratios, lowering the salt concentration should preferentially destabilize higher-order structures, producing an equilibrium shift similar to that induced by DNA dilution. NMR spectra recorded at 10-fold lower K^+^ ion concentration confirm this expectation (Fig. 6B and C). For most studied systems changes induced by salt dilution were actually more pronounced, in extreme cases allowing for an almost quantitative transition from multi- to unimolecular G4 folds (Fig. 6C for **143**; similar behavior for **223** and **224** is shown in appropriate panels of Supplementary Fig. S1).

Combining H-to-D exchange experiments with the exploration of different salt and DNA concentrations allowed us to identify the set of sequences capable of forming unimolecular two-tetrad G4s under conditions suitable for 2D NMR studies. A strong and unexpected trend emerged from these experiments: the majority of imino protons belonging to the unimolecular form(s) consistently resonated at higher chemical shifts than their multimolecular counterparts (Supplementary Fig. S6). In each case the bulk of imino signals of multimolecular G4s were located below 11.7 ppm, with at most two signals extending above this threshold (the highest observed for **422** at 12.07 ppm). In contrast, all unimolecular G4 forms displayed multiple imino resonances above 12.0 ppm. Although, to the best of our knowledge, this trend has not been reported before, it is physically well justified, because the chemical shifts of nucleobase protons in structured DNA are strongly influenced by the ring currents from neighboring stacked bases. For two-tetrad G4s, the presence of only one stacking partner results in weaker ring-current-induced shielding and, consequently, in imino protons resonating at the upper edge or even above the typical 10–12 ppm range associated with G4s. This feature can therefore be used to rapidly assess the presence and population of two-tetrad G4s in a sample.

### Assignment of loop topologies for two-tetrad G4s

After identifying the sequences that form unimolecular G4 folds at NMR concentrations, we next focused on determining their G4 topologies according to the classification outlined in Fig. 1. This requires assigning NMR resonances for each molecule and identifying sufficient long-range NOE contacts to unambiguously define the folding pattern. In particular, NOE contacts between imino and aromatic protons in G-tetrads play a crucial role in this process [42]. However, unambiguous assignment of these protons often requires the use of site specific ^13^C/^15^N isotopic enrichment, which is both time- and resource- intensive when applied to multiple DNA sequences. As a result, G4 loop topology assignment is rarely performed in studies involving a broad range of DNA molecules.

**Figure 7.**
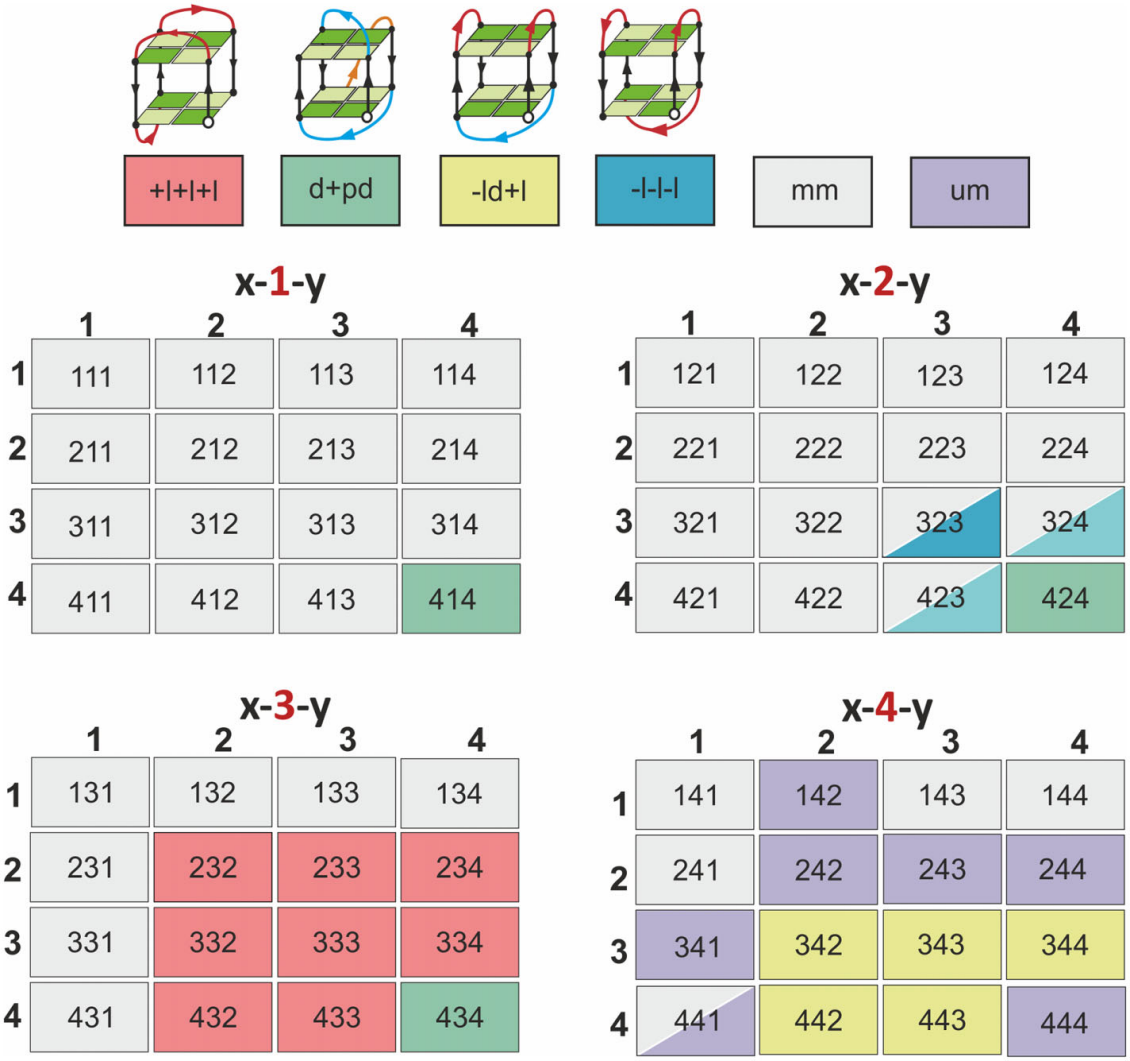
The dominant G4 fold under NMR conditions as a function of the three loop lengths. For unimolecular G4s the assigned loop topology is indicated (if known; otherwise marked as unimolecular, “um”), while multimolecular G4s are grouped into a single category (“mm”, gray). Fields corresponding to sequences that produce comparable populations of uni- and multimolecular species are shown split in half into a gray and colored triangle. Systems for which loop topology assignment is tentative—**324** and **423**—are shown in a lighter shade of the appropriate color.

In the present study, the relative spectral simplicity of two-tetrad G4s allowed us to assign NMR resonances for multiple systems without isotope enrichment (“Materials and methods” section), using standard methods for non-exchangeable protons and phosphorus atoms [37, 38] and ^1^H-^13^C HMBC spectra for imino proton resonances [39]. This, in turn, enabled determination of their topologies based on NOE contacts involving guanosine imino protons (described in detail in the Supplementary data, and illustrated in Supplementary Figs S8 and S9). In total, topologies were assigned for 17 systems: 8 adopting the *+l+l+l* fold, 5 *−ld+l*, 3 *d+pd*, and 1 *−l−l−l* topology (Fig. 7). For systems adopting the *+l+l+l* and -*ld+l* topologies, chemical shift patterns and long-range NOEs between non-exchangeable protons were also compared with previously reported data for two-tetrad G4s of the same folds. The observed features closely match earlier findings (as described in the Supplementary data and illustrated in Supplementary Fig. S10 and Supplementary Table S3), providing an additional confirmation of the assigned topologies.

## Discussion

### Structural preferences of two-tetrad G4s as a function of loop lengths

Our aim was to investigate the sequence-topology relationship for two-tetrad G4s with thymidine-only loops using a comprehensive set of DNA sequences. By combining multiple experimental approaches (see the “Results” section) we determined which molecules form two-tetrad G4s and which preferentially fold into higher order G4 assemblies. In many cases, the transition from PAGE to NMR conditions significantly shifted the equilibrium between these structural forms. Accordingly, the “Discussion” section focuses primarily on NMR conditions, for which more detailed structural information was obtained, while also referring to low-concentration behavior where relevant.

When the dominant G4 fold is analyzed as a function of the three loop lengths, the clearest overall trends emerge when the central loop length is treated as the primary variable. Figure 7 presents the obtained results in this format with each of its four panels corresponding to a different length of the central loop. Similar tables focusing on the first and the third loops are provided in Supplementary Fig. S11.

#### Systems containing single nucleotide loops

When the central loop consists of a single nucleotide, multimolecular G4 structures dominate for almost all combinations of the other two loop lengths (see Fig. 7). The only exception is sequence **414**, which forms a two-tetrad G4 of *d+pd* topology. This trend also applies to molecules with single nucleotide loops at other positions: of 37 sequences containing such loops, only 3—**414, 142**, and **341**—display dominant unimolecular populations under NMR concentrations. For some sequences—notably **141, 143, 241**, and **331**—minor populations of unimolecular G4s can also be detected in NMR spectra, which can be further enhanced by adjusting experimental conditions (as illustrated in Fig. 6D for **143**). More than a dozen additional sequences of this type may partially fold into unimolecular G4s at significantly lower DNA concentrations used in PAGE, as indicated by intense, fast migrating bands observed, for example for **113, 114, 123**, or **131**. However, except for **123** (Supplementary Figs S1–S7), we were not able to induce even minor populations of unimolecular forms under NMR conditions. Some sequences, such as **111**, remain exclusively multimolecular even at PAGE concentrations. Because multimolecular assemblies dominate under NMR conditions, it is difficult to draw conclusions regarding the topologies of two-tetrad G4s formed by this group of molecules.

Concurrently, it is noteworthy that nearly all sequences producing “parallel” or “mostly parallel” CD spectra (Fig. 3) contain single-nucleotide loops. In no case, however, can this CD signature be confidently assigned to a unimolecular, two-tetrad G4. While some of these sequences (e.g. **123**) may form parallel unimolecular G4 under PAGE conditions, their conformational ensemble under CD conditions remains uncertain. These observations are consistent with previous reports [31] showing that DNA with GG tracts and short loops preferentially form multimolecular assemblies rather than parallel two-tetrad G4s, a trend further reinforced by our results.

#### Systems with a two-nucleotide central loop

**Figure 8.**
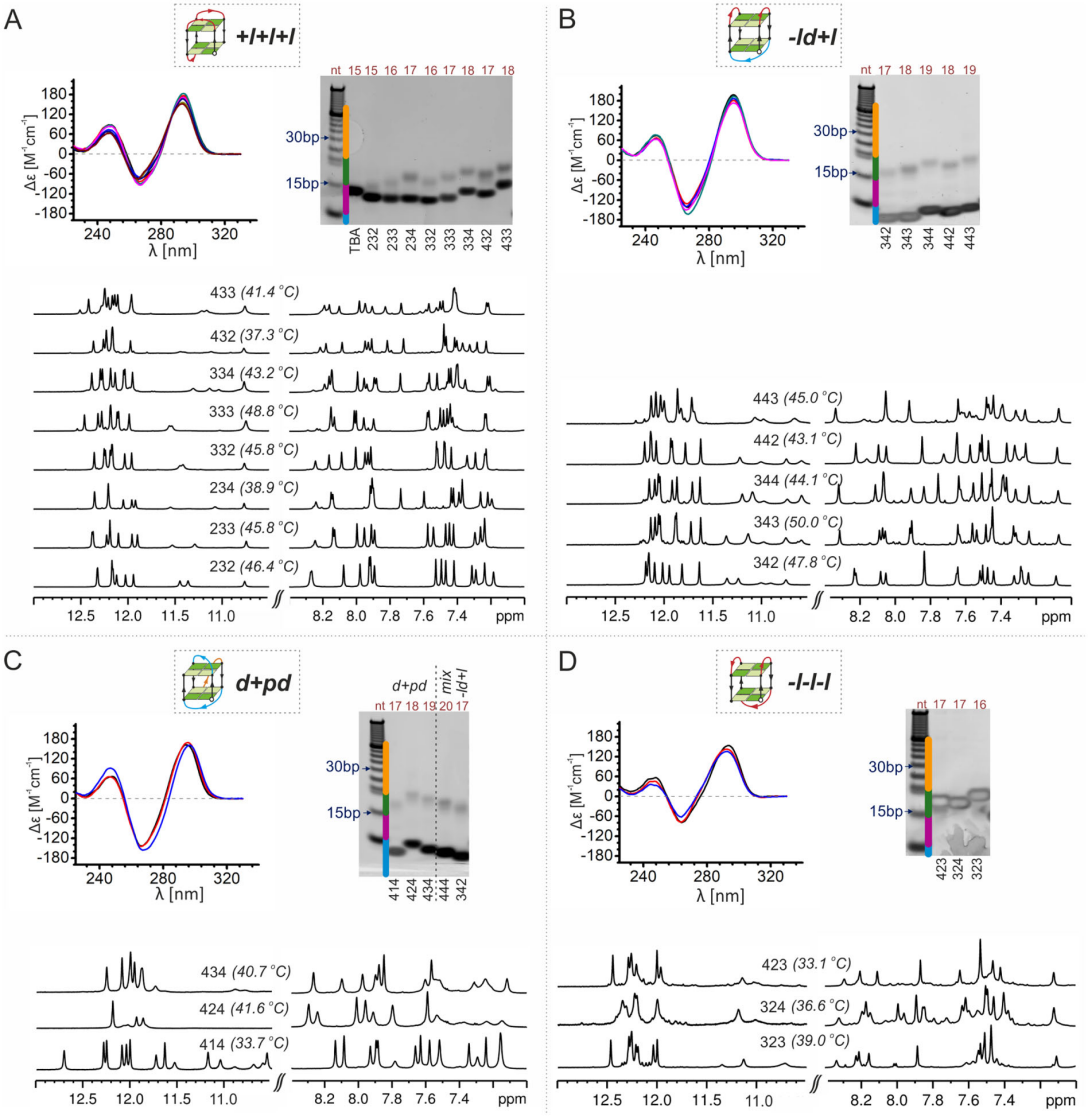
Collected 1D ^1^H NMR, CD, electrophoretic mobility, and thermal stability data characterizing each of the four G4 loop topologies identified in this study. (**A**) *+l+l+l*, (**B**) *−ld+l*, (**C**) *d+pd*, and (**D**) *−l−l−l*. In panel (C) two additional sequences, not assuming the *d+pd* topology, are shown in the gel fragment for comparison (see text).

For systems inf which the central loop contains two nucleotides, a clear boundary is apparent in the sequence space between regions dominated by multimolecular and unimolecular G4 structures. When either the first or the third loop has fewer than three nucleotides, multimolecular folds predominate under our standard NMR conditions (0.4 mM DNA, 150 mM KCl), whereas at the opposite extreme, **424** sequence exists exclusively in the unimolecular *d+pd* form. Three sequences located in the transition region between these extremes—**323, 324**, and **423**—exist in an equilibrium between multi- and unimolecular G4s. This equilibrium can be shifted almost quantitatively in either direction within the concentration range required for 2D NMR measurements (Fig. 6B and C). Among these three systems, **323** was shown by 2D NMR analysis to adopt the *−l−l−l* topology. For the remaining two sequences, this approach was inconclusive, however, their highly similar 1D NMR spectra, together with their shared, characteristically slow gel migration (Fig. 8D), strongly suggest that they also adopt the *−l−l−l* topology.

While not significantly populated under our standard NMR conditions, unimolecular folds can also be induced for sequences **222, 223, 224**, and **322** at lower K^+^/DNA concentrations (Supplementary Fig. S1). However, their topologies could not be determined in such conditions.

#### Systems with a three-nucleotide central loop

When the central loop consists of three nucleotides, a single unimolecular G4 fold clearly dominates, namely the *+l+l+l* topology. The corresponding region of the sequence space can be defined as 5′-GG**T**_**2–4**_GG**T**_**3**_GG**T_2–4_**GG-3′, with a single exception—**434**—which instead adopts the *d+pd* topology. The **232** sequence represents the root of this region and closely resembles TBA oligonucleotide for which the *+l+l+l* topology was first reported [26]. The results presented here show that lengthening of the first and/or the third loop preserves this fold. Moreover, increasing loop length initially has little effect on the thermal stability of the *+l+l+l* topology, with the **333** sequence exhibiting the highest melting temperature among the group. Only when either loop reaches four nucleotides, the G4 structures become somewhat less stable. On the other hand, modifying the length of the central loop or shortening any other loop to a single nucleotide leads to replacement of the *+l+l+l* topology by other G4 forms.

All eight systems adopting the *+l+l+l* fold share a common gel mobility, distinct from that of other unimolecular G4 topologies identified in the dataset (see below). Interestingly, the **132** and **231** oligonucleotides, which form mixtures of multimolecular G4s under our standard NMR conditions, also give rise to similarly migrating gel bands. These results suggest that the *+l+l+l* topology may also be available to some G4s with single-nucleotide loops, although multimolecular folds are preferred at higher DNA and KCl concentrations.

#### Systems with a four-nucleotide central loop

Finally, when the second loop contains four nucleotides, a single unimolecular G4 topology dominates—this time the *−ld+l* fold—although less clearly than the *+l+l+l* in the previous category. The five sequences for which this topology was unambiguously confirmed all follow the pattern 5′-GG**T_3–4_**GG**T_4_**GG**T_2–4_**GG-3′. Among them, **343** forms the most stable G4 structure, yet all five systems exhibit melting temperatures above 40°C. Six additional oligonucleotides may also populate the *−ld+l* fold, but NMR analysis was not conclusive. For both **142** and **341** a single unimolecular G4 form predominates, however, limited thermal stability of these structures prevented successful topology assignment. For the remaining sequences **242, 243, 244**, and **444** the recorded 2D NMR spectra were too crowded for a conclusive analysis due to the presence of multiple unimolecular G4 species. Nevertheless, the NOESY spectra of all these systems show a chemical shift and NOE patterns similar to those observed by us and others [35] for a four-thymidine diagonal loop in the *−ld+l* fold (described in the Supplementary data). Thus, all of them appear to feature a G4 topology with a central diagonal loop in their conformational ensemble, although its exact identity could not be unambiguously confirmed.

#### Summary of the observed trends

In summary, the observations described above unveil a dominant role of the central loop length in determining the preferred molecularity and topology of the studied molecules. Another major factor influencing the molecularity of the observed G4s appears to be the presence of a single-nucleotide loop at any position, which strongly promotes the formation of multimolecular species.

The only clear exception from these trends is the unimolecular *d+pd* topology, which dominates for oligomers of the type 5′-GG**T_4_**GG**T_1–3_**GG**T_4_**GG-3′. This topology is preferred when both the first and third loops are four nucleotides long, regardless of the central loop length, and it persists even when the central loop consists of a single nucleotide. Notably, the *d+pd* topology is the only one (see Fig. 1B) that contains a diagonal loop located outside the central position (in fact, two such loops). Previous studies suggests that loops composed of four thymidines preferentially adopt the diagonal conformation [19]. It can therefore be proposed that when two such loops flank the central one, their common propensity to assume the diagonal conformation can outweigh the otherwise dominant conformational preferences imposed by the central loop. An interesting case is provided by the **444** sequence, which lies at an intersection between regions of the loop-length space occupied by the *−ld+l* and *d+pd* topologies. This molecule forms an ~1:2 mixture of two unimolecular G4s (Supplementary Fig. S1_64). Although the spectral complexity of this mixture precluded unequivocal identification of the individual forms, the **444** sequence produces a single native gel band with a migration rate shared by the -*ld+l* and *d+pd* topologies. This system may thus represent a “balance point” between these two folds, which could be tipped in either direction by, for example, base substitutions in the loops (see discussion of the previously reported [25] *C. elegans* telomeric G4 structure provided below).

### New methodological insights

This work, representing the most comprehensive analysis of two-tetrad G4 structural preferences to date, uncovers several experimental trends not previously reported:

- Imino protons of two-tetrad G4s occupy a chemical shifts range somewhat distinct from that of G4s with more tetrads

As discussed in the “Results” section, all two-tetrad G4s in our set displayed multiple imino resonances above 12.0 ppm, while for multimolecular G4s the bulk of imino signals resonated below 11.7 ppm. This feature could be exploited in future studies to rapidly confirm the formation of two-tetrad G4s, potentially in combination with an H-to-D exchange experiment, which in turn can provide a rapid confirmation of the presence of more complex G4 states.

- Electrophoretic mobility of different antiparallel two-tetrad G4 loop topologies varies significantly, with some migrating similarly to multimolecular species

Another new observation from this study is the unexpectedly large variation in gel migration between unimolecular G4s of different antiparallel topologies. Sequences adopting the *−ld+l* and *d+pd* folds exhibit the fastest migration, while those with *+l+l+l* topology migrate significantly more slowly, although still faster than unstructured single strands. Sequences with *−l−l−l* topology migrate even more slowly than the unstructured form, giving rise to three clearly distinct migration classes for unimolecular anti-parallel G4s. To the best of our knowledge such a pronounced influence of G4 loop topology on its electrophoretic mobility has not been reported previously. Slower gel migration was mainly linked to propeller loops, especially in parallel G4s where all three loops adopt this geometry, whereas none of the topologies identified here contain such motifs [43]. The relatively small size of systems studied here may have contributed to the better differentiation of gel migration speeds compared to G4s with more tetrads.

- Antiparallel two-tetrad G4s give rise to two distinguishable types of CD profiles that correlate with their loop topologies

Finally, analysis of the CD spectra revealed two distinct subgroups within the antiparallel category, differing in the intensity of the negative peak around 265 nm (Fig. 3). Assignment of loop topologies for many of these sequences showed a clear correlation between spectral subgroups and specific topologies. G4s adopting the *−ld+l* and *d+pd* topologies produce “antiparallel 1”-type spectra, while those with *+l+l+l* and *−l−l−l* topologies account for the majority of the “antiparallel 2” group. One explanation for this finding may be that loop regions can noticeably influence the shape of CD spectra of two-tetrad G4s, in contrast to what was previously proposed for G4 folds featuring more tetrads [44]. This difference could arise because the CD signal of two-tetrad G4 core is inherently weaker than that of G4s with more tetrads, making the contribution of the loop regions more pronounced. Coincidently however, a new classification of G4 folds was recently published [45], distinguishing eight G4 types, based on the properties of their guanosine cores i.e. relative strand directionalities, as well as the glycosidic angle and groove-width patterns that they give rise to (in contrast to the commonly used classification based on loop progressions given in Table 1). In this new system, the *−ld+l* and *d+pd* loop topologies collapse into a single class named “Antiparallel-basket,” while similarly *+l+l+l* and *−l−l−l* topologies form the “Antiparallel-chair” class. The clear correspondence between “antiparallel 1”/“antiparallel 2” CD profiles and “Antiparallel-basket”/ “Antiparallel-chair” classes opens up the possibility that these two types of antiparallel G4 cores themselves give rise to slightly different CD profiles (due to slightly different geometries of GG stacking) potentially making different classes of antiparallel G4 cores distinguishable in CD spectroscopy.

The distinct behavior of different antiparallel two-tetrad G4 topologies in gel electrophoresis and CD spectroscopy allows some of them to be rapidly identified using a combination of low-resolution methods alone. For example, in the current dataset an “antiparallel 2” CD spectrum combined with intermediate gel migration was unique to the *+l+l+l* topology, while the same CD profile coupled with slow migration identified the *−l−l−l* topology. On the other hand, the *−ld+l* and *d+pd* topologies both exhibit an “antiparallel 1” CD spectrum and fast gel migration. Thus, while these two topologies are clearly distinguishable from *+l+l+l* and *−l−l−l*, they cannot be differentiated from each other based on these data alone.

### Comparison of the new results with current state of knowledge

A search within the PDB database reveals that only seven distinct topologies have been reported to date for two-tetrads G4s (Table 2). Sequences giving rise to each of these topologies will be discussed and compared with their closest counterparts in the present study.

**Table 2.** Loop topologies of previously reported two-tetrad G4 structures compared to their closest counterparts in the current work

	Previously characterized sequences					Counterpart in our dataset	
	Sequence (5′/3′)	PDB ID/ reference	Loop lengths	Topology	Comment	Seq ID	Topology
1	GG **TTT** GG **TTTT** GG **TT** GG	5j4w [35]	3-4-2	*−ld+l*	Na^+^	**342**	*−ld+l*
2	GG **TTT** GG **TTTT** GG **TTT** GG	5j4p [35]	3-4-3	*−ld+l*	Na^+^	**343**	*−ld+l*
3	GG **GTTG** GG **TTTT** GG **GT** GG G	2m6v [35]	4-4-2	*−ld+l*	Na^+^	**442**	*−ld+l*
4	GG **GTTA** GG **GTTAG** GG **TTA** GG GT	2kf8 [23] 2kf7 [23]	4-5-3	*−ld+l*	G-triple	**443**	*−ld+l*
5	GG **GTA** GG **GAGCG** GG **AGA** GG G	6gzn [46]	3-5-3	*−ld+l*	Two base triples	**343**	*−ld+l*
6	GG **CTTA** GG **CTTA** GG **CTTA** GG	7oqt [25]	4-4-4	*−ld+l*	C-T pair	**444**	mix*
7	TA GG **GTTA** GG **GTTAG** GG **TTA** GG	5lqg [15] 8jic [47]	4-5-3	*−ld+l*	Base triple with neutral A20	**443**	*−ld+l*
8	TA GG **GTTA** GG **GTTAG** GG **TTA^+^** GG	5lqh [15] 8jih [47]	4-5-3	*+ld−l*	Base triple with protonated A20^+^	**443**	−ld+l*
9	TA GG **GTA** GG **GTAG** GG **TAI** GG	2kow [48]	3-4-3	*+ld−l*	Two base triples	**343**	−ld+l*
10	GG **TT** GG **TGT** GG **TT** GG	e.g. 148d [26]	2-3-2	*+l+l+l*	-	**232**	*+l+l+l*
11	GG **CGA** GG **AGG** GG **CGT** GG CCGGC	6gh0 [49]	3-3-3	*−l−l−l*	G-C base pair (involving flanking region)	**333**	+l+l+l*
12	GG **TTTT** GG **CA** GG **GTTTT** GG T	1i34 [50]	4-2-5	*d+pd*	Na^+^, G-T pair	**424**	*d+pd*
13	TG GG **TTTG** GG **TTG** GG **TTT** GG G	2mfu	4-3-3	*−l−l−p*	Na^+^, G-G pair	**433**	+l+l+l*
14	GG**A**GG**A**GG**A**GGA	1myq [51]	1-1-1	*−p−p−p*	Dimer	**111**	-^a^

^a^A multimolecular parallel fold.

Instances in which the two topologies differ are marked with a star. The presence of additional structural elements or an ion different than K^+^ in the literature structures is also indicated.

The *−ld+l* topology is found in the PDB for a variety of loop lengths and compositions and was previously reported for the exact counterparts of **342** and **343**, albeit in the presence of Na^+^ ions (Table 2, rows 1–2) [35]. Our results show that for these sequences the same topology is also induced by K^+^ ions. The same study also described a two-tetrad *−ld+l* G4 with loop lengths of 4-4-2, but containing both T and G residues (Table 2, row 3) [35]. Its closest equivalent in our dataset, **442**, also adopts this topology. Additionally, the *−ld+l* topology was also observed for oligomers derived from human telomeric repeats (Table 2, row 4) [23] and from the regulatory region of RANKL gene (Table 2, row 5) [46]. In both cases, the central loop consists of five nucleotides, so these sequences have no direct counterparts in our dataset. Nevertheless, the most closely related sequences studied here—**443** and **343**, respectively—also adopt the *−ld+l* fold. This apparent agreement may be coincidental, however, as both the telomeric and RANKL G4s feature extensive interactions within their loop regions, including base-triple formation. These additional interactions are likely to significantly influence the energetics of these systems and their preferred G4 topologies, as illustrated by the next examples. The *C. elegans* telomeric repeats quadruplex, which contains three CTTA loops, also adopts the *−ld+l* topology (Table 2, row 6) [25]. In contrast, the **444** oligonucleotide in our dataset exists as a mixture of two unimolecular antiparallel G4s. One of these states might be -*ld+l* fold, as two of the closest sequence neighbors of **444** (**344** and **443**) adopt this topology, but this could not be confirmed. Interestingly, a mutational analysis of the *C. elegans G4* [25] revealed that certain single nucleotide substitutions within the CTTA loops (namely C15T and T17C) led to the formation of a mixture of two antiparallel G4s, similar to what we observe for **444**, highlighting the importance of interactions within the loops for G4 conformational preferences. A particularly striking example of this phenomenon is provided by two-tetrad G4s formed by another oligomer derived from human telomeric repeats (Table 2, rows 7 and 8) [15]. In this system, the protonation state of an adenosine in the third loop dictates the preferred G4 topology: the *−ld+l* fold is favored at neutral pH, while *+ld−l* fold dominates when the adenosine is protonated at lower pH. Interestingly, both structures feature extensive hydrogen bond interactions in their loops, with the N1 nitrogen of the crucial adenosine acting as either an H-bond acceptor or donor depending on its protonation state. Apart from the discussed structure with a protonated adenosine, the *+ld−l* topology was reported in only one other context—namely, the *Giardia* telomeric G4 with 3-4-3 loop lengths (Table 2, row 9) [48]. This structure contains two purine triples flanking the G4 core, which are likely responsible for selecting the *+ld−l* topology over *−ld+l* fold observed for **343** in our dataset.

Another topology frequently encountered among two-tetrad G4s in the PDB database is *+l+l+l*. However, nearly all reported instances correspond to TBA, its chemically modified variants, or their complexes with thrombin (representative example [26] given in Table 2, row 10). The closest counterpart of TBA in our dataset—**232**, with only a single G-to-T substitution—also adopts the *+l+l+l* topology. Our results (Fig. 8A) further show that G- quadruplexes with elongated first and/or third loops retain this fold, although no structures of TBA variants with similar loop lengths are available for comparison. In contrast, a two-tetrad G4 structure derived from the c-KIT promoter (kit*) with 3-3-3 loop lengths adopts the *−l−l−l* topology instead (Table 2, row 11) [49]. This discrepancy is likely attributable to the formation of additional structural elements involving loop regions. In this case, a guanosine from the central loop forms a Watson–Crick base pair with a cytidine from the 3′ flanking region. Accordingly, truncation of this flanking sequence was shown to significantly affect the conformational preferences of kit* [49].

Similarly to *−l−l−l*, the next two topologies are each represented only once in the PDB. The *d+pd* topology was reported in the presence of Na^+^ ions for an oligomer with 4-2-5 loop lengths (Table 2, row 12) [50]. The same topology was observed in our study for its closest counterpart, **424**. In turn, the *−l−l−p* topology is found in the PDB for a G4 with 4-3-3 loop lengths and a G-G base pair on the top of 3′-G-tetrad (Table 2, row 13). This represents the only reported instance of a hybrid-type topology for a two-tetrad G4 to date. Unfortunately, the publication describing this PDB entry is not yet available, making it difficult to speculate on factors driving the formation of this structure.

Finally, the parallel topology, *−p−p−p*, has also been reported for several systems nominally containing only two G-tetrads. However, in each case the structure either consists of a dimer of two-tetrad G4 units stacked on top of each other, effectively forming a four-tetrad core (e.g. Table 2, row 14) [51], or represents a derivative of a three-tetrad G4 in which one guanosine has been removed, resulting in a G-triple in addition to the two G-tetrads, making it more akin to a three-tetrad structure [31]. Interestingly, a study aimed at identifying a unimolecular two-tetrad parallel G4 lacking extensive stabilizing loop interactions concluded that such systems are intrinsically unstable, leading instead to the formation of multimolecular G4 assemblies [31]. This is in agreement with our current dataset, in which all sequences that produce parallel type CD spectra were found to form predominantly multimolecular G4s.

As a concluding remark, it is worth noting that the described set of available unimolecular two-tetrad G4 structures shows a remarkable scarcity of folds featuring propeller loops—only the *d+pd* and *−l−l−p* topologies were each reported once in the literature, with our dataset containing three additional instances of the former. This is particularly interesting because propeller loops uniquely span the full height of the G4 core (whereas lateral and diagonal loops typically stack on outer tetrads), and their energetics are therefore expected to depend strongly on the number of tetrads in the core. In contrast, propeller loops are abundant in reported three-tetrad G4 structures with comparable loop lengths. For example, multiple studies on oligomers derived from human telomeric repeats (TTAGGG)_n_ have reported three-tetrad G4s with 3-3-3 loops adopting *−l−l−p, −p−l−l*, or −*p−p−p* topologies in the presence of K⁺ ions [52, 53]. Similarly, the *−l−l−p* topology was observed for a three-tetrad G4 derived from the *Tetrahymena* telomeric repeat containing 4-3-2 loops (GTTG–TTG–TT) [54]. Curiously, both **414** from our dataset and its direct three-tetrad counterpart featuring all-thymidine loops [35] were found to adopt the same *d+pd* topology, which is notably one of the few examples of a propeller loop observed even in two-tetrad G4s. However, more structural data are needed to determine whether the rarity of propeller loops in two-tetrad G4s is a statistical artifact or does if reflect a genuinely reduced propensity to form this structural element.

## Conclusions

Overall, this work provides a comprehensive experimental dataset that advances the understanding of both the formation propensity and topological preferences of two-tetrad G4s. Our results demonstrate that, except for sequences containing single-nucleotide loops, the studied DNA sequences form stable two-tetrad G4s, with topology primarily dictated by the central loop length.

The presented dataset describes properties of G4-forming DNA sequences with loops composed entirely of thymidine residues and lacking 5′- or 3′-flanking residues. These two factors have to be kept in mind when generalizing the trends reported here to the broader set of two-tetrad G4-forming oligonucleotides. Thymidine residues do not typically form particularly stable homo base pairs and are likely to stack relatively poorly on the G4 core compared to other nucleobases [55]. Loop compositions that enable the formation of more extensive networks of interactions, including base pairs or triples, can significantly alter the conformational preferences of a two-tetrad G4. Data presented in Table 2 illustrate the extent of this phenomenon among two-tetrad G4 structures solved to date, with the additional loop interactions highlighted and each instance described in the “Discussion” section. Thus, the conformational preferences observed in the current study can be regarded as a reasonable approximation of the energetic contribution of loop length alone, providing a valuable reference point, on top of which the effects of specific intraloop interactions have to be considered when predicting the conformational preferences of two-tetrad G4s with arbitrary loop compositions. The other crucial factor to consider is the influence of flanking sequences, which are almost always present in genomic G4-forming sequences. In some instances, their effect arises from specific interactions with residues within the G4 loops, as is the case for the already discussed kit* G4 [49]. However, a more general—sequence independent—observation was also reported, showing that the presence of flanking sequences tends to favor parallel G4 folds by reducing the propensity of the 5′-terminal guanosine to adopt the *syn* conformation, commonly found in non-parallel G4s [56]. To what extent this effect also applies to two-tetrad G4s—for which a monomeric, parallel fold was not reported thus far [31]—remains to be established. On the other hand, it is well-known that the presence of flanking residues significantly reduces the propensity of G4s for multimerization [57]. The formation of multimolecular folds was quite common within the set of sequences studied here, especially among those containing the shortest loops. Thus, future studies on models including flanking residues may reveal that the region of the loop-length space favoring unimolecular G4 formation is even broader than what the current work reports.

## Supplementary Material

gkag590_Supplemental_File

## Data Availability

The data underlying this article are available in the article and in its online Supplementary material. Raw experimental data were also deposited in Zenodo repository under the DOI: 10.5281/zenodo.19248269. NMR chemical shift assignments for sequences **232, 233, 234, 332, 333, 334, 432, 433, 342, 343, 344, 442, 443, 434, 424, 414**, and **323** were deposited at BMRB under accession codes 53681, 53682, 53683, 53684, 53685, 53686, 53687, 53688, 53689, 53690, 53691, 53692, 53693, 53694, 53695, 53696, and 53697, respectively.
