# Supplementary material for: How loop lengths shape topological preferences of two-tetrad G-quadruplexes
Source: Nucleic Acids Res. 2026 Jun 16;54(11):gkag590. doi: 10.1093/nar/gkag590 (PMC13270197; doi:10.1093/nar/gkag590)
Supplement: gkag590_Supplemental_File [file gkag590_supplemental_file.pdf]

Supplementary information

How loop-lengths shape topological preferences of two-tetrad G-quadruplexes

Amadeusz Woś, Karolina Zielińska, Karol Pasternak, Aleksandra Pawłowicz-Perczak, Zofia Gdaniec, Dorota Gudanis-Sobocińska,* Witold Andrałojć,*

Institute of Bioorganic Chemistry, Polish Academy of Sciences, 61-704 Poznan, Noskowskiego 12/14, Poland

* To whom correspondence should be addressed. Email: wandralojc@ibch.poznan.pl,

Correspondence may also be addressed to Dorota Gudanis-Sobocińska. Email: dgud@ibch.poznan.pl

Table of contents:

- **Supplementary Note 1.** G4 topology assignment methodology

- **Supplementary Figure S1_1-64**. Main experimental results (UV melting, CD spectroscopy, 1D ^{1}H NMR spectroscopy and gel electrophoresis) for each of the 64 sequences studied (one panel per sequence).

- **Supplementary Figure S2**. UV melting curves (measured at 295 nm) of the 64 sequences studied.

- **Supplementary Figure S3**. The origin of Circular Dichroism spectral shapes for G4 structures.

- **Supplementary Figure S4**. Circular Dichroism spectra of the 64 studied sequences.

- **Supplementary Figure S5**. Principal component Analysis (PCA) of the obtained set of 64 CD spectra.

- Supplementary Figure S6. Imino proton regions of 1D ^{1}H NMR spectra of 64 studied molecules

- Supplementary Figure S7. Imino proton regions of 1D ^{1}H NMR spectra of the studied set of 64 molecules measured after H_2O to D_2O solvent exchange.

- **Supplementary Figure S8.** Assignment of G4 topologies based on 2D NMR data.

- **Supplementary Figure S9.** Expected imino-to-imino and imino-to-aromatic NOEs for each of the 14 possible G4 topologies.

- **Supplementary Figure S10.** Illustration of ^{1}H and ^{31}P chemical shifts differences between **232** and TBA.

- **Supplementary Figure S11**. The dominant G4 fold under NMR conditions as a function of the three loop lengths.

- **Supplementary Table S1**. Classification of the studied molecules based on the imino regions of 1D ^{1}H NMR spectra.

- **Supplementary Table S2**. List of sequences for which 1D NMR experiments at different DNA and/or KCl concentrations were performed.

- **Supplementary Table S3**. ^{1}H and ^{31}P chemical shifts of TBA measured at 20 °C.

- **Supplementary References.**

Supplementary Note 1. *G4 topology assignment methodology*

The most reliable method to derive the topology of a G4 fold from NMR spectra, without explicitly solving its high-resolution structure makes use of NOE contacts between imino and aromatic protons within G-tetrads, as well as similar contacts between imino protons of neighboring G-tetrads. The pattern of expected imino-to-aromatic and imino-to-imino NOE contacts is distinct for each topology, as shown in Supplementary Figure S9. A comparison between the set of NOEs observed experimentally for a given G4 structure and the blueprint given in the abovementioned figure allows to unambiguously confirm the G4's topology, provided that enough diagnostic NOEs could be identified in the NMR spectra.

To use the imino-to-aromatic and imino-to-imino NOE contacts in this way, it is first necessary to obtain the resonance assignment of the imino (H1) and aromatic (H8) protons of all guanosine residues forming the G-tetrads. For this, end site specific $^{13}C/^{15}N$ isotopic enrichment is often used, yet this method is both time and resource intensive to apply to multiple DNA sequences. Luckily, for many of the two tetrad G4s studied here, the short oligomer length resulted in relatively simple spectra, allowing assignment of non-exchangeable proton resonances (including guanosine H8 atoms) using standard sequential assignment methods for nucleic acids. These methods rely on ^{1}H-^{31}P through-bond connectivities between consecutive residues in conjunction with ^{1}H-^{1}H NOESY connectivities to identify protons belonging to the same residues.

To obtain the guanosine imino proton assignments, ^{1}H-^{13}C HMBC spectra were recorded that connect the imino H1 and aromatic H8 resonances of the same guanosine through correlation with the C5 carbon atom.[1] Thus, using ^{1}H-^{13}C HMBC spectra the knowledge of H8 proton assignments was leveraged to uncover the identities of H1 imino protons within the G-tetrads.

The whole procedure: (1) non-exchangeable proton and ^{31}P resonance assignment, (2) imino proton assignment and (3) topology identification - was successfully completed for 17 molecules within the studied dataset. Each entry is documented in the appropriate panel of Supplementary Figure S8 (numbered using Roman numerals I – XVII). Each panel contains:

- a schematic representation of the identified topology and expected NOE connectivities (with residue numbering adjusted for each sequence) along with figures depicting the

assigned ^{1}H-^{13}C HMBC spectrum and the assigned imino-to-aromatic and imino-to-imino NOE regions of the ^{1}H-^{1}H NOESY spectrum **- top part**

- the aromatic-to-anomeric region of the ^{1}H-^{1}H NOESY spectrum and the full ^{1}H-^{31}P COSY spectrum - **middle part**
- the resonance assignment table - **bottom part**:

The panel numbers corresponding to specific sequences are as follows:

Sequence	Panel number in Supplementary Figure S8	Experimental conditions	Determined topology
**232**	I	20 °C, 150 mM K^{+}	*+l+l+l*
**233**	II	20 °C, 150 mM K^{+}	*+l+l+l*
**234**	III	20 °C, 150 mM K^{+}	*+l+l+l*
**332**	IV	20 °C, 150 mM K^{+}	*+l+l+l*
**333**	V	20 °C, 150 mM K^{+}	*+l+l+l*
**334**	VI	20 °C, 150 mM K^{+}	*+l+l+l*
**432**	VII	20 °C, 150 mM K^{+}	*+l+l+l*
**433**	VIII	20 °C, 150 mM K^{+}	*+l+l+l*
**342**	IX	20 °C, 150 mM K^{+}	*-ld+l*
**343**	X	20 °C, 150 mM K^{+}	*-ld+l*
**344**	XI	20 °C, 150 mM K^{+}	*-ld+l*
**442**	XII	20 °C, 150 mM K^{+}	*-ld+l*
**443**	XIII	20 °C, 150 mM K^{+}	*-ld+l*
**414**	XIV	15 °C 75 mM K^{+}	*d+pd*
**424**	XV	35 °C 150 mM K^{+}	*d+pd*
**434**	XVI	35 °C 150 mM K^{+}	*d+pd*
**323**	XVII	20 °C, 60 mM K^{+}	*-l-l-l*

For some molecules, the observed chemical shifts and NOE patterns could be compared with NMR data reported in the literature for closely related systems, providing a secondary means of confirming their G4 topologies. Such comparisons, together with

additional spectral features characteristic of each topology identified in the studied set, are presented in the following sections.

G4 topology assignment – additional spectral patterns observed for *+l+l+l* G4s

The discussion of *+l+l+l* topology in our dataset is best started from the simplest example – the **232** oligomer. It is closely related in sequence to the best characterized *+l+l+l* G4 in the literature – the thrombin binding aptamer (TBA), from which it differs by only a single G8-to-T8 substitution in the central loop. Thus, if **232** indeed adopts the *+l+l+l* topology, its chemical shift values can be expected to closely resemble those of TBA for all residues not directly interacting with the central loop. To verify this, we reassigned TBA chemical shifts in our buffer (Supplementary Table S2) and compared them to those of **232** (Supplementary Figure S10). As shown in Supplementary Figure S10, almost identical chemical shifts were observed between TBA and **232** for residues belonging to loops 1 and 3, as well as, for the G-tetrad adjacent to them. In the central loop, the G8-to-T8 substitution did cause significant chemical shift changes (Supplementary Figure S10); however, the conformation of this loop likely remains similar in both TBA and **232**, based on the observed sequential and long-range NOE patterns. In TBA, the second and third residues of the central loop, G8 and T9, stack with the adjacent G-tetrad giving rise to a set of non-sequential NOE contacts: T9H1'-G15H8, G8H8-G6H1' and G8H8-G6H8. In addition, a chain of sequential H1'(n)-H8(n+1) NOEs is observed for residues G6-T7-G8-T9 and broken for the T9-G10 pair. The same NOE pattern is observed for **232**.

All other *+l+l+l* G4s in our dataset also contain a three nucleotide TTT central loop and differ from each other only by the lengths of the first and/or third loops. Because the central loop in the *+l+l+l* topology interacts with the opposite side of the G4 structure relative to the other two loops, its conformation and chemical environment are expected to be only marginally affected by the changes in the first or third loops. Consistent with this expectation, the NOE pattern described above is observed for each of these systems. The chemical shifts values measured in the G6-T7-T8-T9-G10 region (numbering according to **232** sequence) are remarkably similar between the eight studied *+l+l+l* G4s, with all proton resonances agreeing within 0.1 ppm – except for T8H1' – which exhibit a slightly broader range of 0.138 ppm.

Regarding the first and third loops, solution structures of TBA show that their conformations mirror each other, with the first thymidine of each loop exposed to the solvent, while the second stacks on the adjacent G-tetrad. This interaction is reflected most prominently in chemical shift values of the involved thymidine methyl groups (significantly upfield shifted to around 1.0 ppm) and in their NOE contacts (prominent NOEs to G(n-2) H8 and H1' resonances). Similar chemical shift values and NOEs were observed for T4 and T13 methyl groups of **232,** as expected, because its sequence is identical to TBA in this region of the structure. Lengthening of the first and/or third loops in the other *+l+l+l* G4s is expected to reshape their local conformation. Interestingly, however, for each studied system we still observed two thymidine methyl groups resonating around 1.0 ppm giving rise to NOE contacts described above with the preceding guanosine residue. In all cases, these features were associated with the terminal thymidine of each loop, suggesting that regardless of the loop-length, it is always the last residue that stacks on the G4 core. The presence of similar stacking interactions for all studied *+l+l+l* G4s may partially account for their comparable thermal stabilities.

G4 topology assignment – additional spectral patterns observed for *-ld+l* G4s

Oligomers **342** and **343** were previously reported to adopt the *-ld+l* topology in presence of Na^+ ions.[2] As the same topology was also assigned here in the presence of K^+ ions, chemical shift values under both conditions were compared. For most non-exchangeable protons the values observed in the two conditions agreed reasonably well (within 0.2 ppm), despite different buffer conditions. However, some notable exceptions were observed, such as H3' protons from residues T3, G6, and T10, which were reported significantly upfield shifted - below 4.5 ppm - in the literature, but resonated within the typical H3' range in our samples. The origin of these discrepancies is difficult to identify, especially given a close reproduction of other rare chemical shift values and non-standard NOEs between Na^+ and K^+ conditions (see below).

Comparing the five sequences that adopt the *-ld+l* topology – **342**, **343**, **344**, **442** and **443** – reveals that non-exchangeable proton chemical shifts of thymidines forming the central diagonal loop (T8-T9-T10-T11 in **342**), as well as those of guanosines forming the adjacent G-tetrad (G1-G7-G12-G17 in **342**) are all conserved within 0.1 ppm. This includes several protons

resonating at frequencies well separated from the bulk of similar protons, including: T8H1', T9H1', T10H1', T8H2', T8H3', T9H3' and T11H3'. The NOE pattern of the central loop is closely reproduced between all five systems. In each sample sequential H1'(n)-H8(n+1) NOEs are observed in the G7-T8-T9-T10-T11 region, complemented by a T8Me-G7H8 contact and non-sequential T9H1'-T11H6, T10H1'-G1H1, T10Me-G1H8 cross peaks among others. These non-standard contacts (for a loop region) are also found in NOE lists of the previously solved structures of **342** and **343** suggesting, together with the conserved chemical shift values, that the diagonal loop adopts a similar conformation under Na^+ and K^+ conditions.

Moreover, the spectra of all *-ld+l* G4s present in our dataset all contain a thymidine imino proton resonating around 9.0 ppm, that appear to be protected from solvent exchange (remaining observable up to G4 melting) and gives rise to a single NOE cross-peak of appreciable intensity (to T8H1'). We tentatively assigned this resonance to T10 (third residue of the diagonal loop), based on the following arguments. In Na^+ conditions no imino protons are observed around this frequency, but a strong cross-peak between T8H1' and T10H3 (resonating around 9.8-9.9 ppm in those conditions) is reported. Coincidentally, in the deposited structures of **342** and **343,** T10H3 is positioned within H-bonding distance from T8O2, which could explain its slow solvent exchange.

G4 topology assignment – additional spectral patterns observed for *d+pd* G4s

While **414** produced sharp non-exchangeable proton resonances and was easily assignable using NOESY and ^{31}P-^{1}H COSY spectra, **424** and **434** featured significant line broadening in some regions, later identified as parts of the first and third loops. Fortunately, for G4 topology assignment only the guanosine residues have to be identified unambiguously. In both cases, the two central G-tracts and the central loop were not affected by line broadening and were easily identified by through bond ^{31}P-^{1}H connectivities. The 3' and 5'-terminal G-tracts were in turn distinguished because the 3'-terminal residue lacks the H3'-P connectivity.

No close counterparts of **414**, **424** or **434** were studied previously by NMR. However, for **414** (where all the residues forming the diagonal loops were assigned) a comparison with *-ld+l* G4s from our dataset reveals an intriguing observation. The chemical shift and NOE

pattern characteristic of a four-thymidine diagonal loop in *-ld+l* (described above) is reproduced in the third loop of **414**, but not in the first, even though both are diagonal. Consistently, the unusual thymidine imino proton resonating around 9.0 ppm, assigned to the third residue of the diagonal loop, is observed only for the third loop and not the first. This suggests that the two diagonal loops in **414** adopt distinct conformations. The available structures of *-ld+l* G4s with TTTT loop[2] show that this element forms important stacking interactions with the 5'-guanosine. In **414** the 5'-terminal guanosine belongs to the G-tetrad interacting with the third loop, while the first loop stacks with the G-tetrad containing the 3'-residue. Thus, our observations suggest that the composition of the adjacent tetrad (whether and which terminal residues are present) has a significant impact on the 3D conformation assumed by the TTTT loop.

Supplementary Figure S1_1-64. Main experimental results (UV melting, CD spectroscopy, 1D ^{1}H NMR spectroscopy and gel electrophoresis) for each of the 64 sequences studied (one panel per sequence)

Figure S1_1

1 (**111**): 5' GG**T**GG**T**GG**T**GG 3' 11 nt

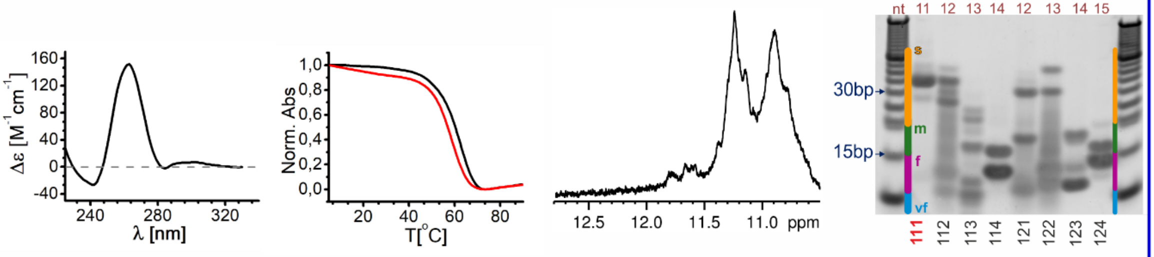

Remarks:
- Broad lines in 1D NMR and slowly migrating bands in PAGE - predominantly G4 aggregates.

Figure S1_2

2 (**112**): 5' GG**T**GG**T**GG**TT**GG 3' 12 nt

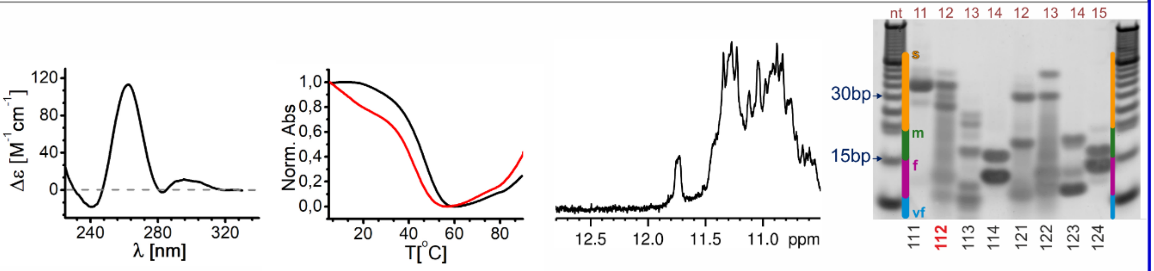

Remarks:
- Broad lines in 1D NMR and slowly migrating bands in PAGE - predominantly G4 aggregates.

Figure S1_3

3 (**113**): 5' GG**T**GG**T**GG**TTT**GG 3' 13 nt

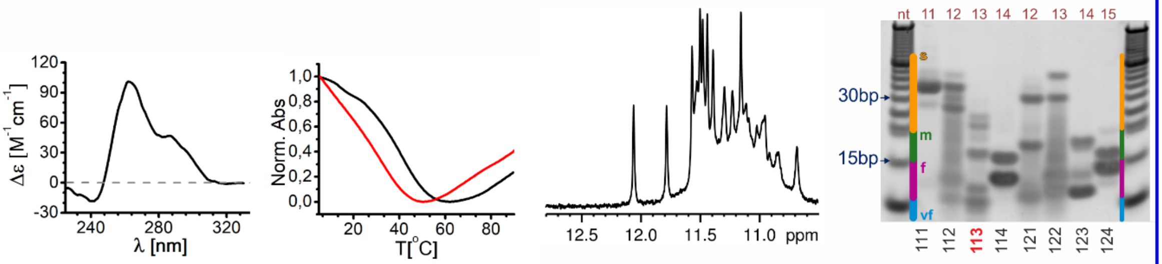

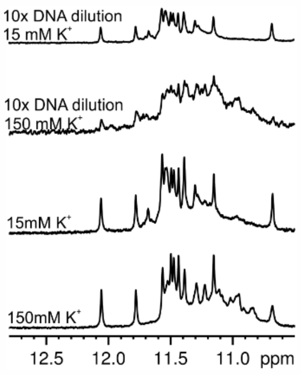

Remarks:

- NMR conditions - mixture of multimolecular forms, including aggregates.
- PAGE conditions – slow, medium and fast migrating bands are present.
- NMR with dilution of DNA and K^+ - elimination of G4 aggregates.

Figure S1_4

4 (**114**): 5' GG**T**GG**T**GG**TTTT**GG 3' 14nt

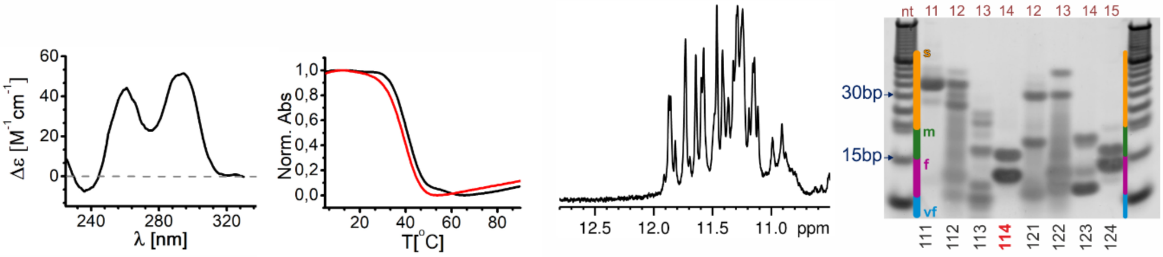

Remarks:

- NMR conditions - mixture of multimolecular forms.
- PAGE conditions - one medium and one fast migrating band.

Figure S1_5

5 (**121**): 5' GG**T**GG**TT**GG**T**GG 3' 12 nt

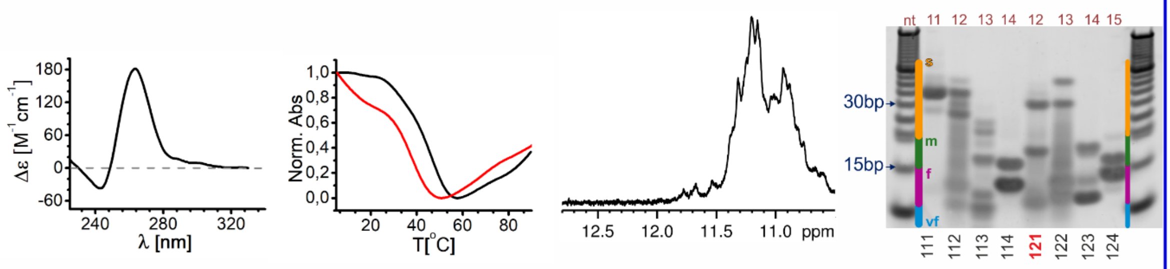

Remarks:
- NMR conditions - predominantly G4 aggregates.

- PAGE conditions - some faster migrating bands indicate that other G4 structures may be present.

Figure S1_6

6 (**122**): 5' GG**T**GG**TT**GG**TT**GG 3' 13 nt

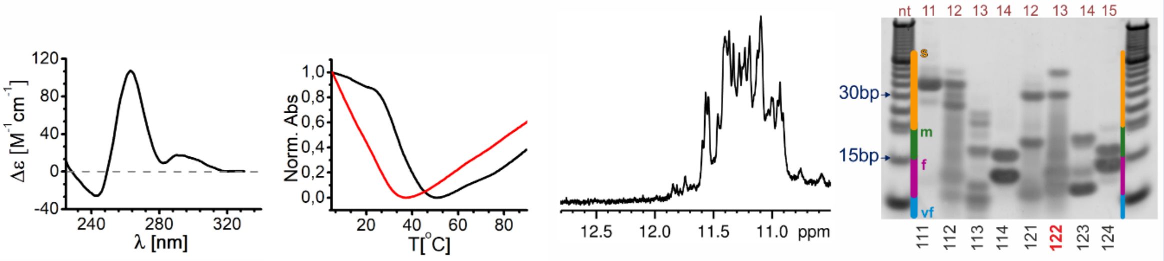

Remarks:
- NMR conditions - mixture of multimolecular forms, including aggregates.

- PAGE conditions - slowly migrating bands, consistent with NMR observations.

Figure S1_7

7 (**123**): 5' GG**T**GG**TT**GG**TTT**GG 3' — 14 nt

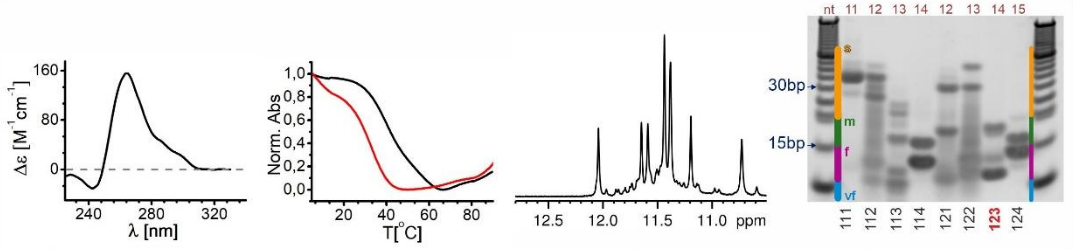

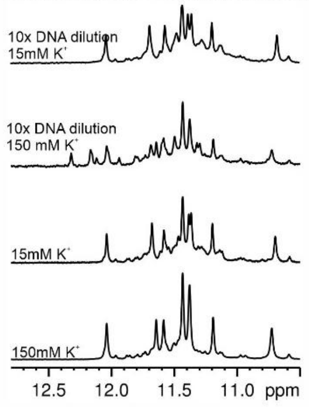

Remarks:
- NMR conditions - a single, multimolecular form dominates.

- PAGE conditions - the presence of one medium and one fast migrating band.

- NMR with dilution of DNA - a population of a unimolecular form is induced.

Figure S1_8

8 (**124**): 5' GG**T**GG**TT**GG**TTTT**GG 3' — 15nt

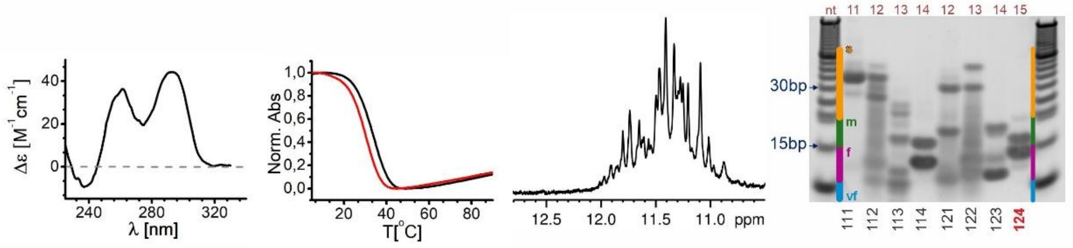

Remarks:
- NMR conditions - mixture of multimolecular forms.

- PAGE conditions - one medium and one fast migrating band.

Figure S1_9

9 (**131**): 5' GG**T**GG**TTT**GG**T**GG 3' 13 nt

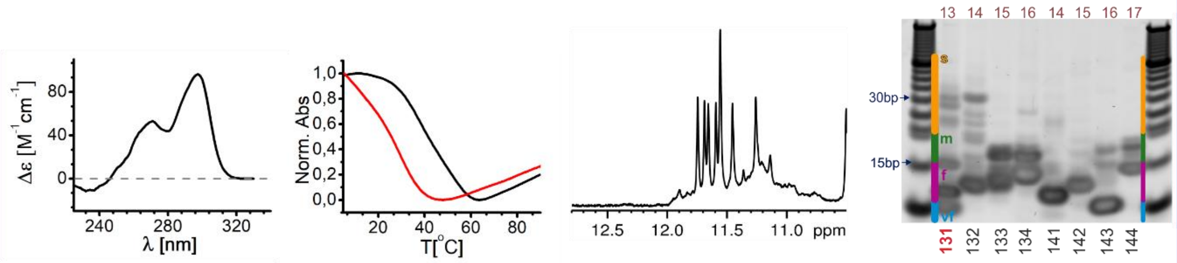

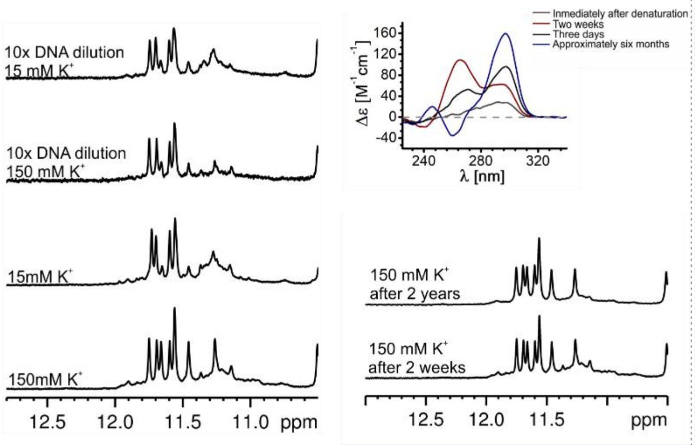

Remarks:
- NMR conditions - one well-resolved multimolecular form and a G4 aggregate.
- PAGE conditions - both slow and fast migrating bands are present. The latter likely correspond to unimolecular forms impossible to observe in NMR experiments.
- NMR with dilution of DNA and/or K^+ - no significant changes observed. The discrepancy between NMR and PAGE observations remains.
- Other observations - thermodynamic equilibrium takes a long time to establish itself (see CD spectra series in time), the aggregate slowly disappears in time

Figure S1_10

10 (**132**): 5' GG**T**GG**TTT**GG**TT**GG 3' 14 nt

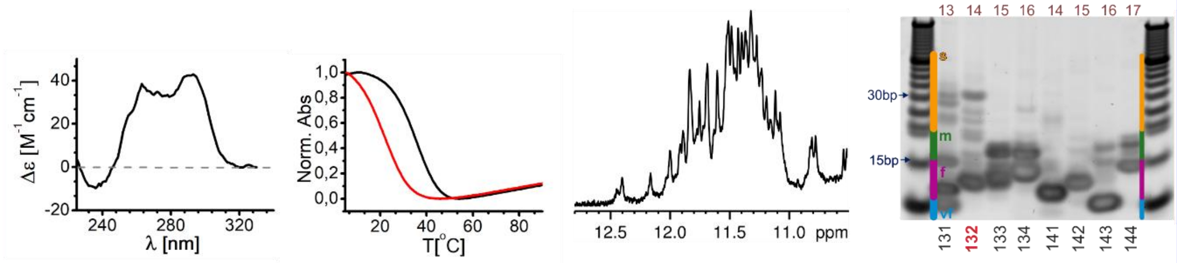

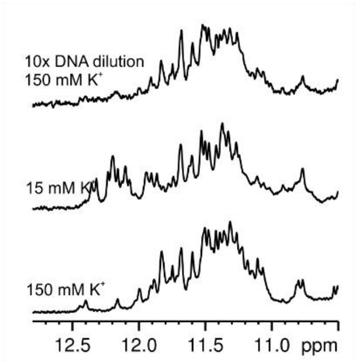

Remarks:
- NMR conditions - multimolecular forms dominate.
- PAGE conditions - both slow and fast migrating bands are present.
- NMR with dilution of K^+ - an increased population of a unimolecular form.

Figure S1_11

11 (**133**): 5' GG**T**GG**TTT**GG**TTT**GG 3' 15nt

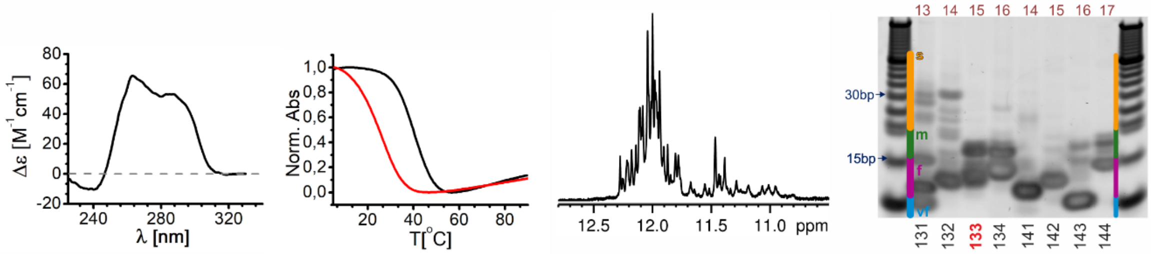

Remarks:

- NMR conditions - mixture of multimolecular forms.

- PAGE conditions - one medium and one fast migrating band

Figure S1_12

12 (**134**): 5' GG**T**GG**TTT**GG**TTTT**GG 3' 16 nt

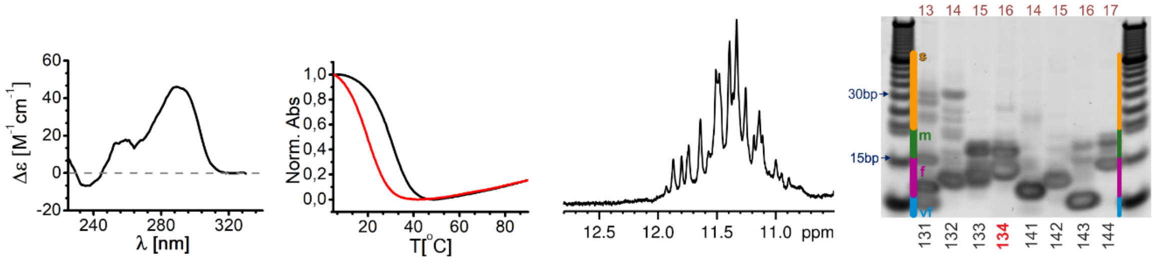

Remarks:

- NMR conditions - mixture of multimolecular forms.

- PAGE conditions - one medium and one fast migrating band.

Figure S1_13

13 (**141**): 5' GG**T**GG**TTTT**GG**T**GG 3' 14 nt

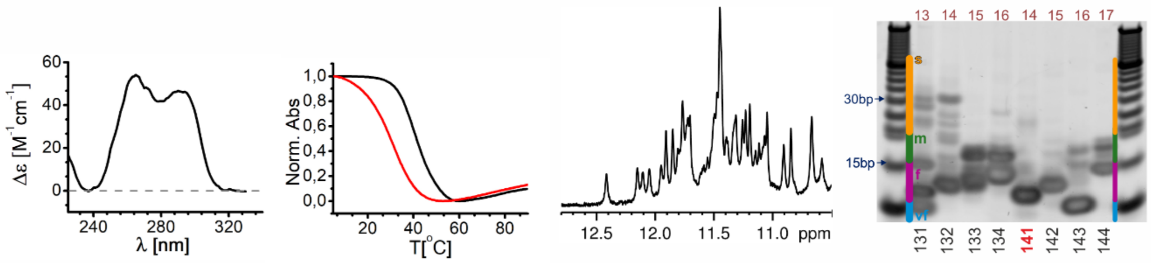

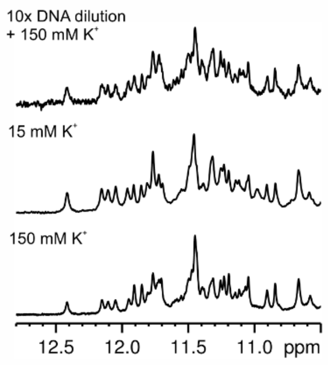

Remarks:

- NMR conditions - mixture of multi- and unimolecular forms.

- PAGE conditions - one fast migrating band.

- NMR with dilution of DNA and/or K^+ - no significant changes observed. The discrepancy between NMR and PAGE observations remains.

Figure S1_14

14 (**142**): 5' GGTGGTTTTGGTTGG 3' — 15 nt

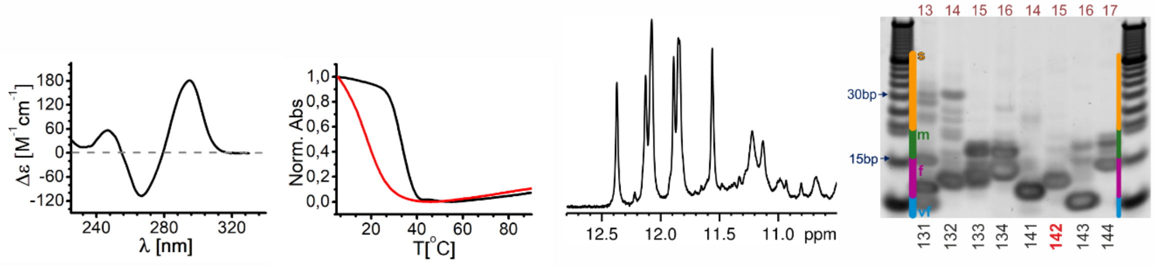

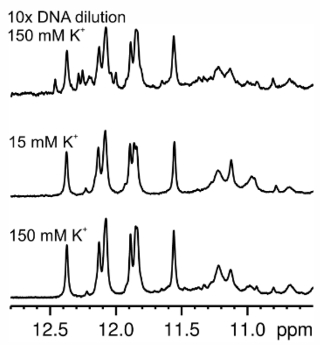

Remarks:

- NMR conditions - a single unimolecular form dominates.

- NMR with dilution of DNA and/or K^+ - no significant changes observed.

Figure S1_15

15 (**143**): 5' GGTGGTTTTGGTTTGG 3' — 16 nt

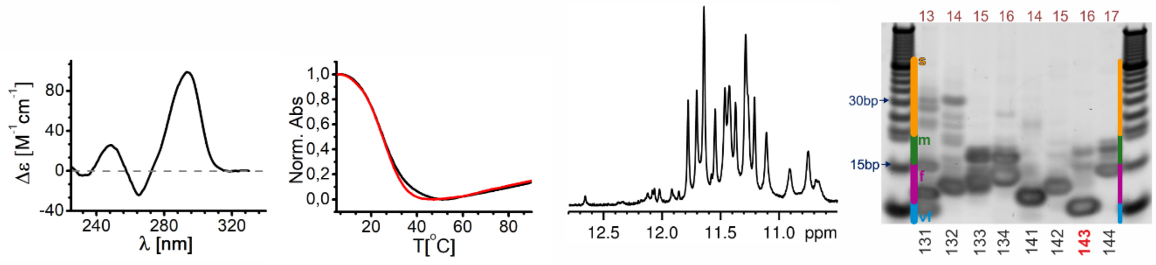

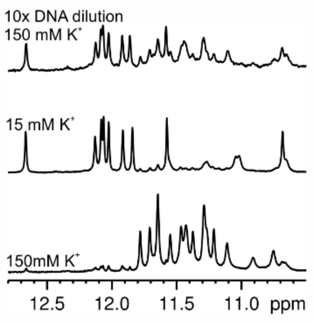

Remarks:

- NMR conditions - multimolecular forms dominate.

-PAGE conditions - one fast migrating band.

-NMR with dilution of DNA - an increased population of a unimolecular form.

-NMR with dilution of K^+ - a single unimolecular form.

-UV melting - particularly low T_m, preventing NMR structure determination.

Figure S1_16

16 (**144**): 5' GG**T**GG**TTTT**GG**TTTT**GG 3' — 17 nt

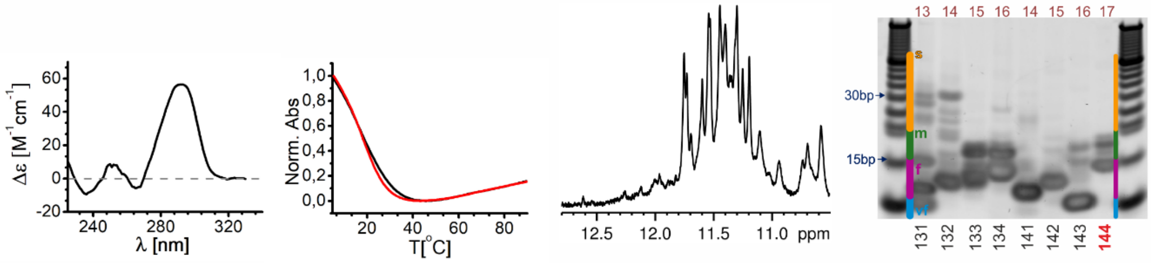

Remarks:
- NMR conditions - multimolecular forms dominate.

- UV melting - the lowest T_m in the set.

Figure S1_17

17 (**211**): 5' GG**TT**GG**T**GG**T**GG 3' — 12 nt

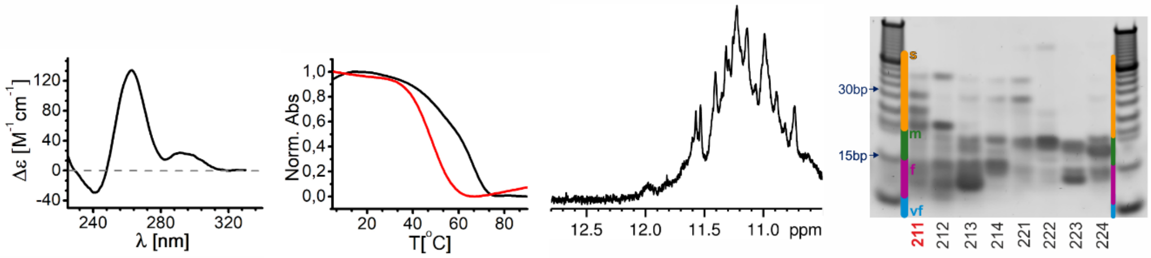

Remarks:
- NMR conditions - multimolecular forms dominate, including aggregates.

Figure S1_18

18 (**212**): 5' GG**TT**GG**T**GG**TT**GG — 13 nt

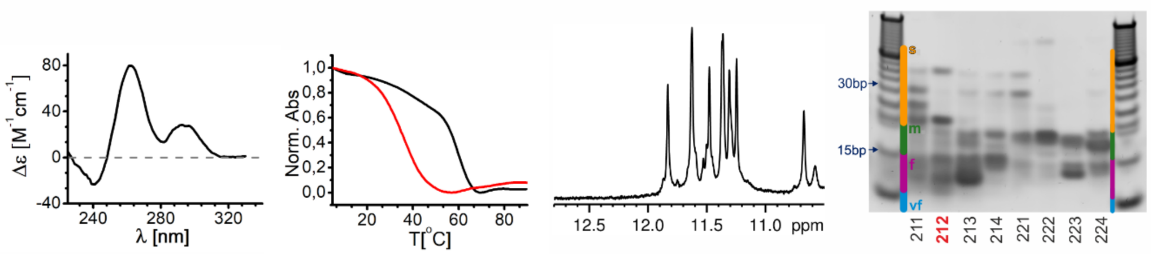

Remarks:
- NMR conditions - one well-resolved multimolecular form.

- PAGE conditions - multiple bands.

Figure S1_19

19 (**213**): 5' GG**TT**GG**T**GG**TTT**GG 3' 14 nt

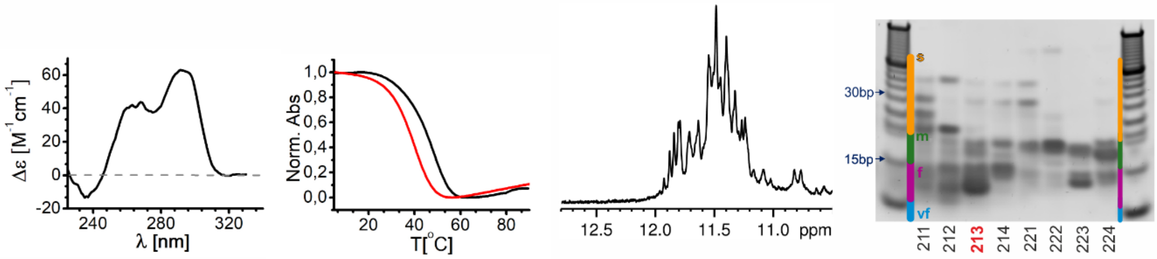

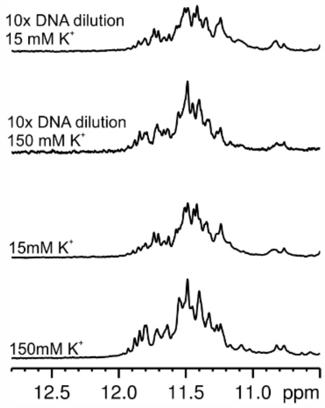

Remarks:
- NMR conditions - multimolecular forms dominate, including aggregates.

- PAGE conditions – fast migrating bands present.

- NMR with dilution of DNA and/or K^+ - no significant changes observed. The discrepancy between NMR and PAGE observations remains.

Figure S1_20

20 (**214**): 5' GG**TT**GG**T**GG**TTTT**GG 3' 15nt

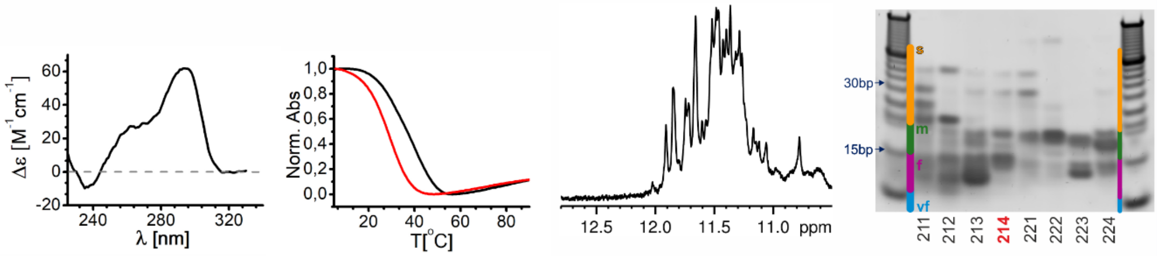

Remarks:
- NMR conditions - multimolecular forms dominate, including aggregates.

- PAGE conditions - one medium and one fast migrating band.

Figure S1_21

21 (**221**): 5' GG**TT**GG**TT**GG**T**GG 3' 13 nt

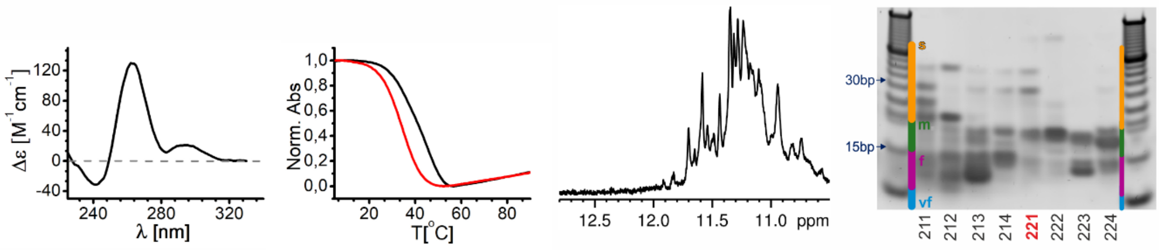

Remarks:
- NMR conditions - multimolecular forms dominate.

Figure S1_22

22 (**222**): 5' GG**TT**GG**TT**GG**TT**GG 3' 14 nt

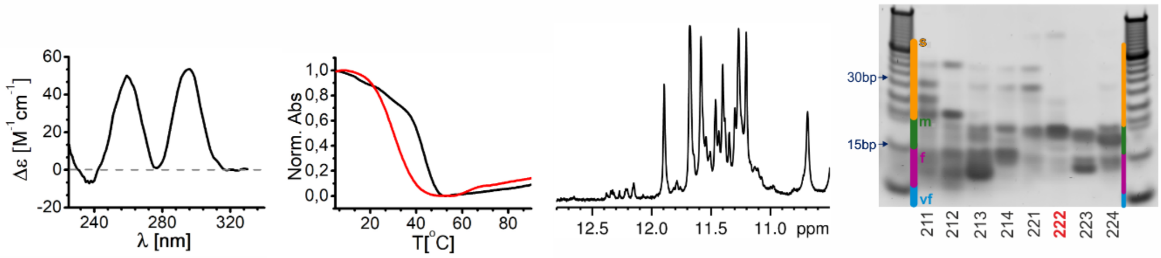

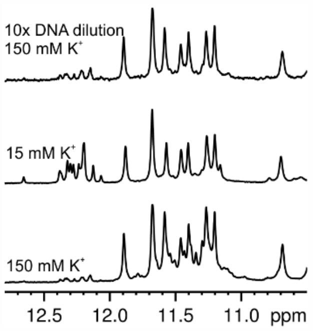

Remarks:
- NMR conditions - a single multimolecular form dominate.

- NMR with dilution of K^+ - an increased population of a unimolecular form is induced

Figure S1_23

23 (**223**): 5' GG**TT**GG**TT**GG**TTT**GG 3' 15 nt

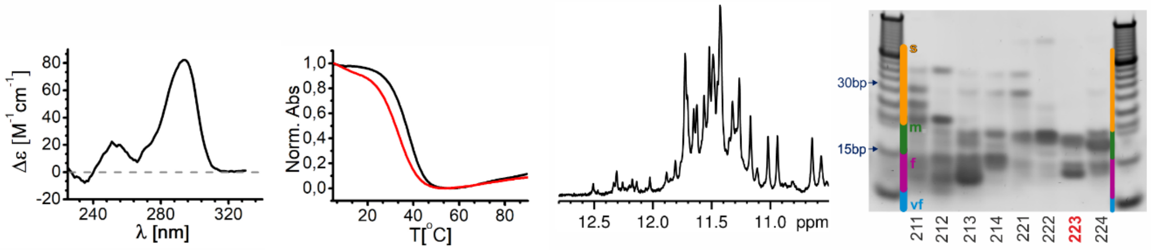

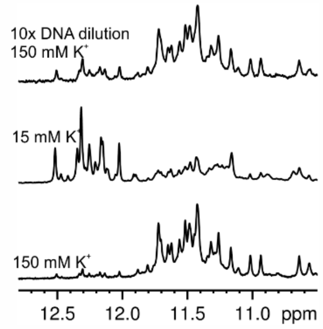

Remarks:
- NMR conditions - multimolecular forms dominate.
- PAGE conditions - one medium and one fast migrating band.
- NMR with dilution of K^+ - a dominant population of a unimolecular form is induced.

Figure S1_24

23 (**224**): 5' GG**TT**GG**TT**GG**TTTT**GG 3' 16 nt

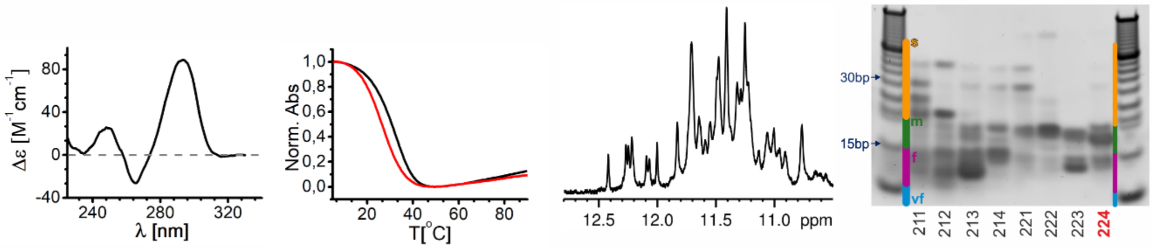

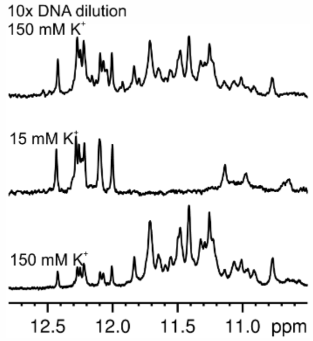

Remarks:
- NMR conditions - multimolecular forms dominate.
- PAGE conditions - a fast migrating band in present.
- NMR with dilution of DNA - mixture of uni- and multimolecular forms.
- NMR with dilution of K^+ - a single unimolecular form.

Figure S1_25

25 (**231**): 5' GG**TT**GG**TTT**GG**T**GG 3' 14 nt

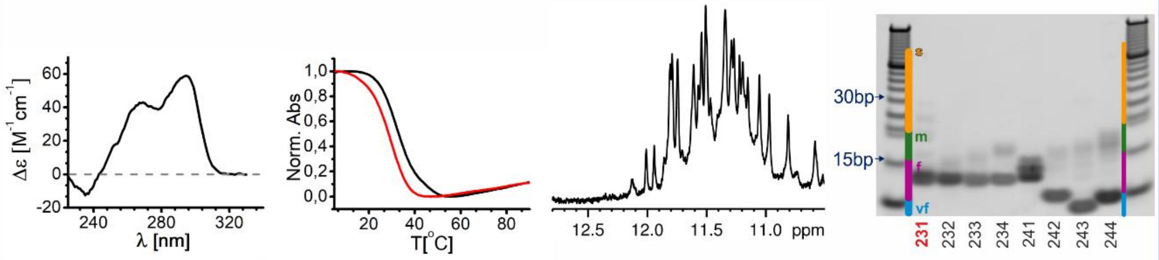

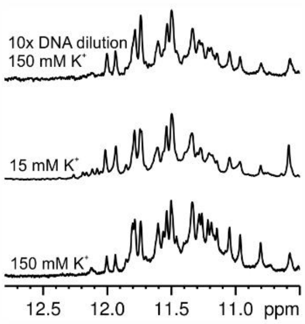

Remarks:
- NMR conditions - multimolecular forms dominate.

- PAGE conditions - a single fast migrating band.

- NMR with dilution of DNA and/or K^+ - no significant changes observed. The discrepancy between NMR and PAGE observations remains.

Figure S1_26

26 (**232**): 5' GG**TT**GG**TTT**GG**TT**GG 3' 15nt

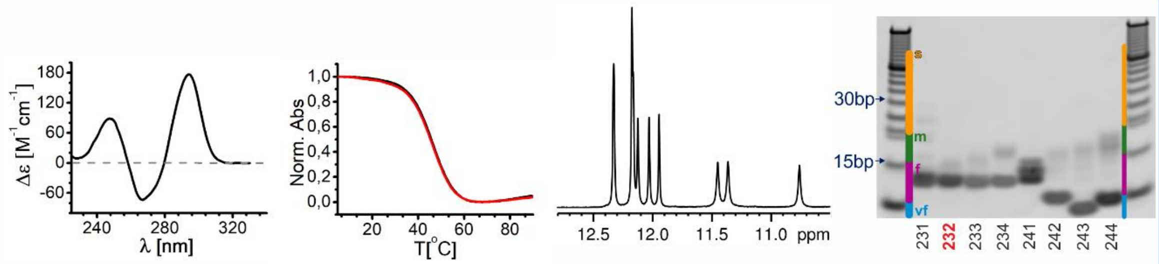

Remarks:
- NMR conditions - a single unimolecular form (+l+l+l topology).

Figure S1_27

27 (**233**): 5' GG**TT**GG**TTT**GG**TTT**GG 3' 16 nt

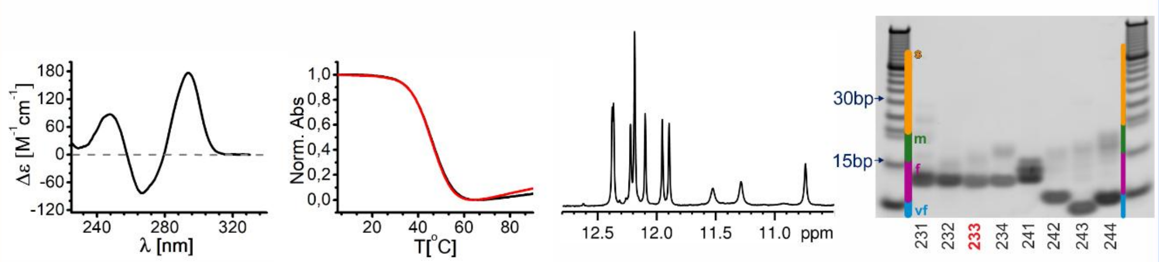

Remarks:
- NMR conditions - a single unimolecular form (+l+l+l topology).

Figure S1_28

28 (**234**): 5' GG**TT**GG**TTT**GG**TTTT**GG 3' — 17 nt

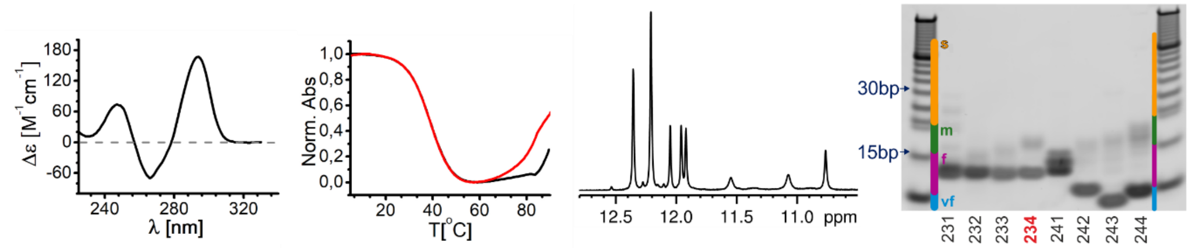

Remarks:
- NMR conditions - a single unimolecular form (+l+l+l topology).

Figure S1_29

29 (**241**): 5' GG**TT**GG**TTTT**GG**T**GG 3' — 15 nt

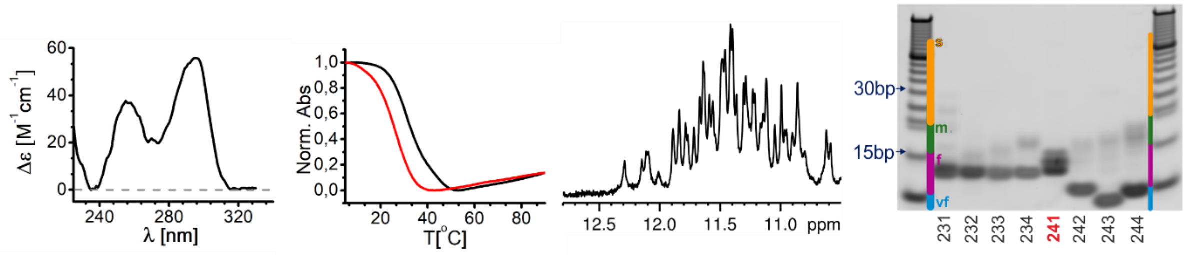

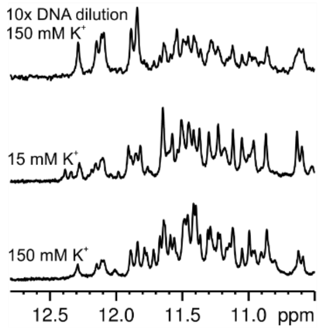

Remarks:
- NMR conditions - multimolecular forms dominate.

- PAGE conditions - fast migrating bands are present.

- NMR with dilution of DNA - an increased population of a unimolecular form.

Figure S1_30

30 (**242**): 5' GG**TT**GG**TTTT**GG**TT**GG 3' 16 nt

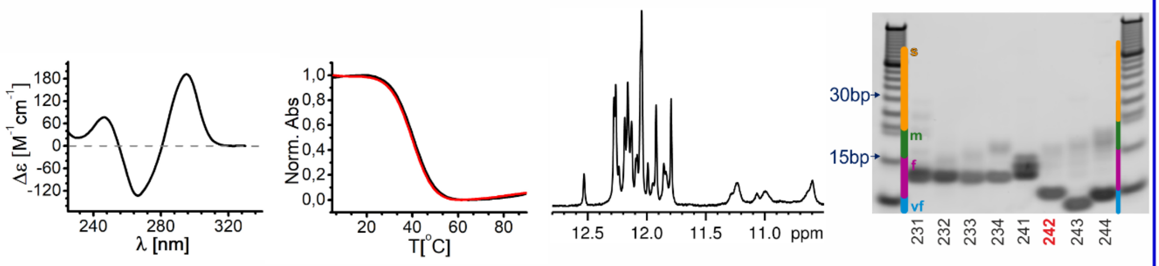

Remarks:

- NMR conditions – a mixture of unimolecular forms.

- PAGE conditions - one fast migrating band.

Figure S1_31

31 (**243**): 5' GG**TT**GG**TTTT**GG**TTT**GG 3' 17nt

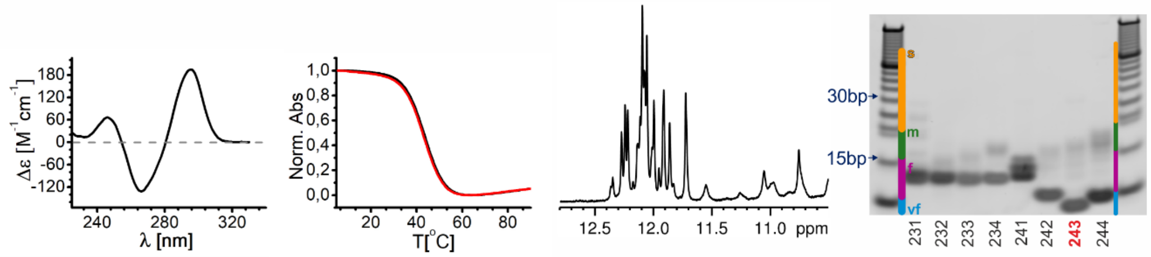

Remarks:

- NMR conditions – a mixture of unimolecular forms.

- PAGE conditions - one very fast migrating band.

Figure S1_32

32 (**244**): 5' GG**TT**GG**TTTT**GG**TTTT**GG 3' 18 nt

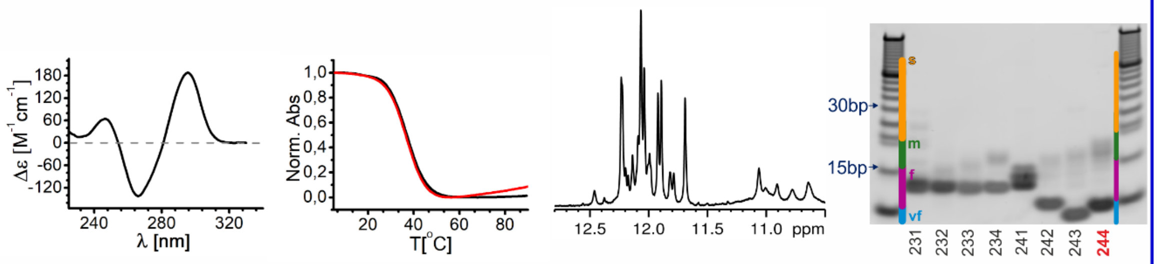

Remarks:

- NMR conditions – a mixture of unimolecular forms.

- PAGE conditions - one fast migrating band.

Figure S1_33

33 (**311**): 5' GG**TTT**GG**T**GG**T**GG 3' — 13 nt

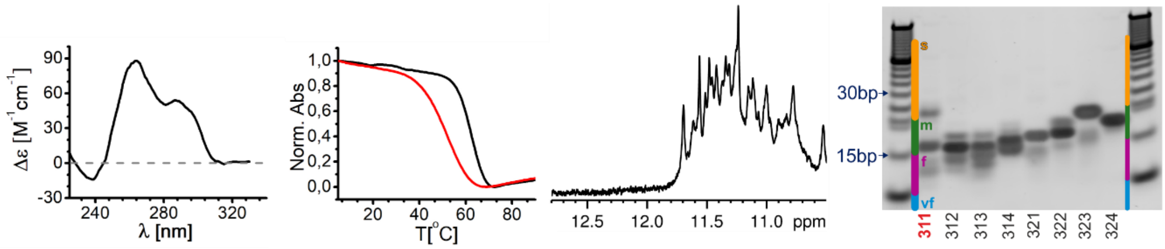

Remarks:
- NMR conditions - multimolecular forms dominate, including aggregates.

- PAGE conditions - one medium and one slow migrating band.

Figure S1_34

34 (**312**): 5' GG**TTT**GG**T**GG**TT**GG 3' — 14 nt

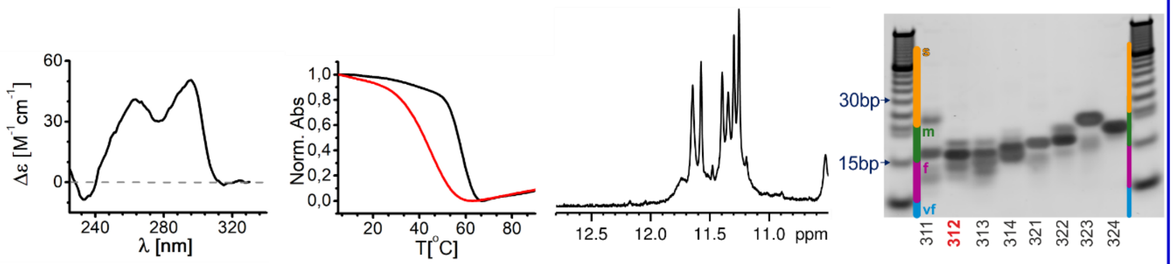

Remarks:
- NMR conditions - a single multimolecular form dominates.

Figure S1_35

35 (**313**): 5' GG**TTT**GG**T**GG**TTT**GG 3' — 15nt

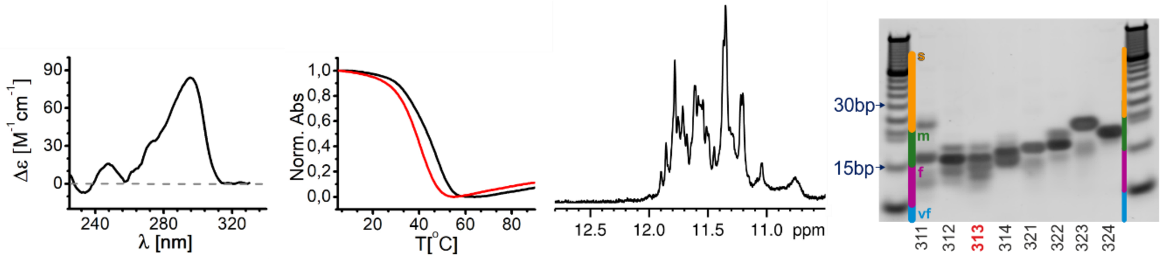

Remarks:
- NMR conditions - multimolecular forms dominate.

- PAGE conditions - a single medium migrating band.

Figure S1_36

36 (**314**): 5' GG**TTT**GGTGG**TTTT**GG 3' 16 nt

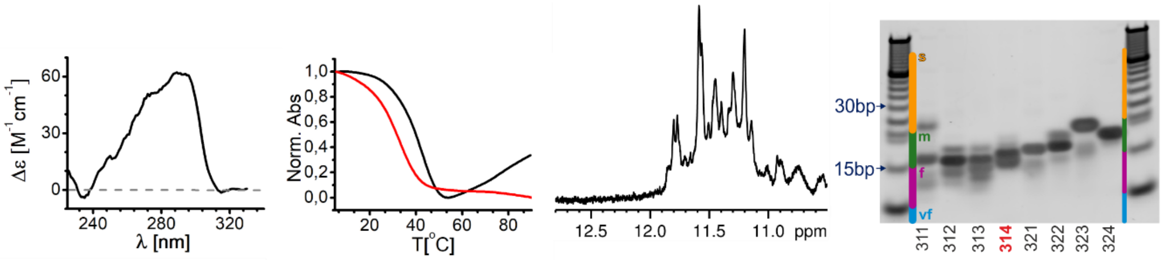

Remarks:

- NMR conditions - multimolecular forms dominate.

Figure S1_37

37 (**321**): 5' GG**TTT**GG**TT**GG**T**GG 3' 14 nt

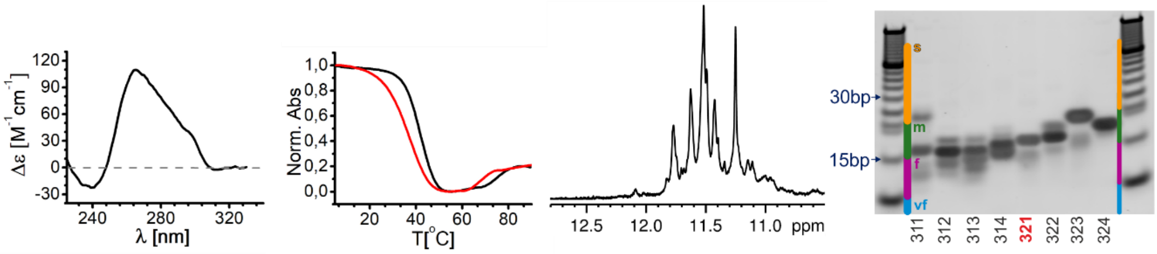

Remarks:

- NMR conditions - a single multimolecular form dominates.

Figure S1_38

38 (**322**): 5' GG**TTT**GG**TT**GG**TT**GG 3' 15 nt

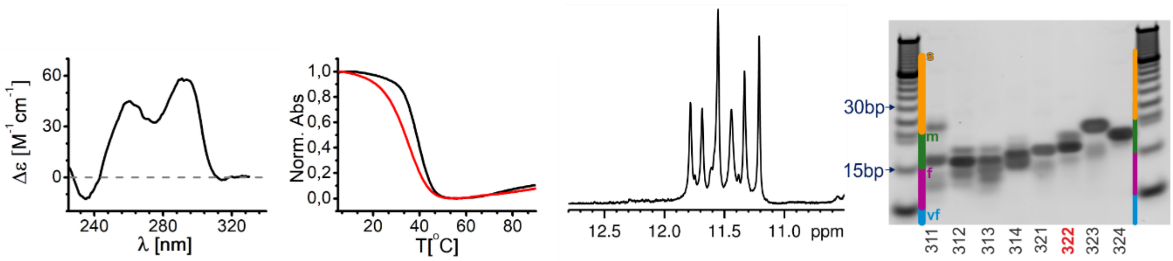

Remarks:
- NMR conditions - a single multimolecular form dominates.

Figure S1_39

39 (**323**): 5' GG**TTT**GG**TT**GG**TTT**GG 3' 16 nt

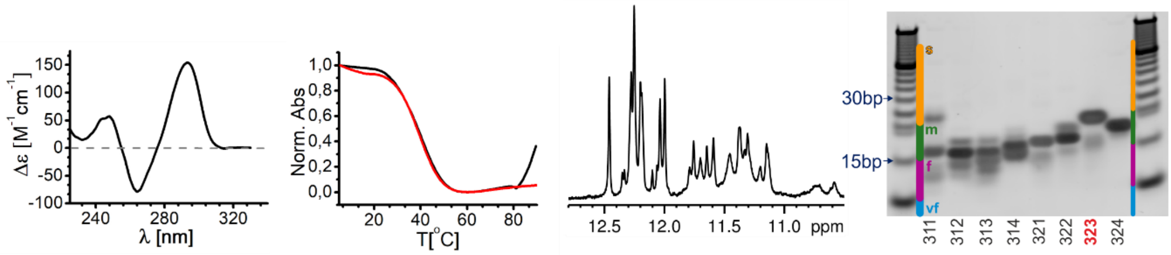

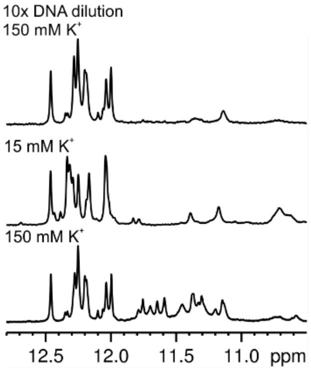

Remarks:
- NMR conditions - mixture of multi- and unimolecular forms (-l-l-l topology).
- PAGE conditions - one medium migrating band.
- NMR with dilution of DNA and/or K^+ - a single unimolecular form.

Figure S1_40

40 (**324**): 5' GG**TTT**GG**TT**GG**TTTT**GG 3' 17 nt

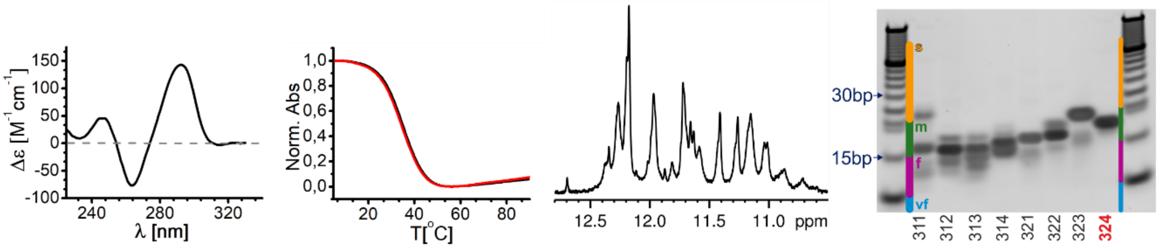

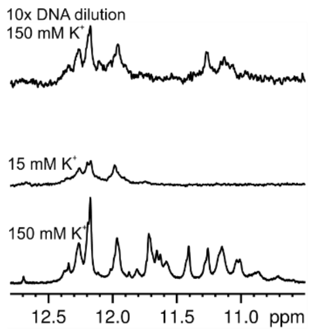

Remarks:

- NMR conditions - mixture of multi- and unimolecular form (presumably -l-l-l topology).
- PAGE conditions - one medium migrating band.
- NMR with dilution of DNA and/or K^+ - a single unimolecular form.

Figure S1_41

41 (**331**): 5' GG**TTT**GG**TTT**GG**T**GG 3' 15 nt

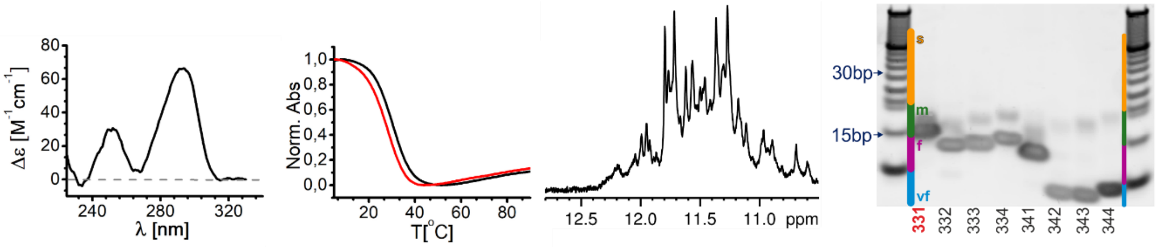

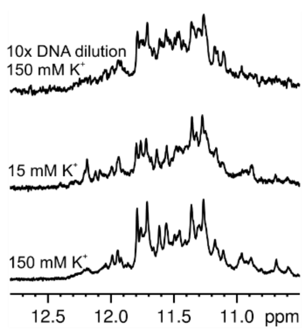

Remarks:

- NMR conditions - multimolecular forms dominate.
- PAGE conditions - one medium migrating band.
- NMR with dilution of DNA and/or K^+ - no significant changes observed. The discrepancy between NMR and PAGE observations remains.

Figure S1_42

42 (**332**): 5' GG**TTT**GG**TTT**GG**TT**GG 3' 16nt

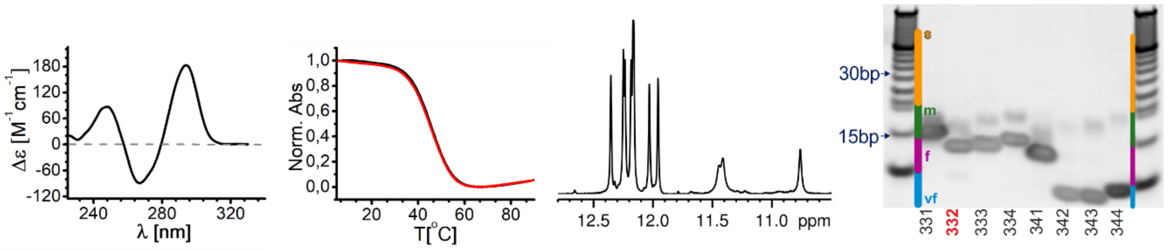

Remarks:
- NMR conditions - a single unimolecular form (+I+I+I topology).

Figure S1_43

43 (**333**): 5' GG**TTT**GG**TTT**GG**TTT**GG 3' 17 nt

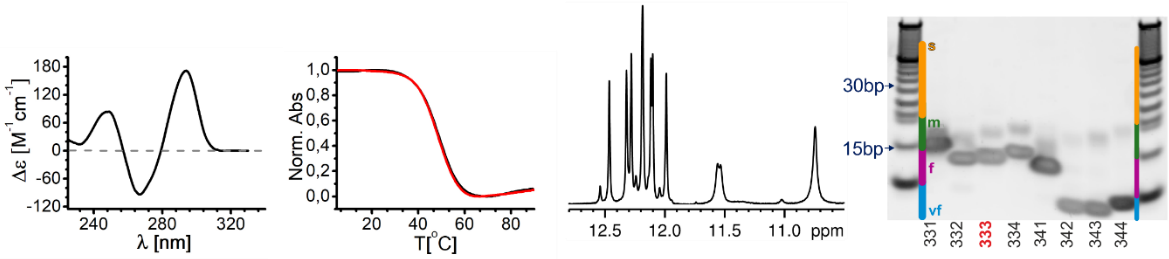

Remarks:
- NMR conditions - a single unimolecular form (+I+I+I topology).

Figure S1_44

44 (**334**): 5' GG**TTT**GG**TTT**GG**TTTT**GG 3' 18nt

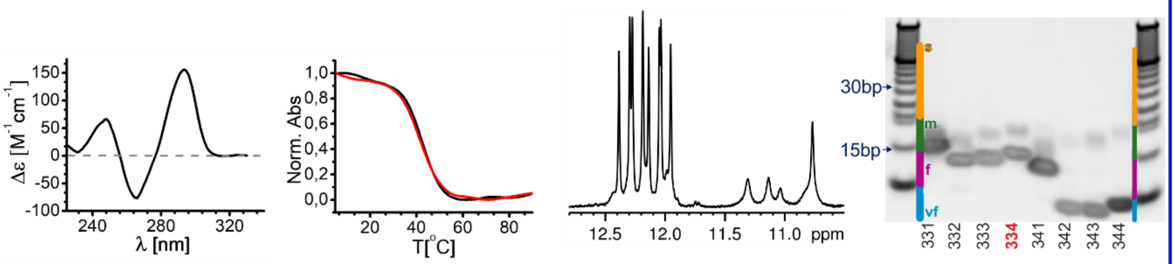

Remarks:
- NMR conditions - a single unimolecular form (+I+I+I topology).

Figure S1_45

45 (**341**): 5' GG**TTT**GG**TTTT**GGTGG 3' 16 nt

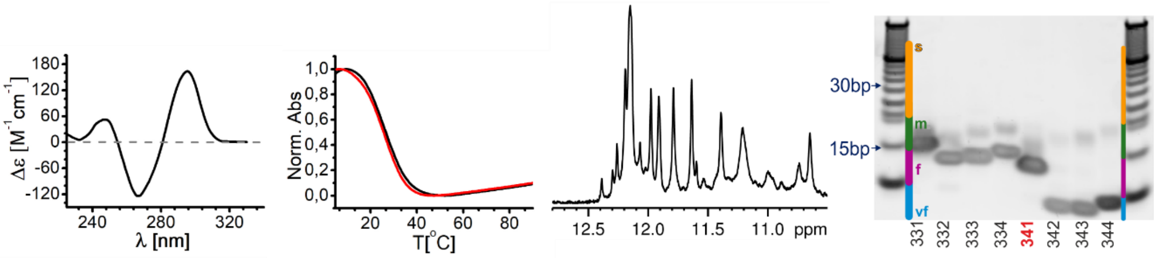

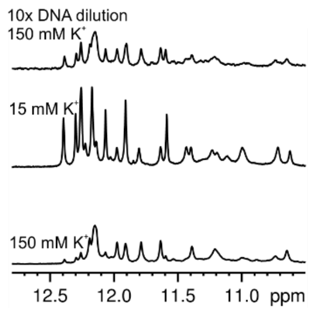

Remarks:

- NMR conditions - a single multimolecular form dominates.
- PAGE conditions - one fast migrating band.
- NMR with dilution of K^+ - a different unimolecular form is induced.
- UV melting - particularly low T_m, preventing NMR structure determination.

Figure S1_46

46 (**342**): 5' GG**TTT**GG**TTTT**GG**TT**GG 3' 17 nt

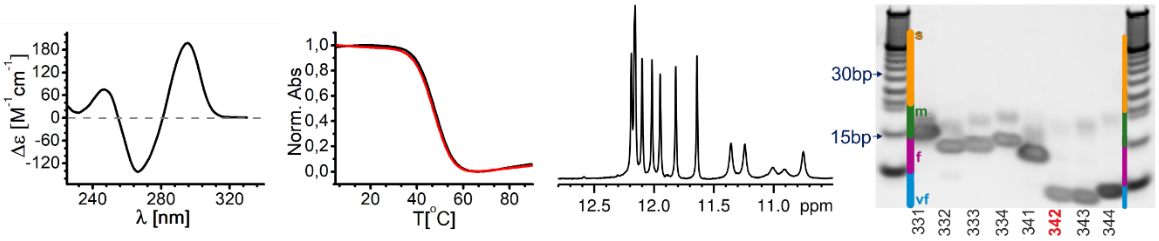

Remarks:

- NMR conditions - a single unimolecular form (-ld+l topology).

Figure S1_47

47 (**343**): 5' GG**TTT**GG**TTTT**GG**TTT**GG 3' 18nt

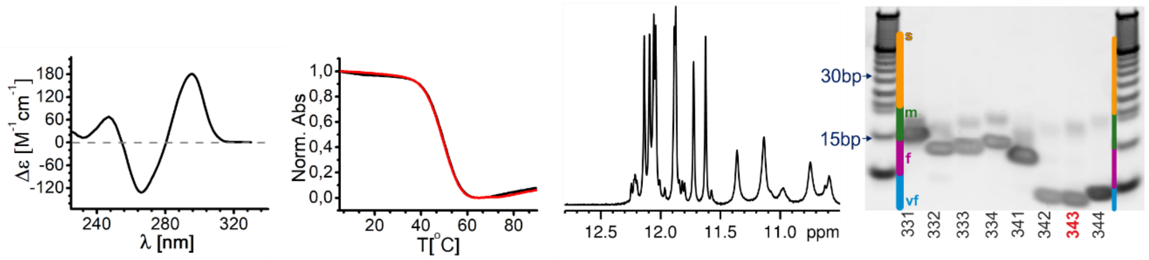

Remarks:

- NMR conditions - a single unimolecular form (-ld+l topology) dominates.

Figure S1_48

48 (**344**): 5' GG**TTT**GG**TTTT**GG**TTTT**GG 3' 19 nt

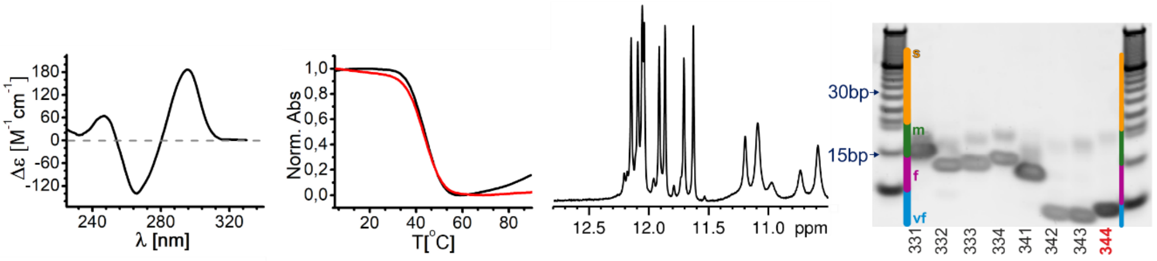

Remarks:
- NMR conditions - a single unimolecular form (-ld+l topology) dominates.

Figure S1_49

49 (**411**): 5' GG**TTTT**GG**T**GG**T**GG 3' 14 nt

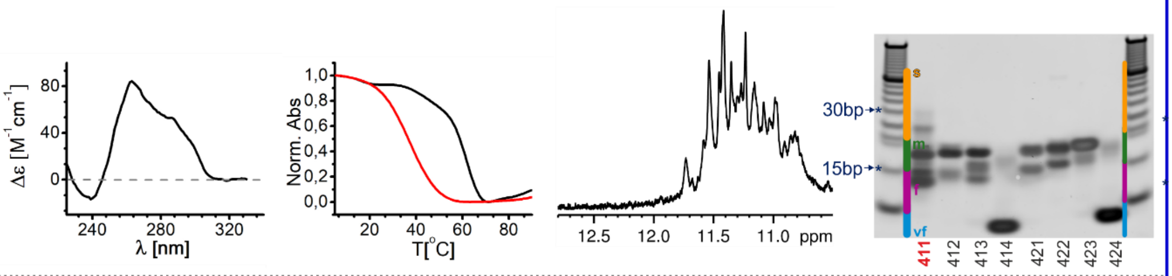

Remarks:
- NMR conditions - multimolecular forms dominate, including aggregates.

- PAGE conditions - one medium and one fast migrating band.

Figure S1_50

50 (**412**): 5' GG**TTTT**GG**T**GG**TT**GG 3' 15 nt

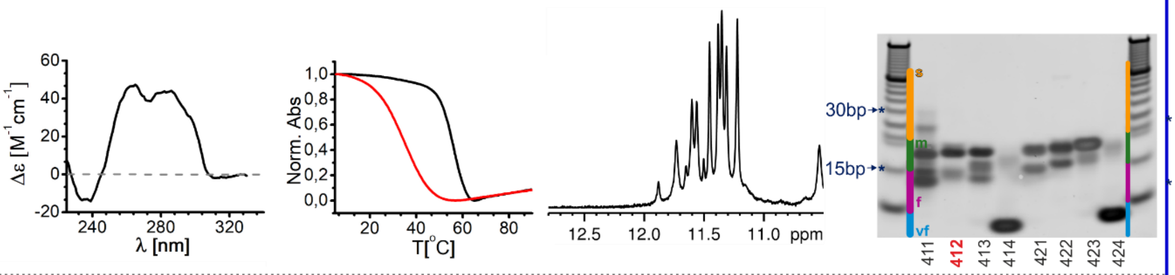

Remarks:
- NMR conditions - a single multimolecular form dominates.

- PAGE conditions - one medium and one slow migrating band.

Figure S1_51

51 (**413**): 5' GG**TTTT**GGTGG**TTT**GG 3' 16nt

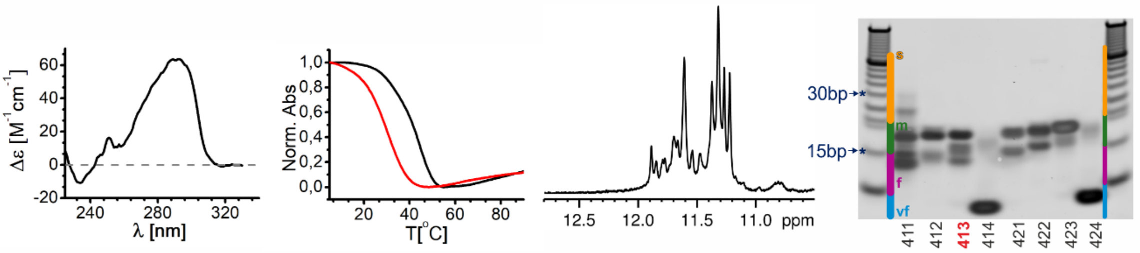

Remarks:
- NMR conditions - a single multimolecular form dominates.

Figure S1_52

52 (**414**): 5' GG**TTTT**GGTGG**TTTT**GG 3' 17 nt

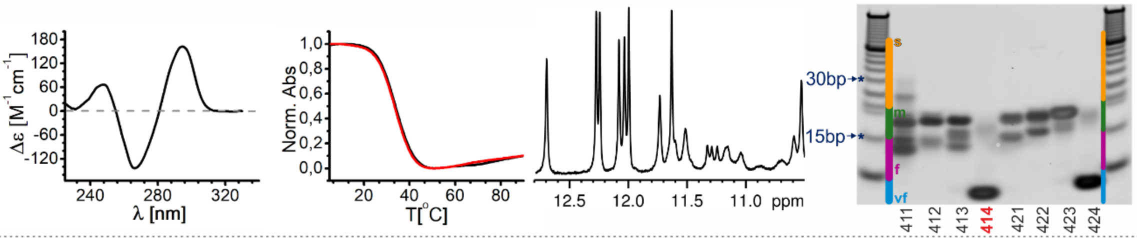

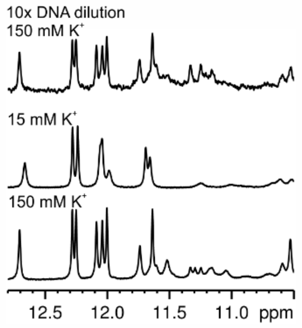

Remarks:
- NMR conditions - a single multimolecular form (d+pd topology) dominates.

- PAGE conditions - one very fast migrating band.

- NMR with dilution of K^+ - a single unimolecular form.

Figure S1_53

53 (**421**): 5' GG**TTTT**GG**TT**GG**T**GG 3' 15 nt

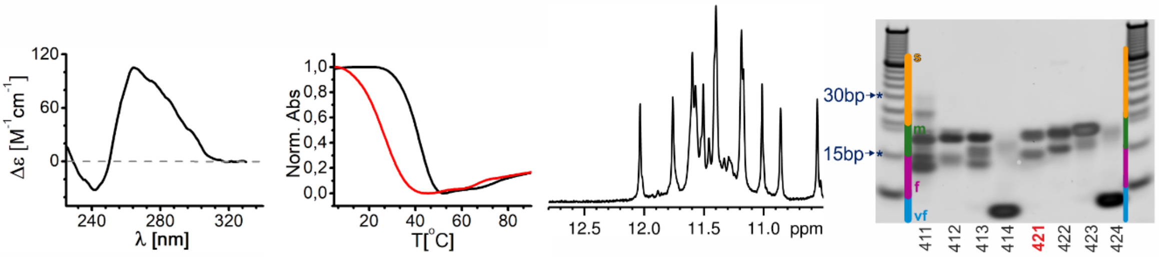

Remarks:
- NMR conditions – one or two multimolecular forms dominate.

Figure S1_54

54 (**422**): 5' GG**TTTT**GG**TT**GG**TT**GG 3' 16nt

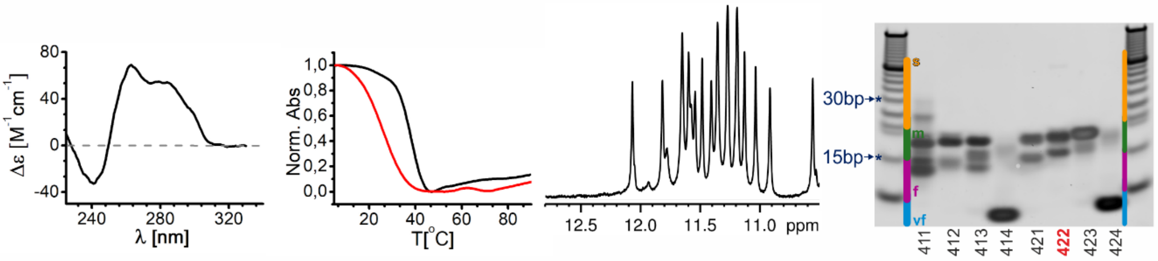

Remarks:
- NMR conditions – two multimolecular forms dominate.

Figure S1_55

55 (**423**): 5' GG**TTTT**GG**TT**GG**TTT**GG 3' 17 nt

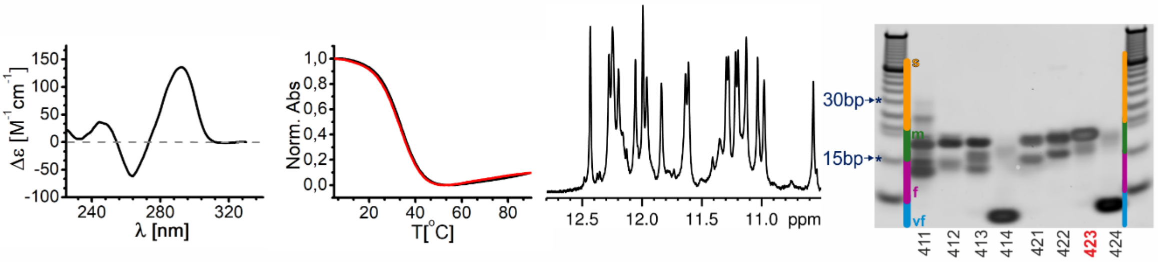

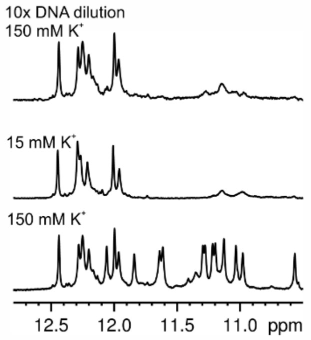

Remarks:
- NMR conditions - mixture of multi- and unimolecular form (presumably -l-l-l topology).
- PAGE conditions - one medium migrating band.
- NMR with dilution of DNA and/or K^+ - a single unimolecular form.

Figure S1_56

56 (**424**): 5' GG**TTTT**GG**TT**GG**TTTT**GG 3' 18 nt

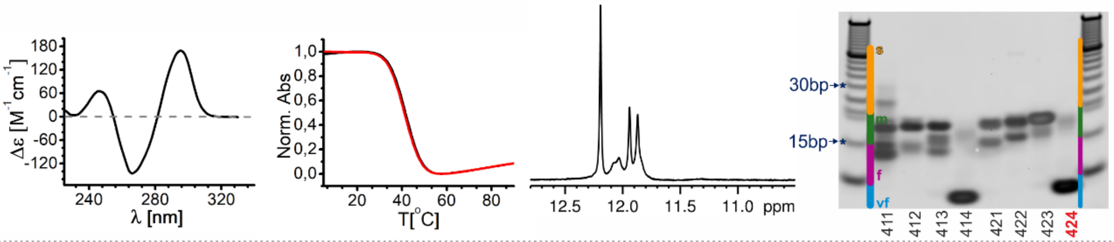

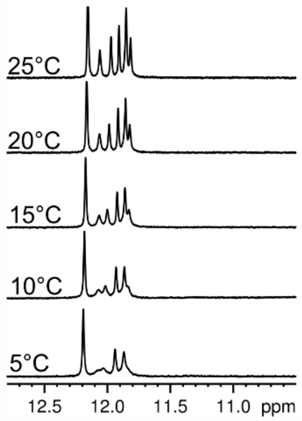

Remarks:
- NMR conditions - a single unimolecular form (d+pd topology).
- PAGE conditions - one very fast migrating band.
- At lower temperatures many NMR resonances broadened by exchange.

Figure S1_57

57 (**431**): 5' GG**TTTT**GG**TTT**GG**T**GG 3' 16nt

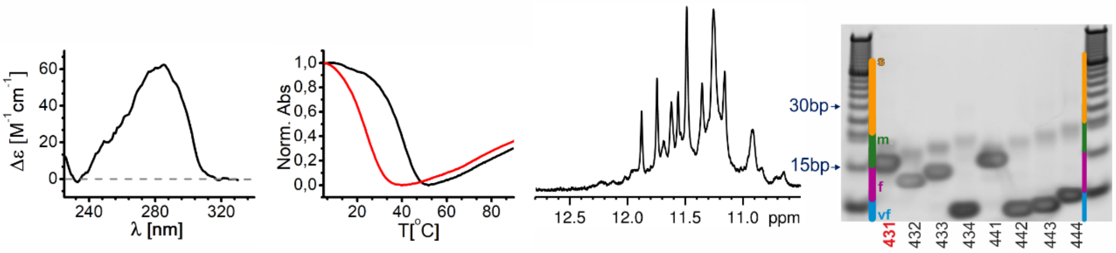

Remarks:
- NMR conditions - multimolecular forms dominate.
- PAGE conditions - one medium migrating band.

Figure S1_58

58 (**432**): 5' GG**TTTT**GG**TTT**GG**TT**GG 3' 17 nt

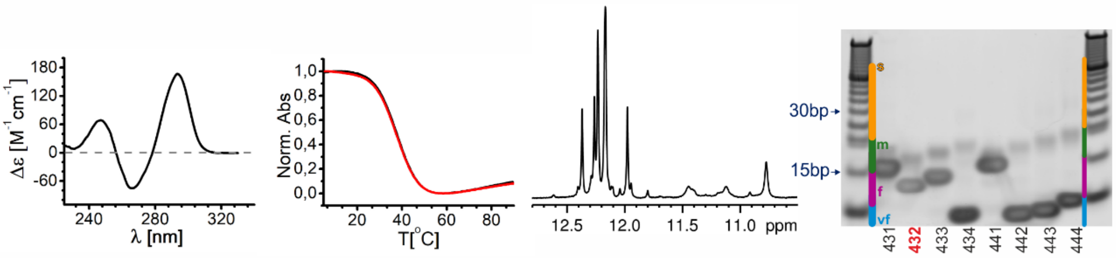

Remarks:
- NMR conditions - a single unimolecular form (+l+l+l topology).

Figure S1_59

59 (**433**): 5' GG**TTTT**GG**TTT**GG**TTT**GG 3' 18nt

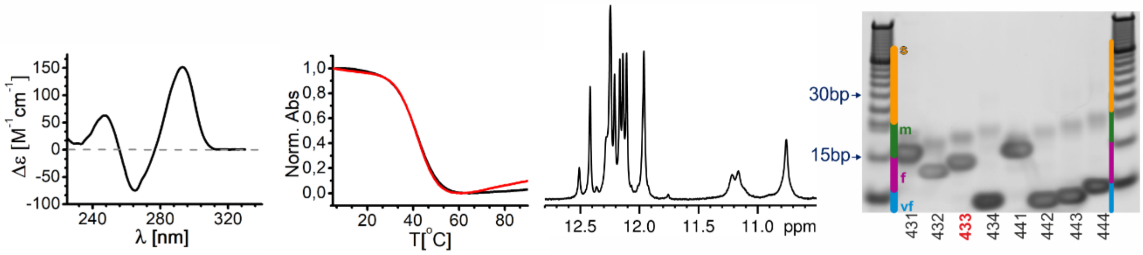

Remarks:
- NMR conditions - a single unimolecular form (+l+l+l topology).

Figure S1_60

60 (**434**): 5' GG**TTTT**GG**TTT**GG**TTTT**GG 3' 19 nt

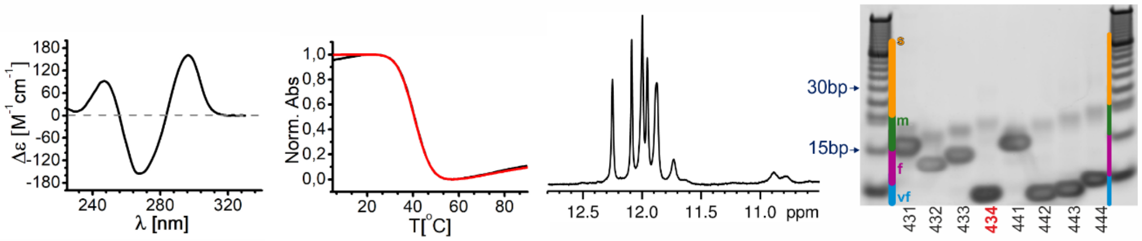

Remarks:
- NMR conditions - a single unimolecular form (d+pd topology).

- At lower temperatures many NMR resonances broadened by exchange.

Figure S1_61

61 (**441**): 5' GG**TTTT**GG**TTTT**GG**T**GG 3' 17 nt

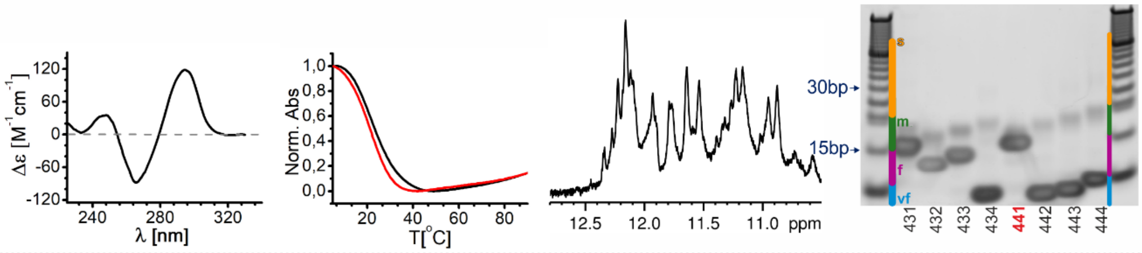

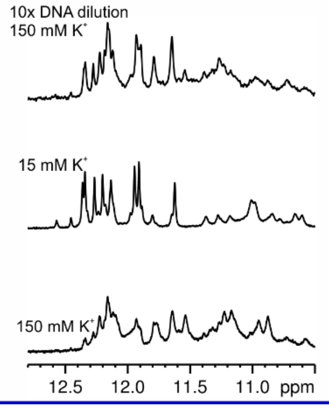

Remarks:
- NMR conditions - mixture of multi- and unimolecular form.

- PAGE conditions - one medium migrating band.

- NMR with dilution of DNA - two unimolecular forms dominate.

- NMR with dilution of K^+ - a single unimolecular form dominates.

- UV melting - particularly low T_m, preventing NMR structure determination.

Figure S1_62

62 (**442**): 5' GG**TTTT**GG**TTTT**GG**TT**GG 3' 18nt

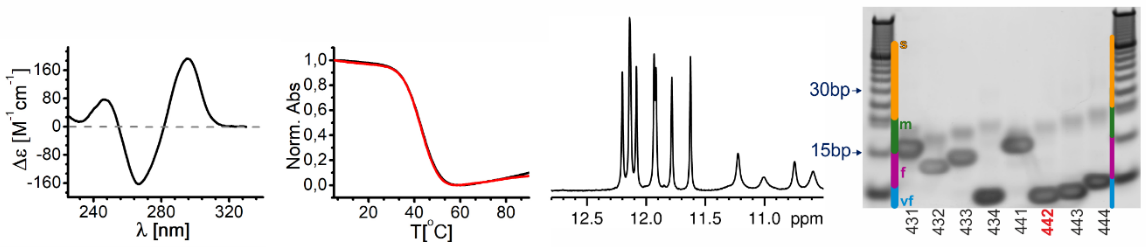

Remarks:
- NMR conditions - a single unimolecular form (-ld+l topology).

Figure S1_63

63 (**443**): 5' GG**TTTT**GG**TTTT**GG**TTT**GG 3' 19 nt

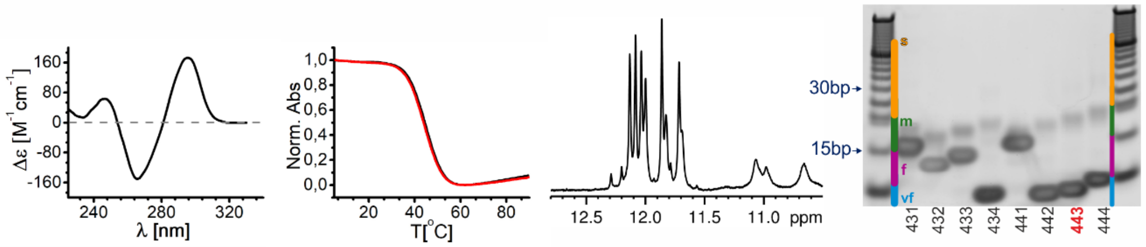

Remarks: NMR conditions - a single unimolecular form (-ld+l topology) dominates.

Figure S1_64

64 (**444**): 5' GG**TTTT**GG**TTTT**GG**TTTT**GG 3' 20 nt

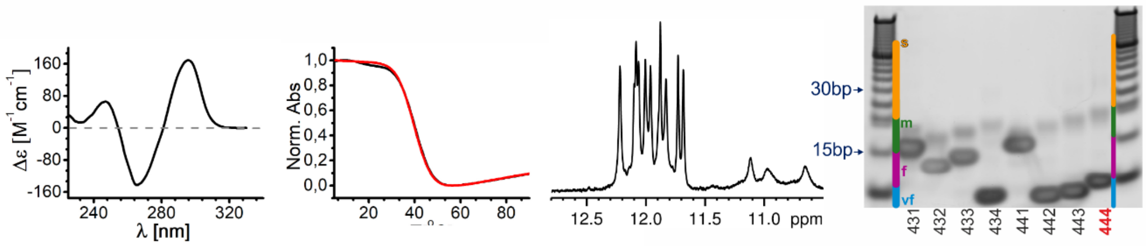

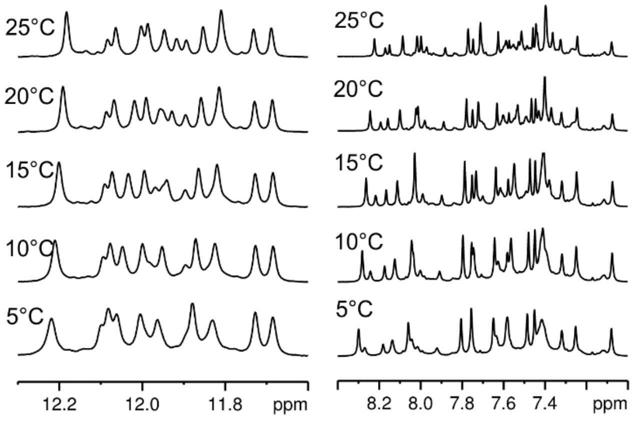

Remarks:
- NMR conditions – a mixture of unimolecular forms.

- PAGE conditions - one fast migrating band.

Supplementary Figure S2. UV melting curves (measured at 295 nm) of the 64 sequences studied. Black curve represents the profile measured from 5 °C to 90 °C for samples incubated at 5 °C for two weeks, while the red curve corresponds to renaturation immediately after melting.

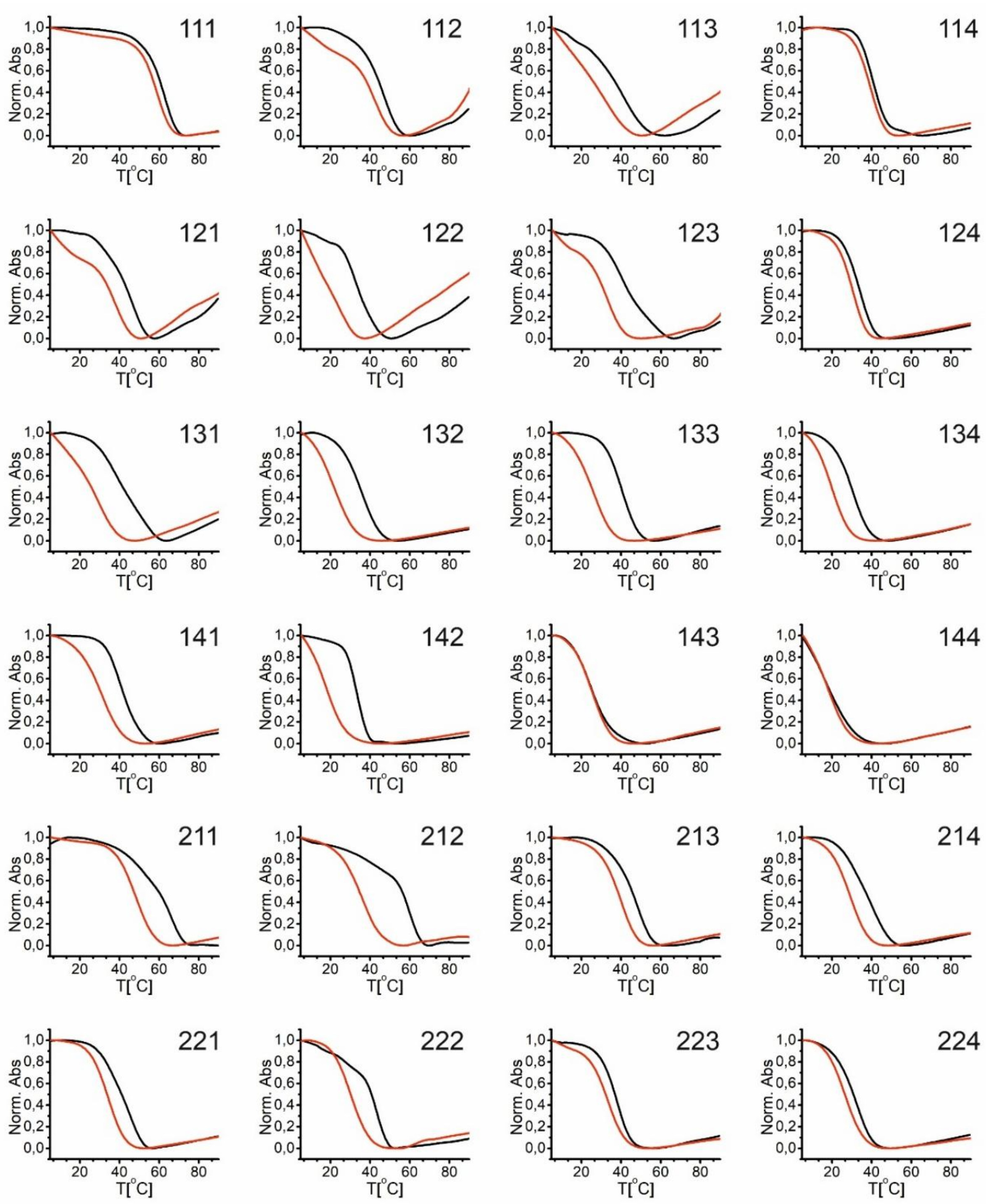

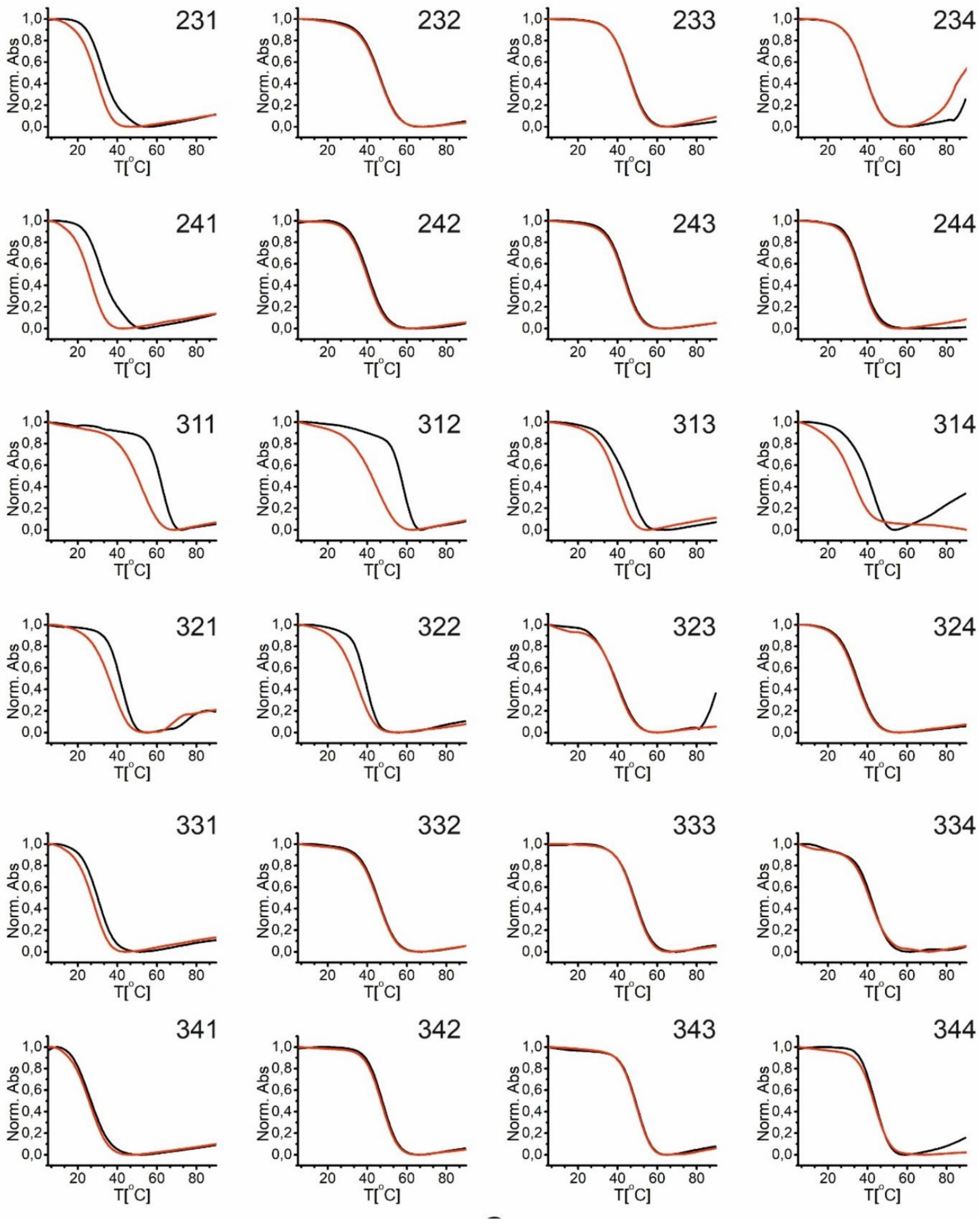
231
232
233
234
241
242
243
244
311
312
313
314
321
322
323
324
331
332
333
334
341
342
343
344
Norm. Abs
1,0
0,8
0,6
0,4
0,2
0,0
20
40
60
80
T[°C]

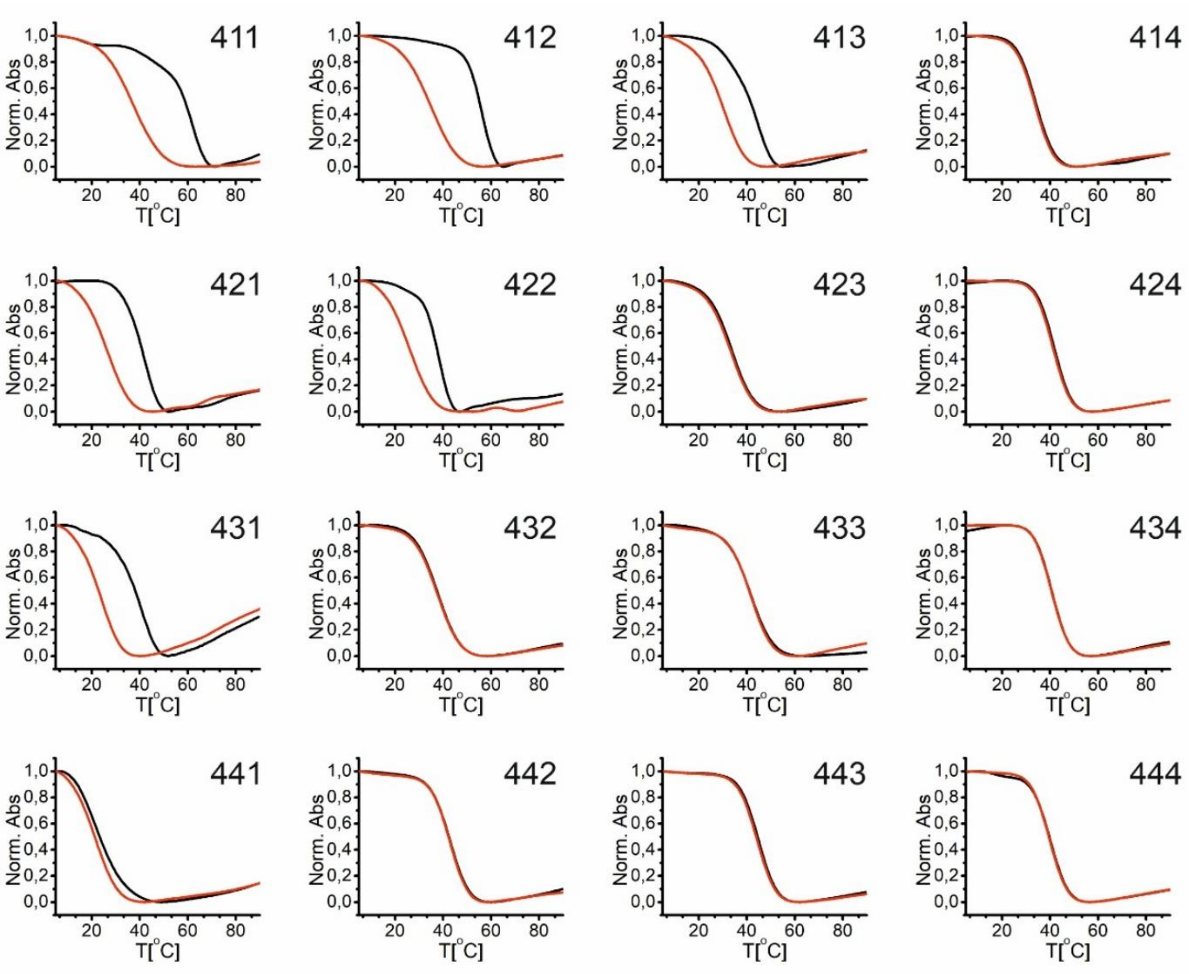
411
412
413
414
421
422
423
424
431
432
433
434
441
442
443
444
Norm. Abs
1,0
0,8
0,6
0,4
0,2
0,0
20
40
60
80
T[°C]

Supplementary Figure S3. The origin of Circular Dichroism spectral shapes for G4 structures. A) Two stacking arrangements of G-tetrads (homopolar and heteropolar) and the CD profiles they produce, B) G-tetrad stacking in parallel, antiparallel and hybrid G4s composed of three tetrads, C) G-tetrad stacking in parallel, antiparallel and hybrid two-tetrads G4s.

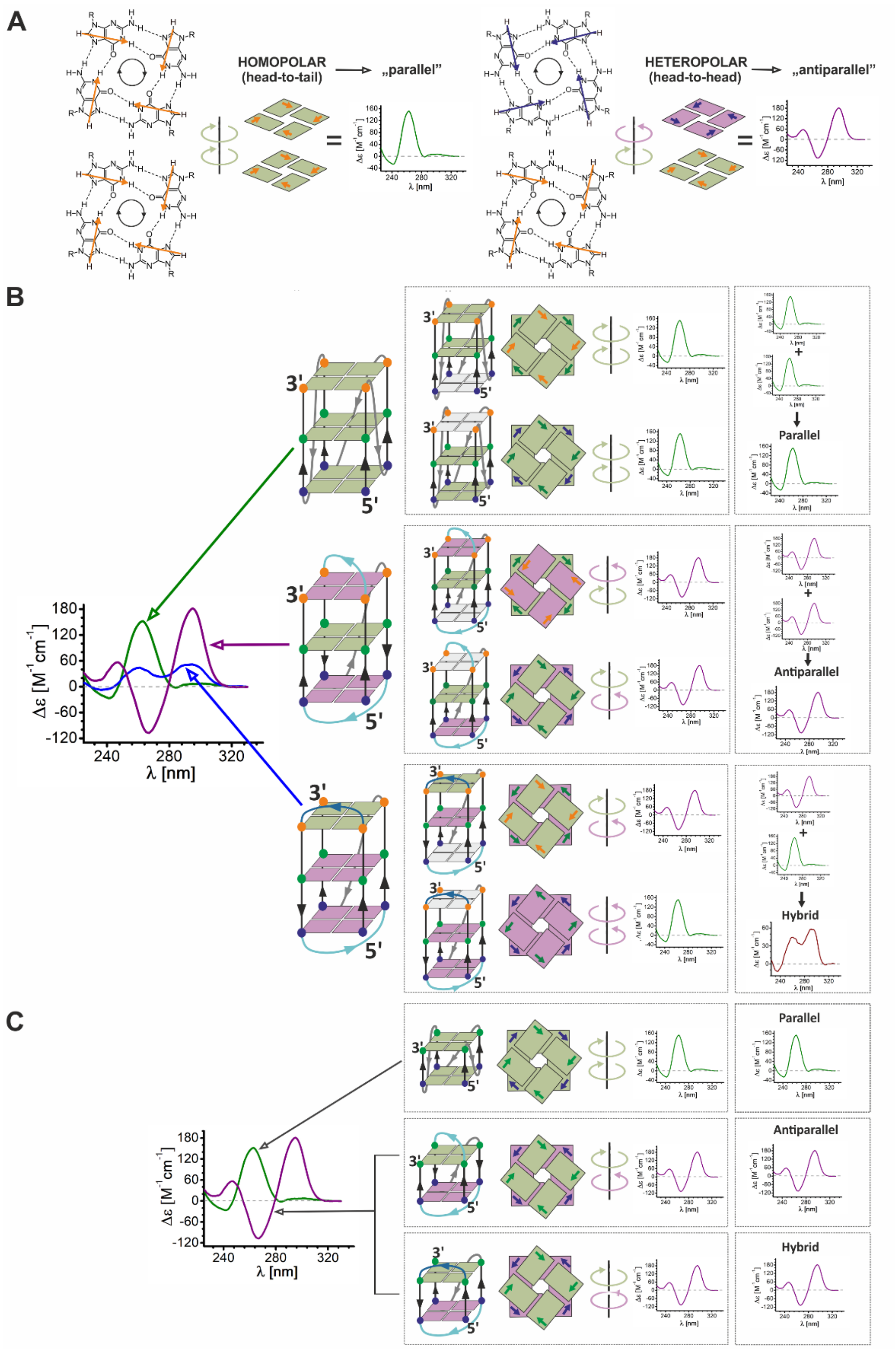

Supplementary Figure S4. Circular Dichroism spectra of the 64 studied sequences

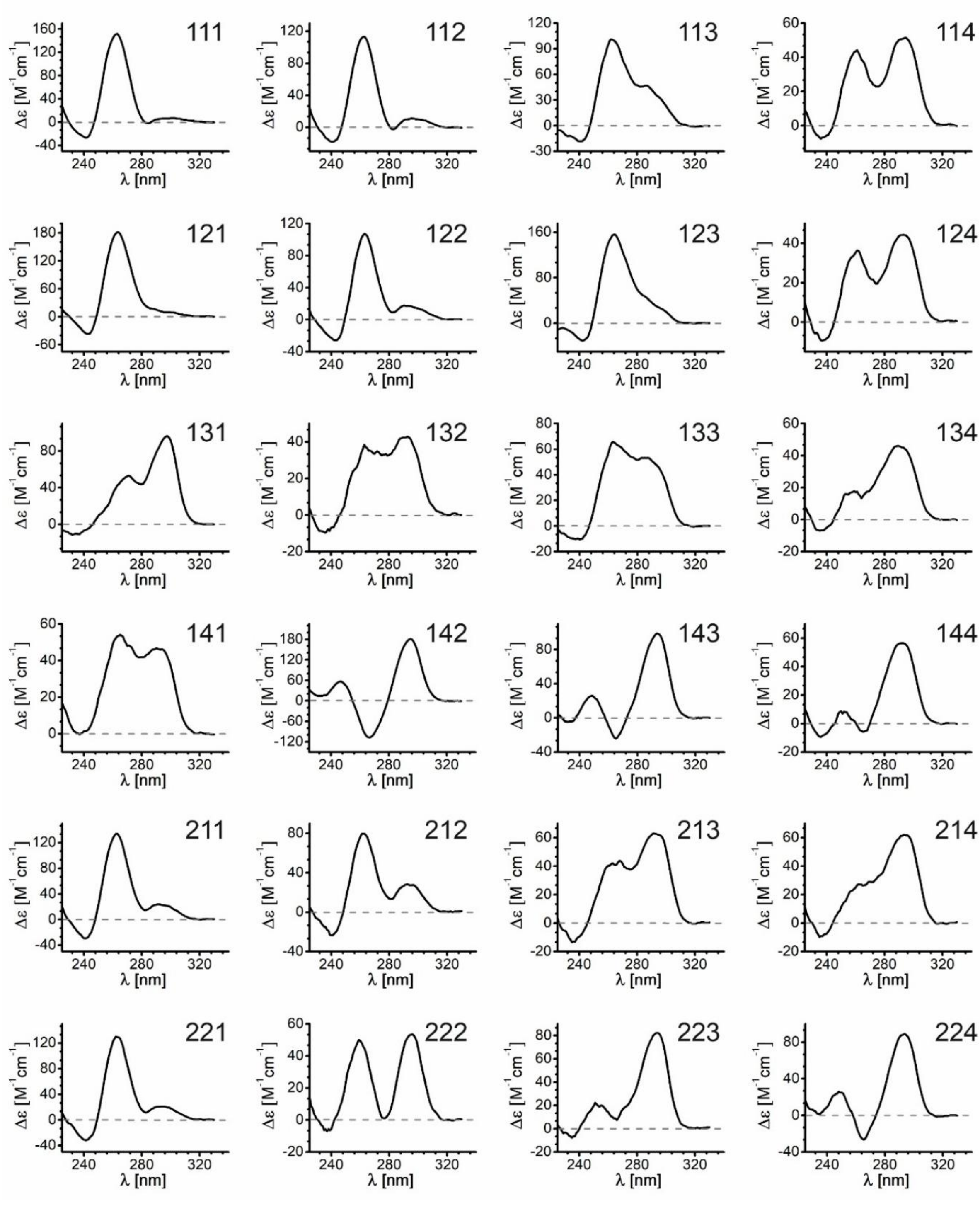

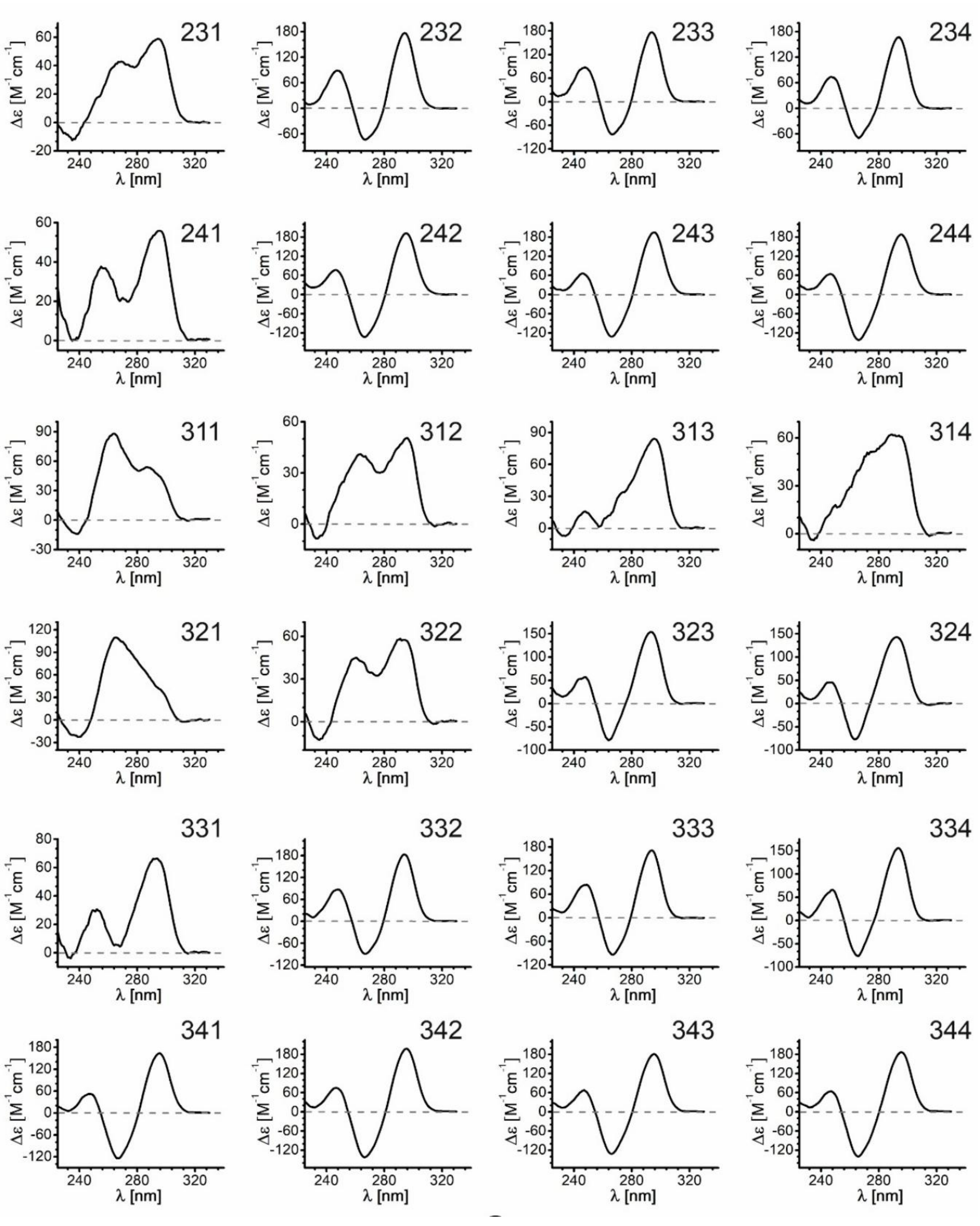

231
232
233
234
241
242
243
244
311
312
313
314
321
322
323
324
331
332
333
334
341
342
343
344
Δε [M-1cm-1]
λ [nm]

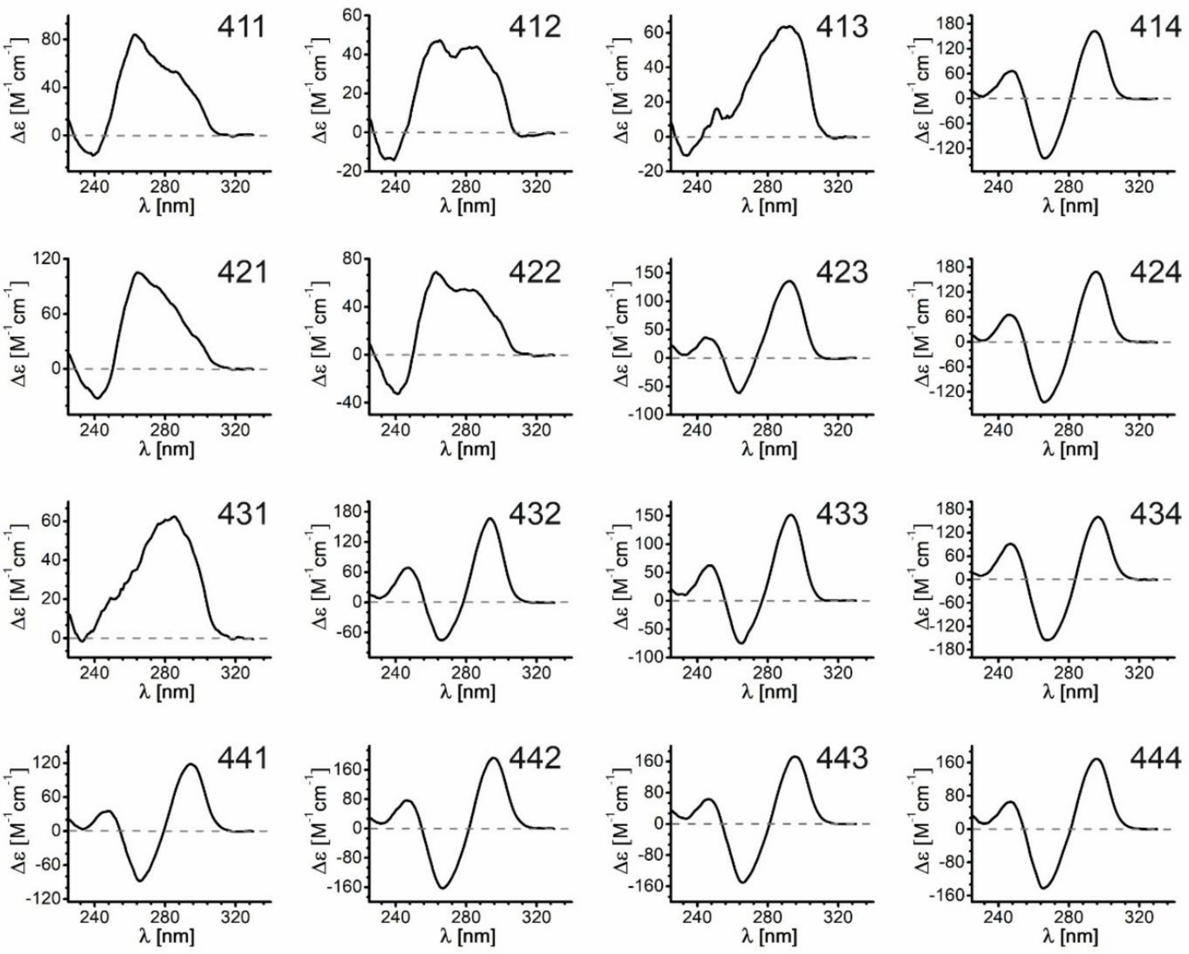
411
412
413
414
421
422
423
424
431
432
433
434
441
442
443
444
Δε [M-1 cm-1]
λ [nm]
240
280
320

Supplementary Figure S5. Principal component Analysis (PCA) of the obtained set of 64 CD spectra. Each point is colored according to results of an independent hierarchical clustering analysis (see text).

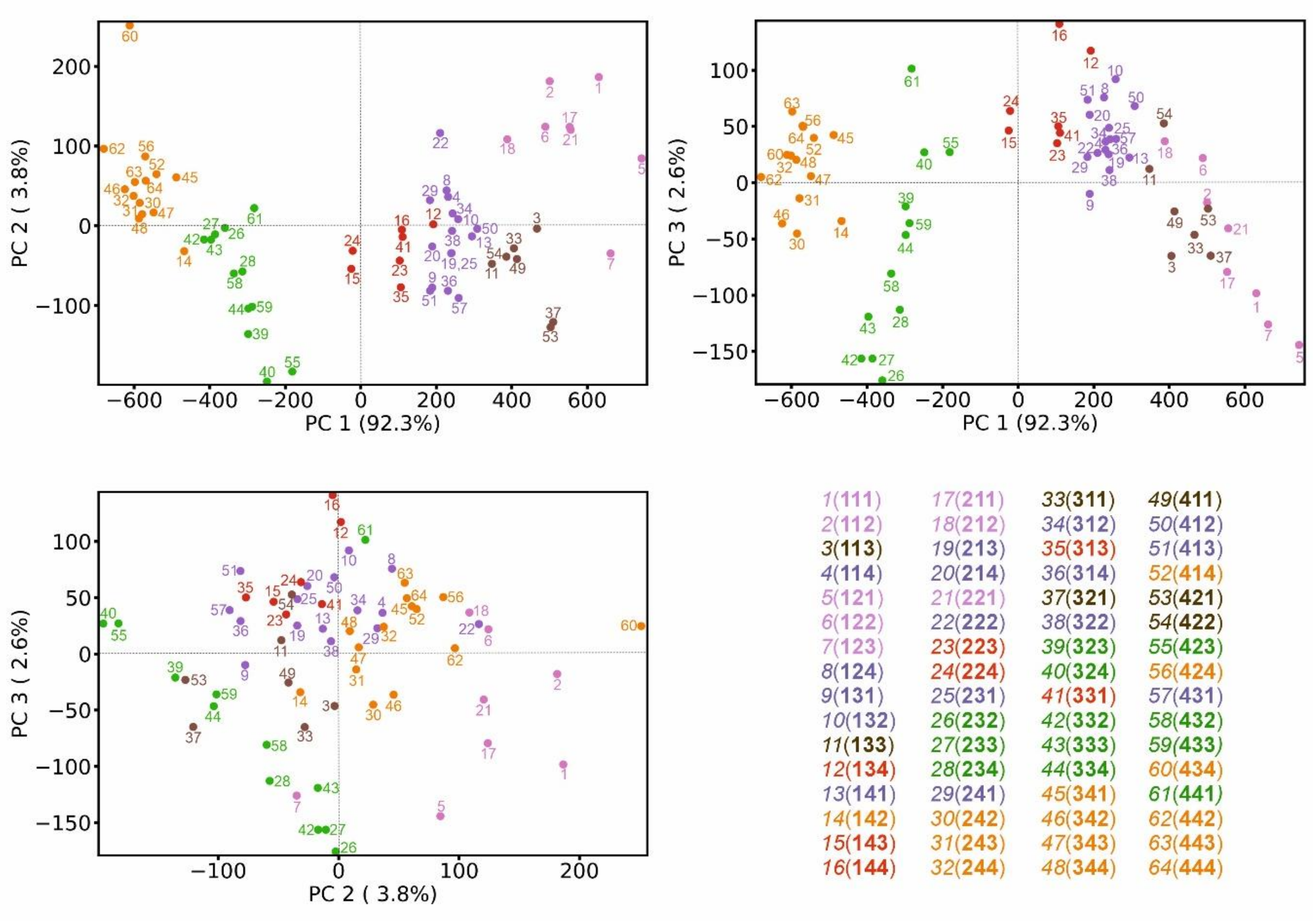

Supplementary Figure S6. Imino proton regions of 1D ^{1}H NMR spectra of 64 studied molecules. The spectral regions characteristic for G4s of different molecularities (see main text) are separated by a dotted blue line.

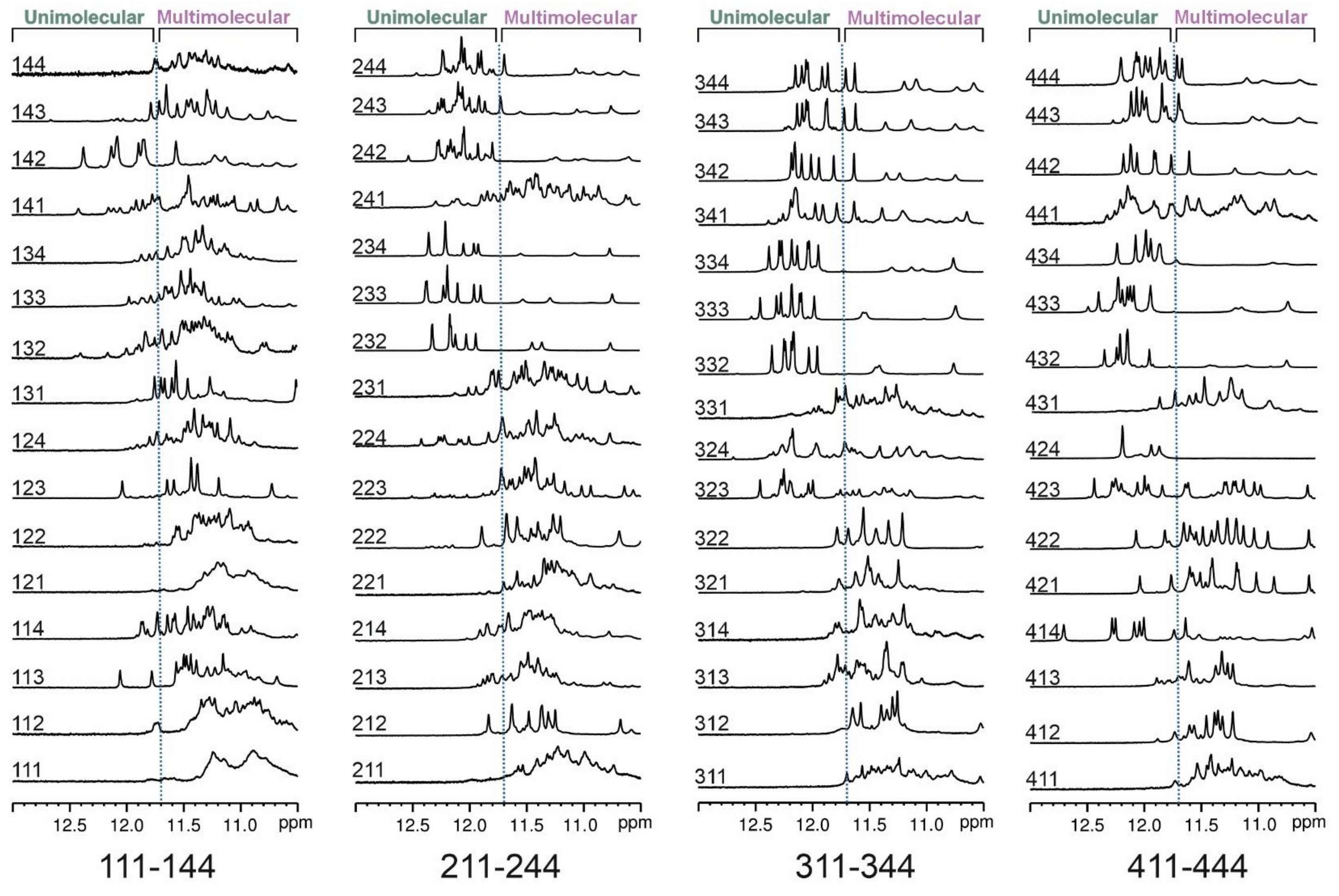

Supplementary Figure S7. Imino proton regions of ^{1}H NMR spectra of the studied set of 64 molecules measured after H_2O to D_2O solvent exchange. The spectra in D_2O are shown immediately after solvent exchange (~ 10 min) or after at least 24 h. Names of systems belonging to the initial set used to establish the method (see main text) are marked by a black background.

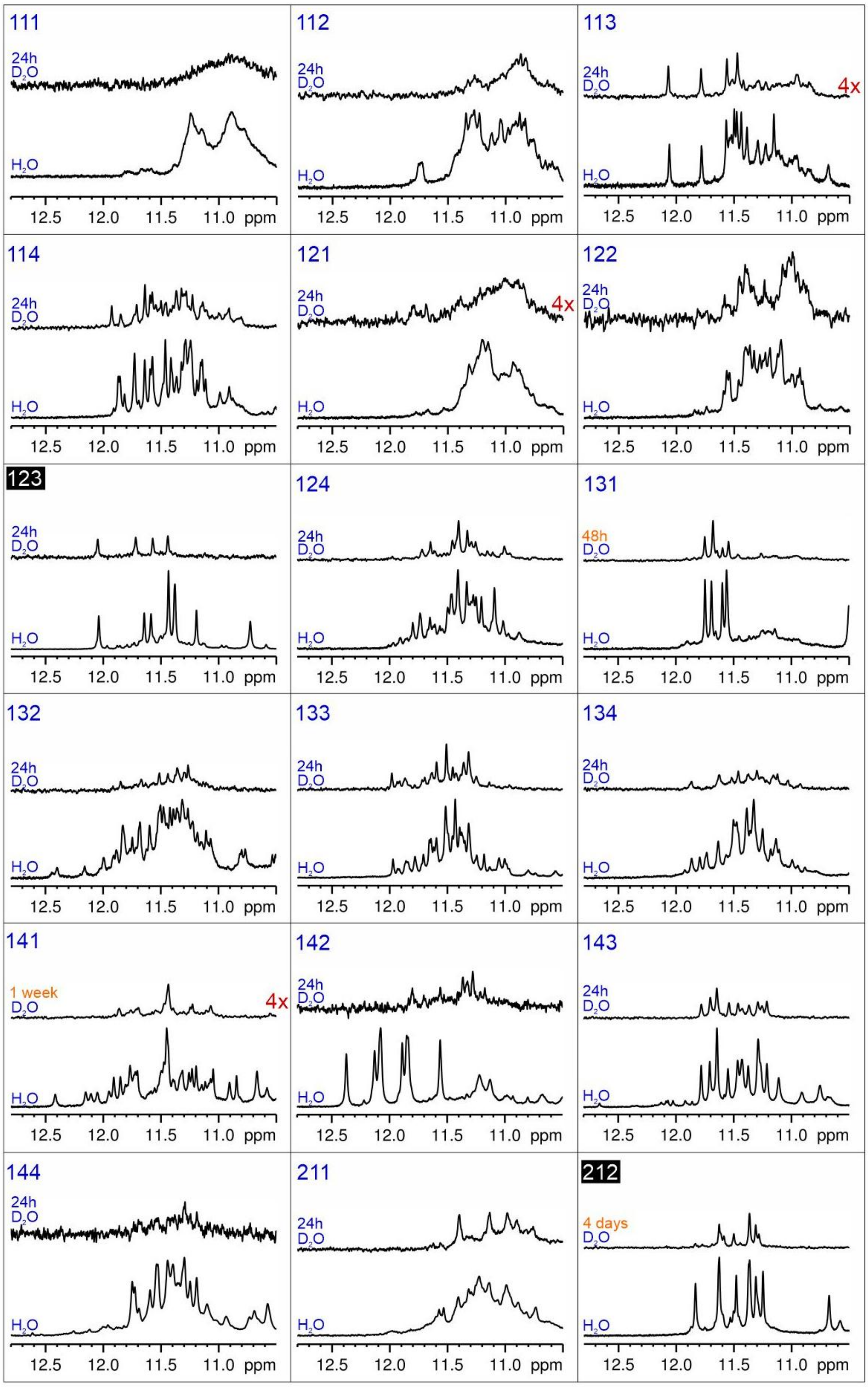

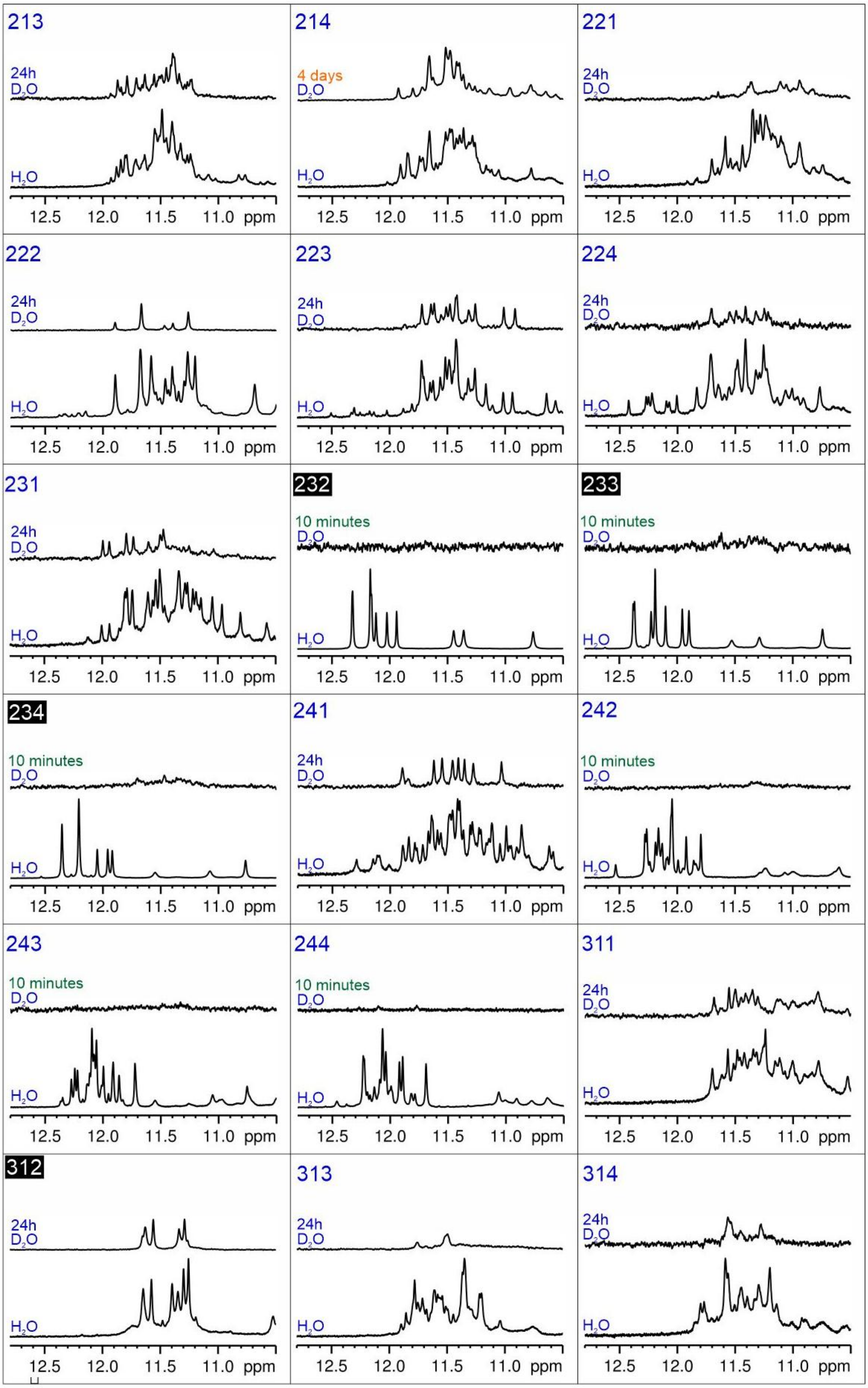

213
24h
D_2O
H_2O
12.5 12.0 11.5 11.0 ppm
214
4 days
D_2O
H_2O
12.5 12.0 11.5 11.0 ppm
221
24h
D_2O
H_2O
12.5 12.0 11.5 11.0 ppm
222
24h
D_2O
H_2O
12.5 12.0 11.5 11.0 ppm
223
24h
D_2O
H_2O
12.5 12.0 11.5 11.0 ppm
224
24h
D_2O
H_2O
12.5 12.0 11.5 11.0 ppm
231
24h
D_2O
H_2O
12.5 12.0 11.5 11.0 ppm
232
10 minutes
D_2O
H_2O
12.5 12.0 11.5 11.0 ppm
233
10 minutes
D_2O
H_2O
12.5 12.0 11.5 11.0 ppm
234
10 minutes
D_2O
H_2O
12.5 12.0 11.5 11.0 ppm
241
24h
D_2O
H_2O
12.5 12.0 11.5 11.0 ppm
242
10 minutes
D_2O
H_2O
12.5 12.0 11.5 11.0 ppm
243
10 minutes
D_2O
H_2O
12.5 12.0 11.5 11.0 ppm
244
10 minutes
D_2O
H_2O
12.5 12.0 11.5 11.0 ppm
311
24h
D_2O
H_2O
12.5 12.0 11.5 11.0 ppm
312
24h
D_2O
H_2O
12.5 12.0 11.5 11.0 ppm
313
24h
D_2O
H_2O
12.5 12.0 11.5 11.0 ppm
314
24h
D_2O
H_2O
12.5 12.0 11.5 11.0 ppm

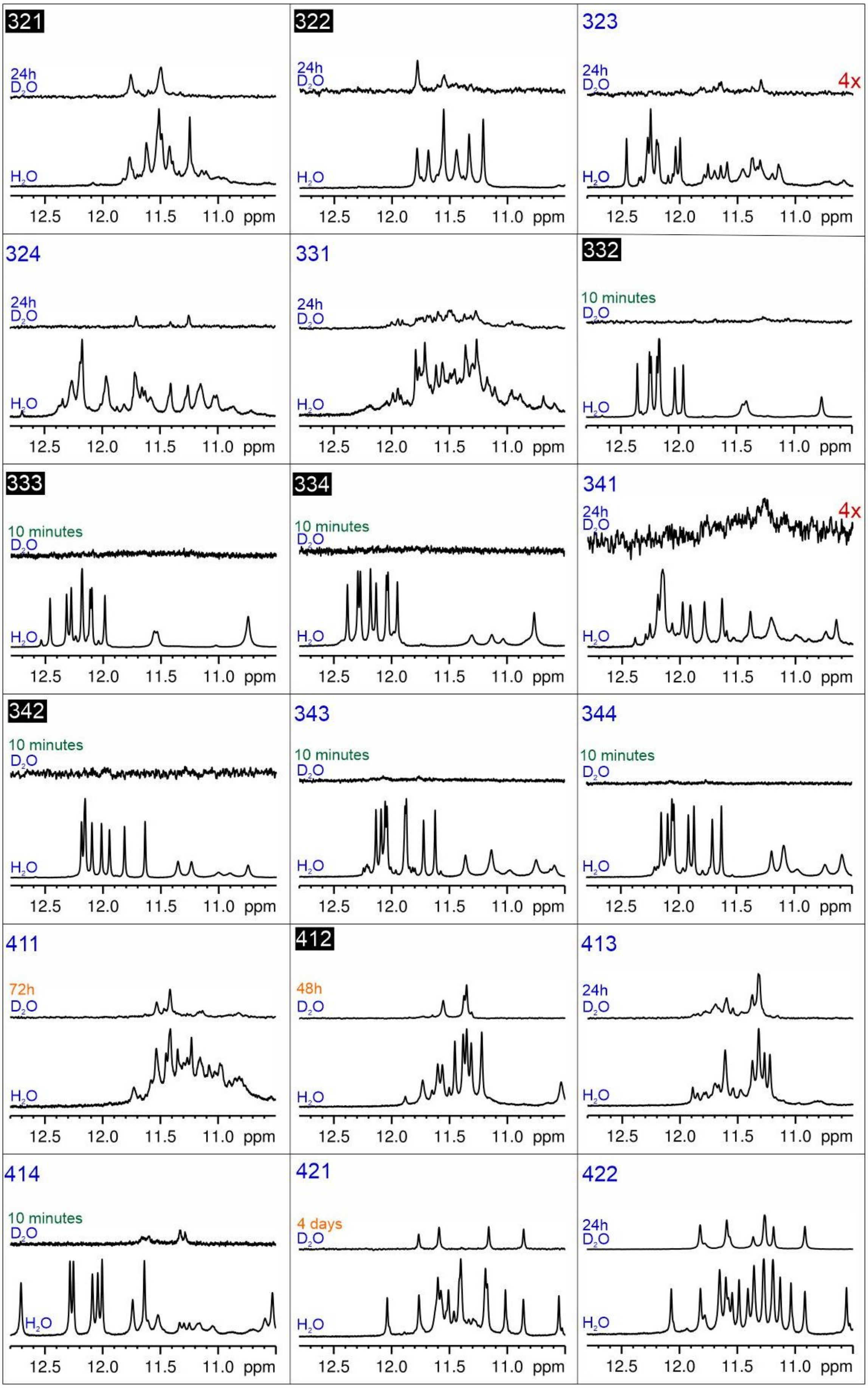

321
24h
D2O
H2O
12.5 12.0 11.5 11.0 ppm
322
24h
D2O
H2O
12.5 12.0 11.5 11.0 ppm
323
24h
D2O
4x
H2O
12.5 12.0 11.5 11.0 ppm
324
24h
D2O
H2O
12.5 12.0 11.5 11.0 ppm
331
24h
D2O
H2O
12.5 12.0 11.5 11.0 ppm
332
10 minutes
D2O
H2O
12.5 12.0 11.5 11.0 ppm
333
10 minutes
D2O
H2O
12.5 12.0 11.5 11.0 ppm
334
10 minutes
D2O
H2O
12.5 12.0 11.5 11.0 ppm
341
24h
D2O
4x
H2O
12.5 12.0 11.5 11.0 ppm
342
10 minutes
D2O
H2O
12.5 12.0 11.5 11.0 ppm
343
10 minutes
D2O
H2O
12.5 12.0 11.5 11.0 ppm
344
10 minutes
D2O
H2O
12.5 12.0 11.5 11.0 ppm
411
72h
D2O
H2O
12.5 12.0 11.5 11.0 ppm
412
48h
D2O
H2O
12.5 12.0 11.5 11.0 ppm
413
24h
D2O
H2O
12.5 12.0 11.5 11.0 ppm
414
10 minutes
D2O
H2O
12.5 12.0 11.5 11.0 ppm
421
4 days
D2O
H2O
12.5 12.0 11.5 11.0 ppm
422
24h
D2O
H2O
12.5 12.0 11.5 11.0 ppm

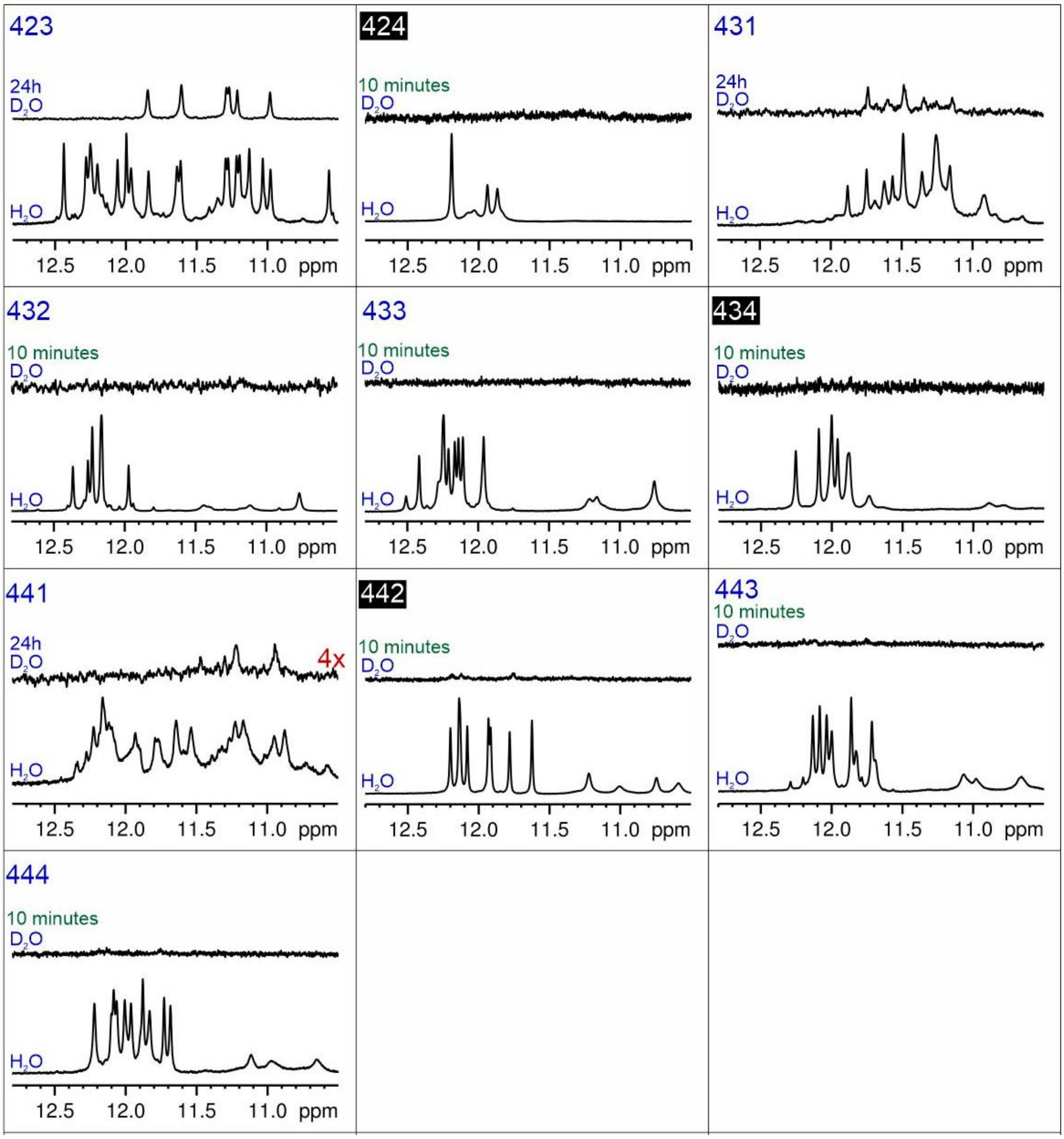

423
24h
D_2O
H_2O
12.5 12.0 11.5 11.0 ppm
424
10 minutes
D_2O
H_2O
12.5 12.0 11.5 11.0 ppm
431
24h
D_2O
H_2O
12.5 12.0 11.5 11.0 ppm
432
10 minutes
D_2O
H_2O
12.5 12.0 11.5 11.0 ppm
433
10 minutes
D_2O
H_2O
12.5 12.0 11.5 11.0 ppm
434
10 minutes
D_2O
H_2O
12.5 12.0 11.5 11.0 ppm
441
24h
D_2O
4x
H_2O
12.5 12.0 11.5 11.0 ppm
442
10 minutes
D_2O
H_2O
12.5 12.0 11.5 11.0 ppm
443
10 minutes
D_2O
H_2O
12.5 12.0 11.5 11.0 ppm
444
10 minutes
D_2O
H_2O
12.5 12.0 11.5 11.0 ppm

Supplementary Figure S8. Assignment of G4 topologies based on 2D NMR data. The contents of each panel (one per system with determined loop topology) are described in the section "G4 topology assignment" of the Supplementary Information. Green and violet rectangles indicate G residues in *syn* and *anti* conformations, respectively.

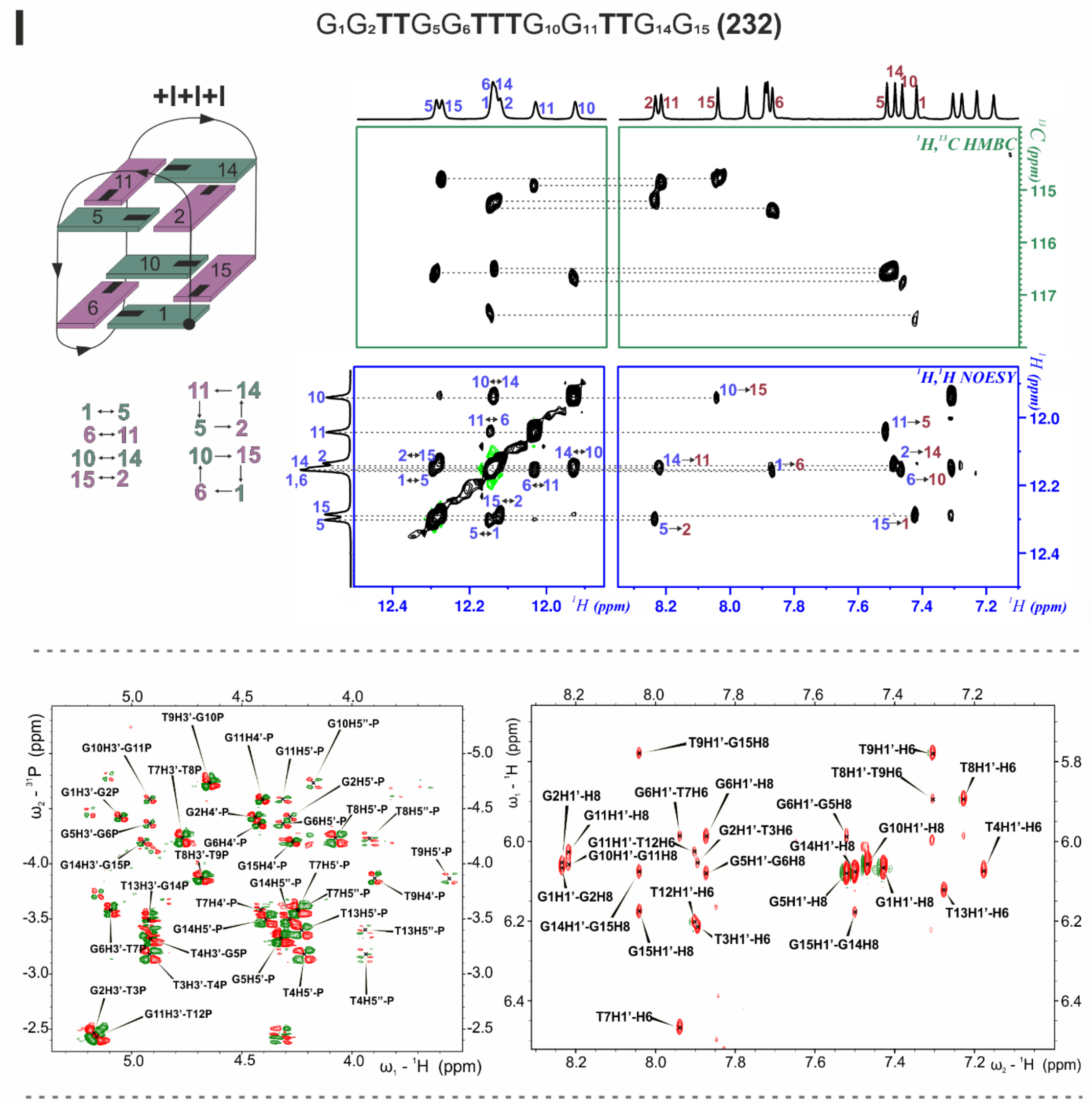

	H1'	H2'	H2"	H3'	H4'	H5'	H5"	H6/H8	Me	P	HN	C5
**G1**	6.066	2.858	2.987	5.048	4.398	3.972	3.972	7.422	-	-	12.15	117.3
**G2**	6.053	3.058	2.365	5.172	4.441	4.280	-	8.235	-	-4.425	12.13	115.2
**T3**	6.213	2.240	2.603	4.918	4.309	3.940	-	7.893	1.995	-2.462	-	-
**T4**	6.073	2.081	2.673	4.915	4.314	4.216	3.937	7.176	1.058	-3.180	-	-
**G5**	6.080	3.476	2.974	4.918	4.445	4.322	-	7.518	-	-3.322	12.29	116.6
**G6**	5.987	2.682	2.366	5.094	4.420	4.312	4.247	7.872	-	-4.362	12.15	115.3
**T7**	6.466	2.438	2.610	4.769	4.416	4.248	4.243	7.939	1.995	-3.580	-	-
**T8**	5.894	1.797	2.165	4.682	3.835	4.074	3.922	7.227	1.801	-4.233	-	-
**T9**	5.780	1.974	2.479	4.646	3.898	3.557	2.993	7.304	1.817	-3.867	-	-
**G10**	6.057	3.725	2.942	4.918	4.430	4.289	4.175	7.467	-	-4.734	11.93	116.7
**G11**	6.026	2.978	2.338	5.156	4.407	4.316	-	8.218	-	-4.586	12.03	114.9
**T12**	6.203	2.218	2.598	4.928	4.298	3.941	-	7.902	2.003	-2.429	-	-
**T13**	6.120	2.109	2.733	4.922	4.305	4.231	3.942	7.276	0.9750	-3.398	-	-
**G14**	6.076	3.519	2.978	4.943	4.442	4.391	4.285	7.495	-	-3.493	12.14	116.5
**G15**	6.175	2.683	2.472	4.810	4.268	-	4.206	8.040	-	-4.199	12.28	114.8

II G_1G_2**TT**G_5G_6**TTT**$G_{10}G_{11}$**TTT**$G_{15}G_{16}$ **(233)**

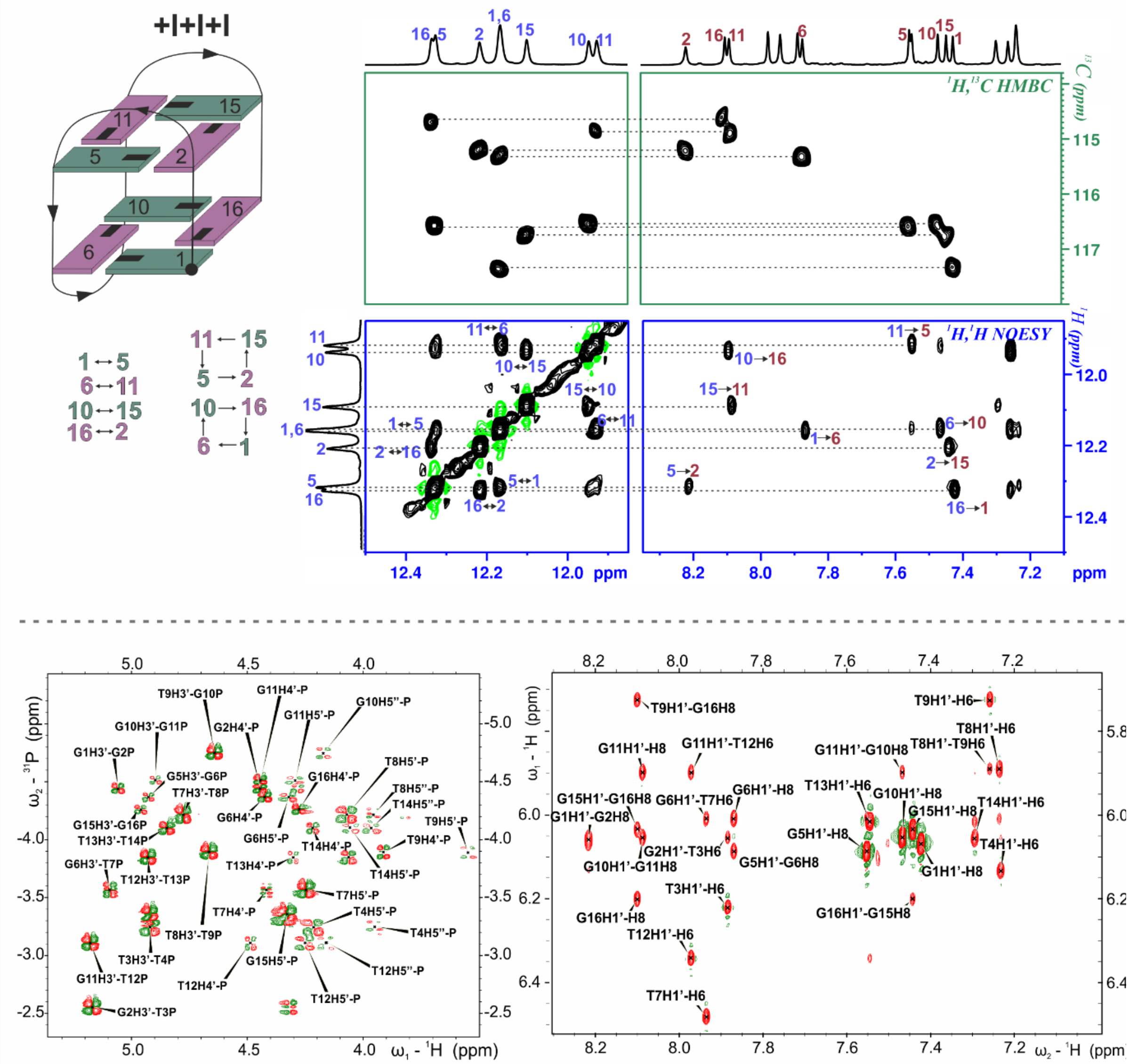

	H1'	H2'	H2''	H3'	H4'	H5'	H5''	H6/H8	Me	P	HN	C5
**G1**	6.064	2.843	2.994	5.058	4.399	3.974	3.974	7.423	-	-	12.16	117.3
**G2**	6.054	3.039	2.380	5.169	4.446	-	-	8.216	-	-4.442	12.21	115.2
**T3**	6.220	2.218	2.573	4.921	4.316	-	-	7.884	1.995	-2.554	-	-
**T4**	6.131	2.111	2.691	4.926	4.329	4.213	3.949	7.232	1.108	-3.247	-	-
**G5**	6.086	3.482	2.976	4.926	4.460	4.360	-	7.553	-	-	12.32	116.6
**G6**	6.008	2.660	2.365	5.093	4.426	4.324	4.251	7.870	-	-4.368	12.15	115.3
**T7**	6.480	2.471	2.646	4.778	4.419	4.247	-	7.935	1.993	-3.568	-	-
**T8**	5.890	1.796	2.135	4.665	3.821	4.069	3.958	7.236	1.803	-4.216	-	-
**T9**	5.725	2.003	2.509	4.643	3.914	3.547	-	7.259	1.819	-3.893	-	-
**G10**	6.053	3.715	2.885	4.897	4.438	4.285	4.171	7.467	-	-4.748	11.94	116.5
**G11**	5.898	2.699	2.289	5.179	4.443	4.293	4.255	8.087	-	-4.514	11.92	114.9
**T12**	6.340	2.070	2.370	4.929	4.488	4.251	4.158	7.972	2.136	-3.111	-	-
**T13**	6.015	1.988	2.428	4.846	4.303	4.063	-	7.544	1.730	-3.847	-	-
**T14**	6.056	2.340	2.526	4.921	4.219	4.059	3.965	7.295	1.092	-4.102	-	-
**G15**	6.034	3.462	2.869	4.960	4.444	4.328	-	7.444	-	-3.358	12.09	116.8
**G16**	6.201	2.708	2.471	4.809	4.277	4.256	4.191	8.101	-	-4.261	12.32	114.7

III $G_1G_2TTG_5G_6TTTG_{10}G_{11}TTTTG_{16}G_{17}$ **(234)**

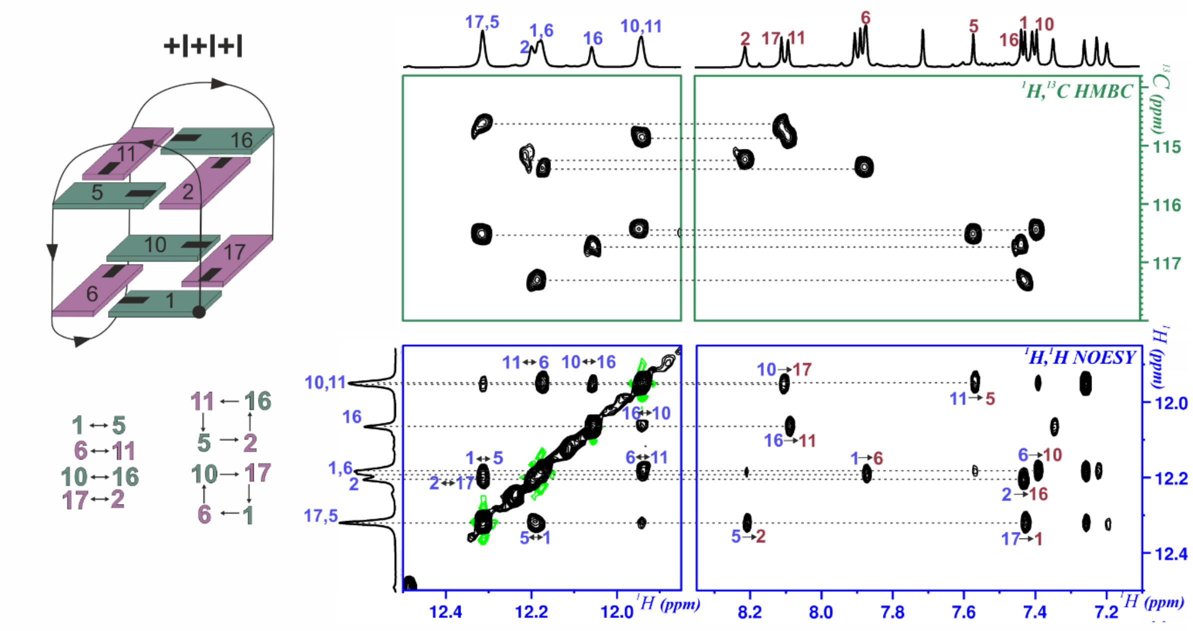

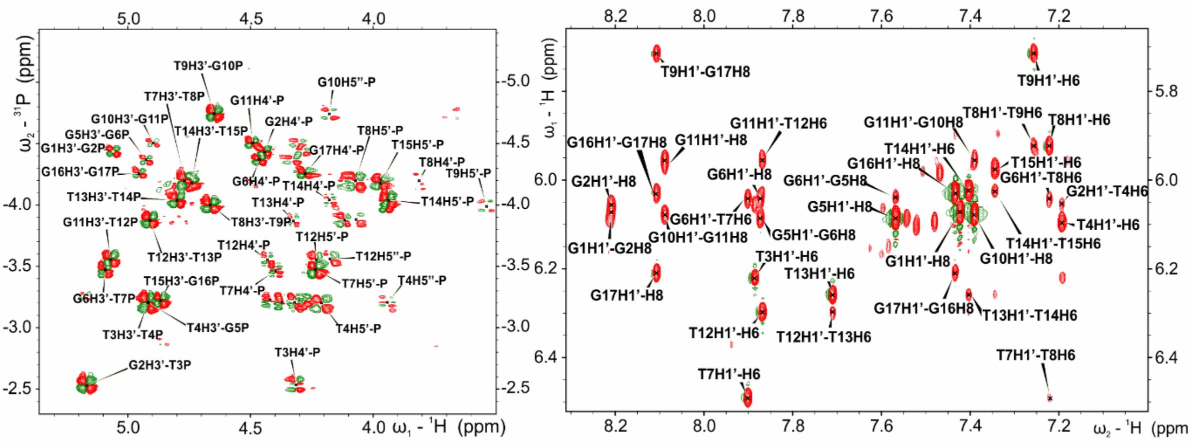

	H1'	H2'	H2''	H3'	H4'	H5'	H5''	H6/H8	Me	P	HN	C5
**G1**	6.072	2.842	3.009	5.060	4.402	3.976	3.976	7.424	-	-	12.19	117.3
**G2**	6.055	3.023	2.374	5.168	4.444	4.300	4.278	8.210	-	-4.436	12.21	115.2
**T3**	6.221	2.221	2.576	4.918	4.312	-	-	7.886	1.999	-2.533	-	-
**T4**	6.096	2.091	2.688	4.916	-	4.210	3.941	7.193	1.105	-3.204	-	-
**G5**	6.086	3.465	2.983	4.924	4.466	4.373	4.329	7.568	-	-3.204	12.31	116.5
**G6**	6.042	2.666	2.389	5.091	4.463	4.335	4.262	7.873	-	-4.370	12.18	115.4
**T7**	6.491	2.529	2.633	4.767	4.397	4.234	-	7.901	1.995	-3.471	-	-
**T8**	5.924	1.784	2.139	4.664	3.814	4.070	3.964	7.221	1.790	-4.198	-	-
**T9**	5.715	2.007	2.515	4.647	3.954	3.540	2.938	7.256	1.822	-3.991	-	-
**G10**	6.077	3.748	2.921	4.897	4.448	4.303	4.177	7.390	-	-4.744	11.95	116.4
**G11**	5.955	2.555	2.385	5.071	4.491	4.305	4.258	8.089	-	-4.505	11.94	114.9
**T12**	6.298	2.308	2.507	4.910	4.434	4.231	4.176	7.868	2.040	-3.556	-	-
**T13**	6.259	2.237	2.409	4.802	4.327	4.115	4.065	7.710	1.925	-3.873	-	-
**T14**	6.024	2.079	2.367	4.742	4.177	3.937	-	7.404	1.672	-4.040	-	-
**T15**	5.976	2.316	2.491	4.868	4.119	3.972	-	7.344	1.275	-4.178	-	-
**G16**	6.030	3.441	2.862	4.953	4.439	4.429	4.286	7.433	-	-3.224	12.06	116.7
**G17**	6.210	2.702	2.476	4.808	4.279	4.258	4.191	8.107	-	-4.256	12.32	114.6

IV

G_1G_2**TTT**G_6G_7**TTT**$G_{11}G_{12}$**TT**$G_{15}G_{16}$ **(332)**

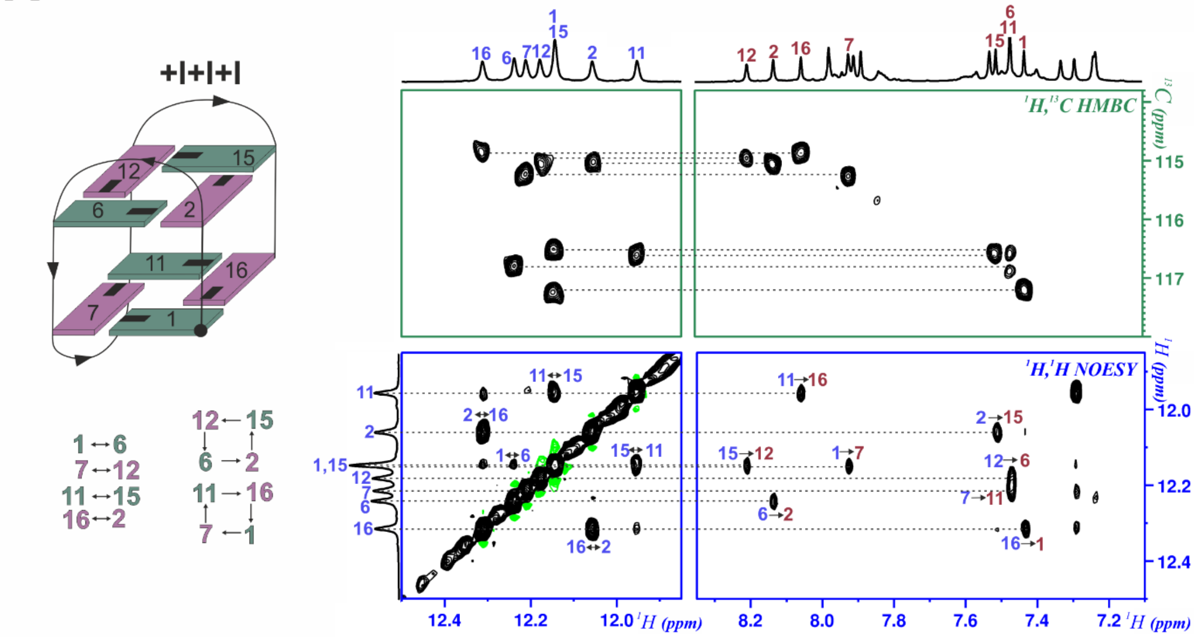

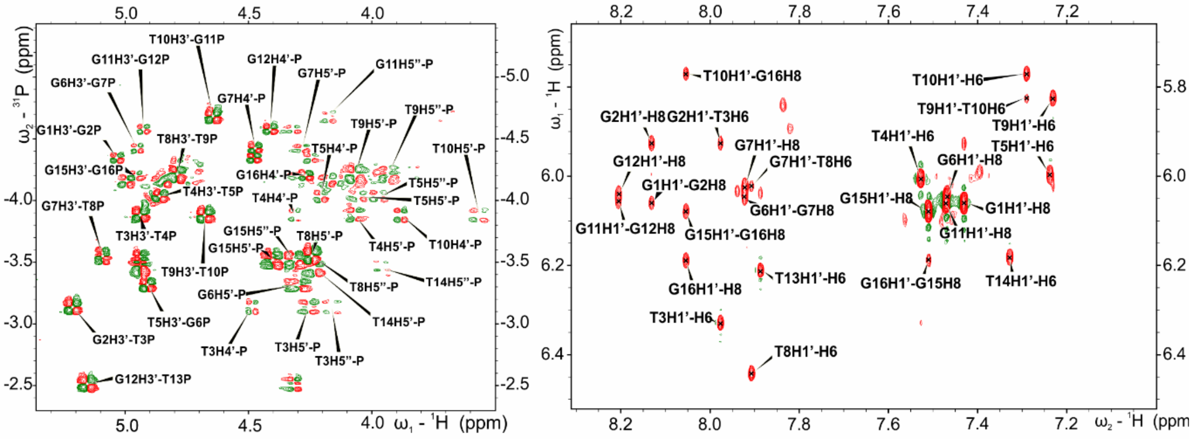

	H1'	H2'	H2''	H3'	H4'	H5'	H5''	H6/H8	Me	P	HN	C5
**G1**	6.060	2.755	2.942	5.032	4.405	3.972	3.972	7.430	-	-	12.15	117.2
**G2**	5.926	2.779	2.332	5.213	4.478	4.249	-	8.132	-	-4.346	12.06	115.1
**T3**	6.329	2.082	2.380	4.939	4.485	4.259	4.160	7.976	2.135	-3.138	-	-
**T4**	6.006	2.017	2.449	4.865	4.312	4.066	-	7.527	1.721	-3.881	-	-
**T5**	5.995	2.326	2.491	4.909	4.207	4.058	3.970	7.238	1.097	-4.032	-	-
**G6**	6.047	3.418	2.854	4.950	4.436	4.292	-	7.468	-	-3.317	12.24	116.7
**G7**	6.024	2.701	2.351	5.091	4.472	4.282	4.231	7.922	-	-4.422	12.21	115.2
**T8**	6.442	2.448	2.604	4.790	4.406	4.243	4.243	7.907	1.977	-3.552	-	-
**T9**	5.825	-	2.140	4.676	3.798	4.068	3.933	7.231	1.817	-4.211	-	-
**T10**	5.771	1.996	2.484	4.643	3.883	3.564	3.026	7.290	1.790	-3.883	-	-
**G11**	6.059	3.705	2.942	4.925	4.436	4.309	4.175	7.471	-	-4.686	11.95	116.7
**G12**	6.040	2.956	2.367	5.153	4.412	4.312	4.292	8.205	-	-4.577	12.17	115.0
**T13**	6.211	2.212	2.578	4.936	4.311	-	-	7.887	1.994	-2.515	-	-
**T14**	6.181	2.145	2.750	4.938	4.327	4.231	3.960	7.328	1.005	-3.450	-	-
**G15**	6.079	3.524	2.975	4.955	4.459	4.401	4.318	7.511	-	-3.520	12.15	116.5
**G16**	6.188	2.682	2.469	4.814	4.267	4.297	4.207	8.054	-	-4.205	12.31	114.9

V

G$_1$G$_2$**TTT**G$_6$G$_7$**TTT**G$_{11}$G$_{12}$**TTT**G$_{16}$G$_{17}$ **(333)**

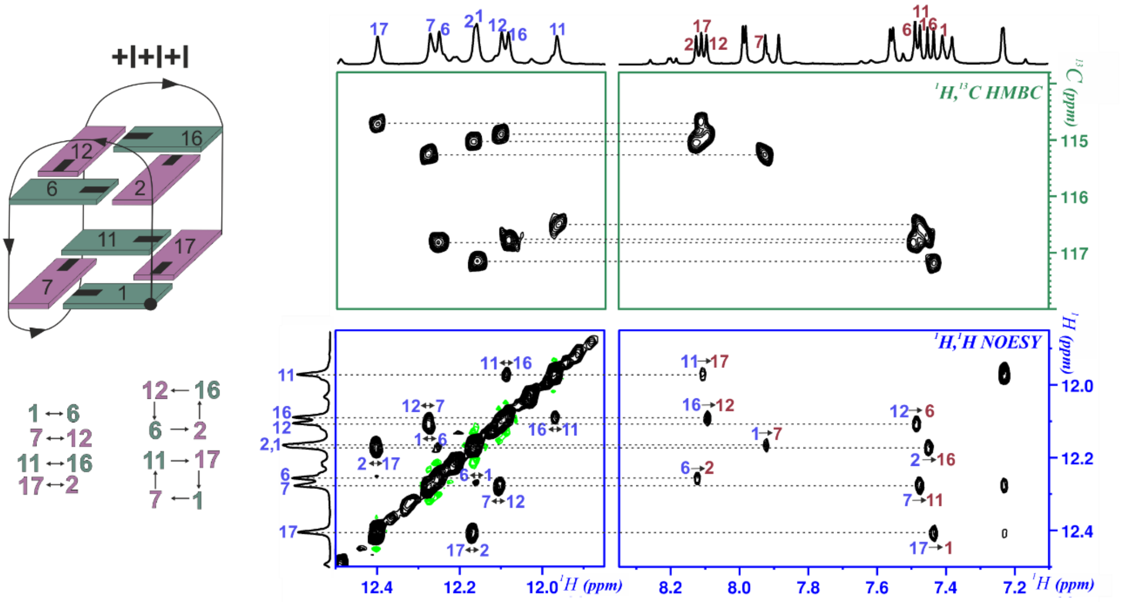

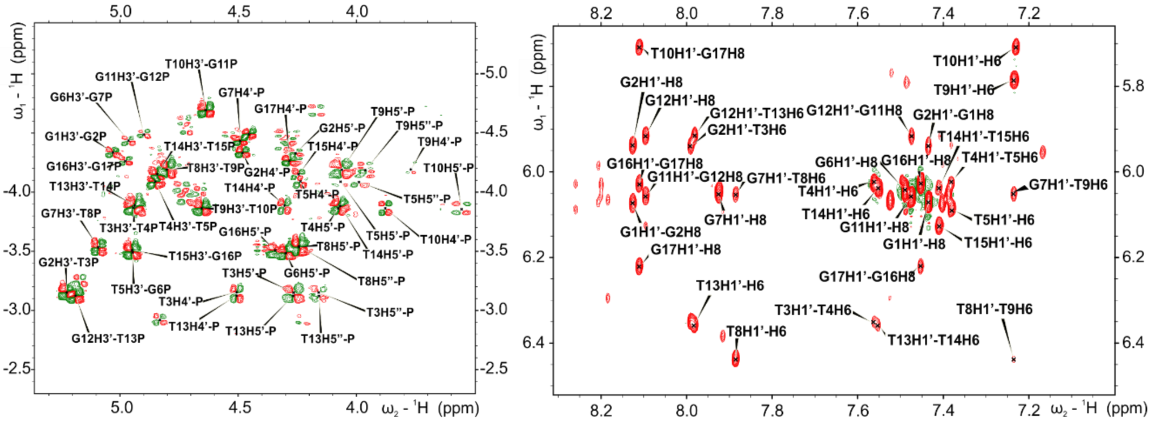

	H1'	H2'	H2''	H3'	H4'	H5'	H5''	H6/H8	Me	P	HN	C5
**G1**	6.072	2.720	2.959	5.028	4.409	4.248	3.974	7.435	-	-	12.16	117.2
**G2**	5.938	2.789	2.330	5.226	4.485	4.258	-	8.126	-	-4.334	12.17	115.0
**T3**	6.353	2.030	2.372	4.947	4.507	4.265	4.159	7.990	2.150	-3.153	-	-
**T4**	6.023	1.890	2.413	4.851	4.310	4.078	-	7.562	1.729	-3.867	-	-
**T5**	6.087	2.391	2.531	4.946	4.237	4.067	3.990	7.381	1.116	-4.088	-	-
**G6**	6.042	3.413	2.845	4.963	4.451	4.300	-	7.490	-	-3.487	12.26	116.8
**G7**	6.052	2.683	2.344	5.089	4.492	4.292	4.235	7.924	-	-4.435	12.28	115.3
**T8**	6.438	2.471	2.630	4.800	4.405	4.243	4.243	7.885	1.967	-3.537	-	-
**T9**	5.786	-	2.092	4.652	3.772	4.062	3.963	7.235	1.821	-4.189	-	-
**T10**	5.709	2.023	2.511	4.636	3.881	3.558	2.992	7.231	1.775	-3.862	-	-
**G11**	6.057	3.684	2.879	4.899	4.441	4.310	4.168	7.475	-	-4.693	11.97	116.5
**G12**	5.916	2.715	2.307	5.189	4.458	4.308	4.259	8.096	-	-4.488	12.10	114.9
**T13**	6.355	2.030	2.370	4.938	4.509	4.266	4.159	7.983	2.150	-3.124	-	-
**T14**	6.036	1.873	2.391	4.842	4.303	4.068	-	7.553	1.725	-3.885	-	-
**T15**	6.127	2.394	2.567	4.951	4.245	4.073	3.987	7.409	1.103	-4.152	-	-
**G16**	6.028	3.460	2.874	4.975	4.460	4.344	-	7.452	-	-3.508	12.09	116.7
**G17**	6.221	2.700	2.471	4.809	4.287	4.260	4.200	8.110	-	-4.264	12.40	114.7

VI

G_1G_2**TTT**G_6G_7**TTT**$G_{11}G_{12}$**TTTT**$G_{17}G_{18}$ **(334)**

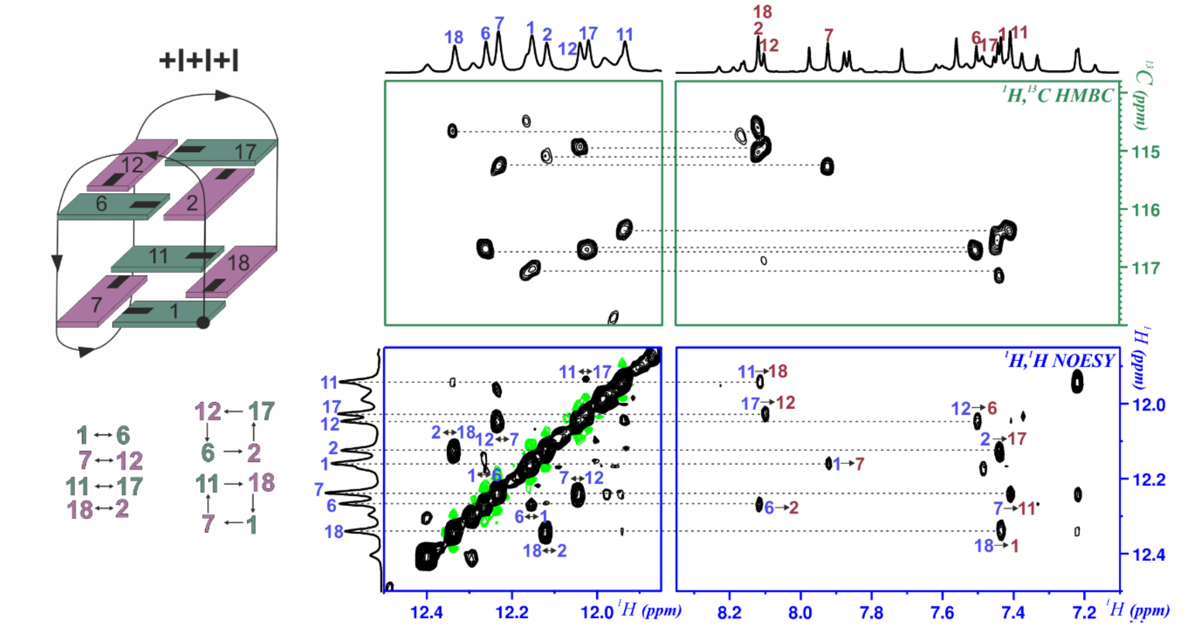

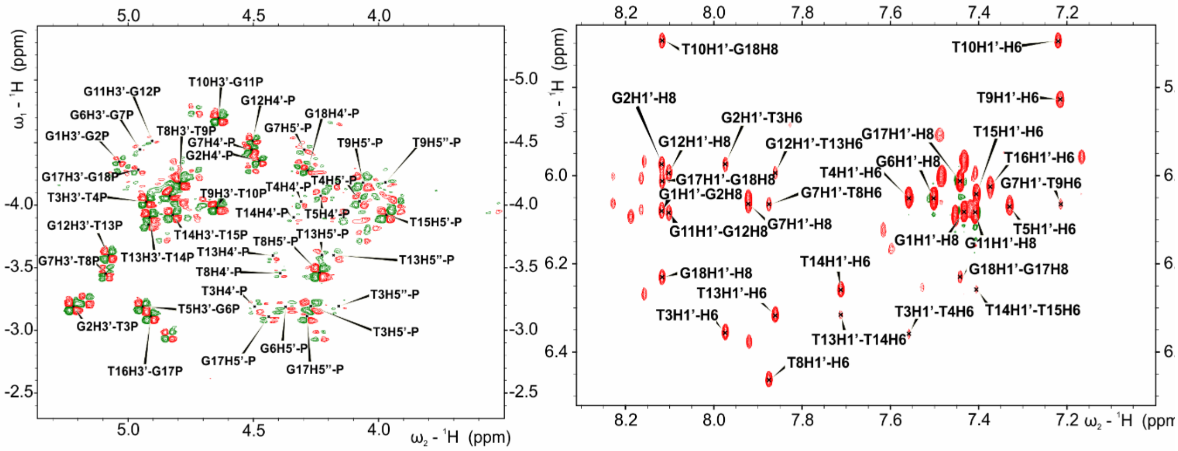

	H1'	H2'	H2''	H3'	H4'	H5'	H5''	H6/H8	Me	P	HN	C5
**G1**	6.081	2.738	2.989	5.034	4.416	3.979	3.979	7.434	-	-	12.15	117.1
**G2**	5.975	2.785	2.342	5.220	4.478	4.269	-	8.118	-	-4.353	12.12	115.1
**T3**	6.356	2.104	2.408	4.928	4.497	4.266	4.160	7.974	2.130	-3.193	-	-
**T4**	6.051	1.870	2.337	4.835	4.308	4.076	-	7.558	1.745	-4.029	-	-
**T5**	6.070	2.385	2.528	4.944	4.232	4.064	3.970	7.330	1.228	-4.076	-	-
**G6**	6.051	3.398	2.864	4.953	4.458	4.374	-	7.504	-	-3.189	12.26	116.7
**G7**	6.065	2.681	2.359	5.088	4.508	4.300	4.244	7.922	-	-4.444	12.23	115.3
**T8**	6.462	2.527	2.628	4.795	4.392	4.236	-	7.875	1.978	-3.458	-	-
**T9**	5.827	-	2.100	4.646	3.757	4.064	3.969	7.214	1.812	-4.181	-	-
**T10**	5.695	2.021	2.519	4.640	3.929	3.545	2.948	7.219	1.781	-3.982	-	-
**G11**	6.083	3.716	2.919	4.902	4.453	4.326	4.176	7.408	-	-4.696	11.94	116.4
**G12**	5.994	2.577	2.390	5.077	4.503	4.312	4.253	8.102	-	-4.516	12.04	114.9
**T13**	6.316	2.319	2.506	4.914	4.425	4.226	4.179	7.861	2.029	-3.600	-	-
**T14**	6.259	2.240	2.390	4.824	4.340	4.128	-	7.712	1.912	-3.903	-	-
**T15**	6.042	1.965	2.388	4.793	4.201	3.971	-	7.405	1.649	-3.923	-	-
**T16**	6.025	2.379	2.508	4.909	4.184	4.023	3.982	7.374	1.317	-	-	-
**G17**	6.012	3.400	2.853	4.958	4.454	4.438	4.285	7.442	-	-3.113	12.02	116.6
**G18**	6.230	2.696	2.477	4.805	4.284	4.248	4.195	8.117	-	-4.281	12.34	114.7

VII

G_1G_2**TTTT**G_7G_8**TTT**$G_{12}G_{13}$**TT**$G_{16}G_{17}$ **(432)**

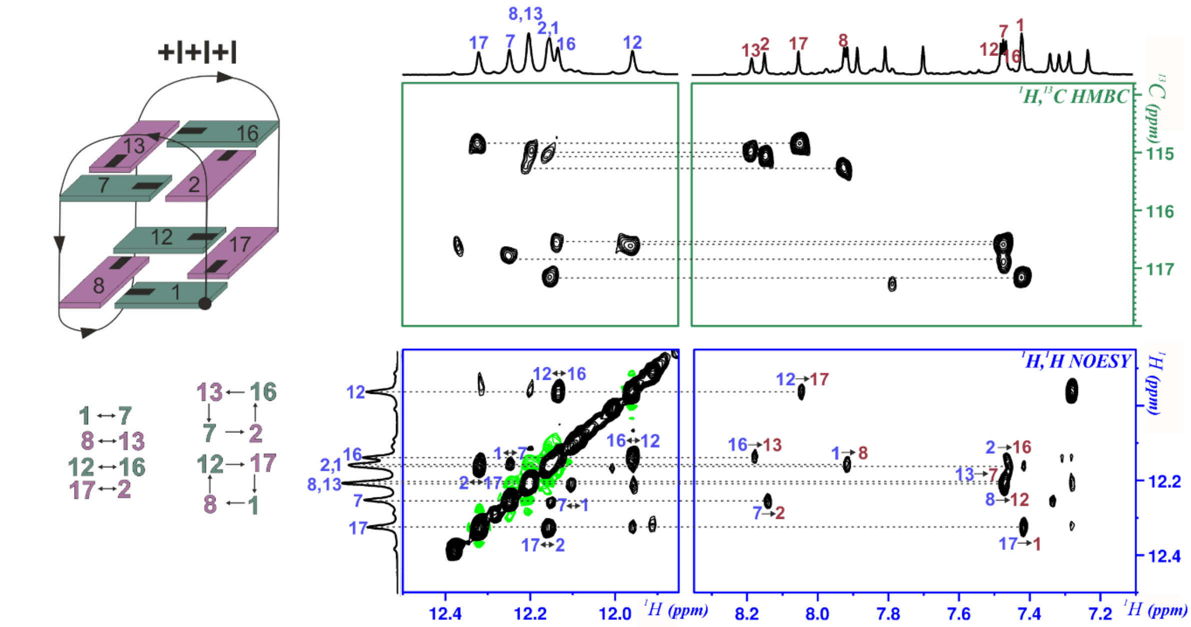

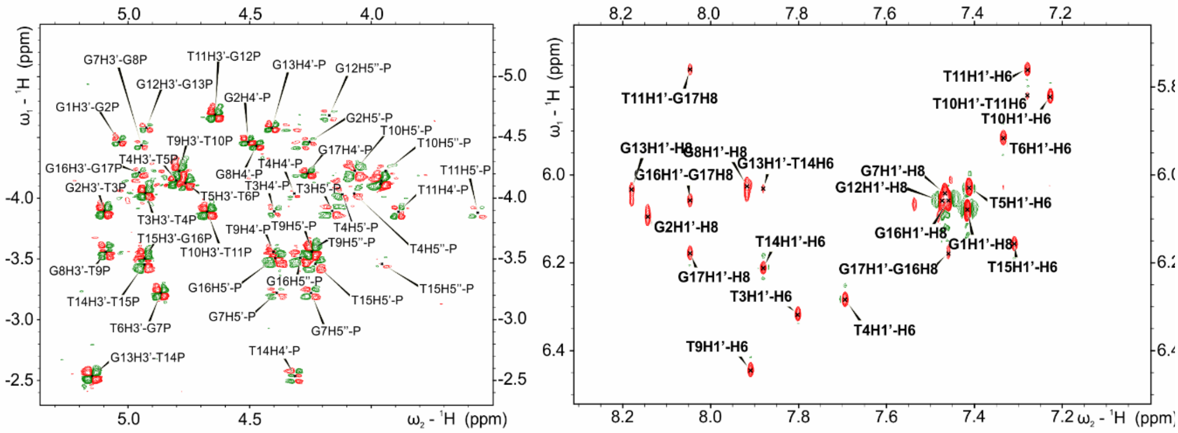

	H1'	H2'	H2''	H3'	H4'	H5'	H5''	H6/H8	Me	P	HN	C5
**G1**	6.079	2.773	2.975	5.036	4.407	3.974	3.974	7.416	-	-	12.15	117.2
**G2**	6.099	2.667	2.421	5.096	4.506	4.253	-	8.143	-	-4.465	12.16	115.1
**T3**	6.317	2.337	2.515	4.927	4.399	4.160	-	7.802	2.020	-3.895	-	-
**T4**	6.284	2.292	2.428	4.800	4.319	4.119	4.069	7.694	1.909	-4.038	-	-
**T5**	6.029	2.102	2.381	4.763	4.185	3.953	-	7.412	1.682	-4.146	-	-
**T6**	5.916	2.317	2.478	4.866	4.105	3.972	-	7.334	1.316	-4.125	-	-
**G7**	6.042	3.419	2.848	4.945	-	4.388	4.247	7.467	-	-3.221	12.25	116.8
**G8**	6.026	2.693	2.349	5.090	4.472	4.282	4.229	7.918	-	-4.434	12.21	115.3
**T9**	6.444	2.444	2.606	4.789	4.405	4.242	4.242	7.909	1.977	-3.561	-	-
**T10**	5.821	-	2.136	4.672	3.790	4.066	3.934	7.228	1.815	-4.225	-	-
**T11**	5.760	1.988	2.481	4.640	3.890	3.562	3.014	7.279	1.784	-3.886	-	-
**G12**	6.059	3.702	2.946	4.925	-	-	4.172	7.473	-	-4.685	11.96	116.6
**G13**	6.032	2.950	2.360	5.148	4.408	4.314	4.287	8.180	-	-4.582	12.19	115.0
**T14**	6.211	2.208	2.573	4.932	4.312	-	-	7.880	1.992	-2.536	-	-
**T15**	6.156	2.136	2.743	4.930	4.314	4.228	3.954	7.309	1.010	-3.458	-	-
**G16**	6.058	3.522	2.961	4.951	-	4.393	4.290	7.461	-	-3.506	12.13	116.6
**G17**	6.178	2.680	2.463	4.808	4.260	4.288	4.202	8.047	-	-4.210	12.32	114.8

VIII

G_1G_2**TTTT**G_7G_8**TTT**$G_{12}G_{13}$**TTT**$G_{17}G_{18}$ **(433)**

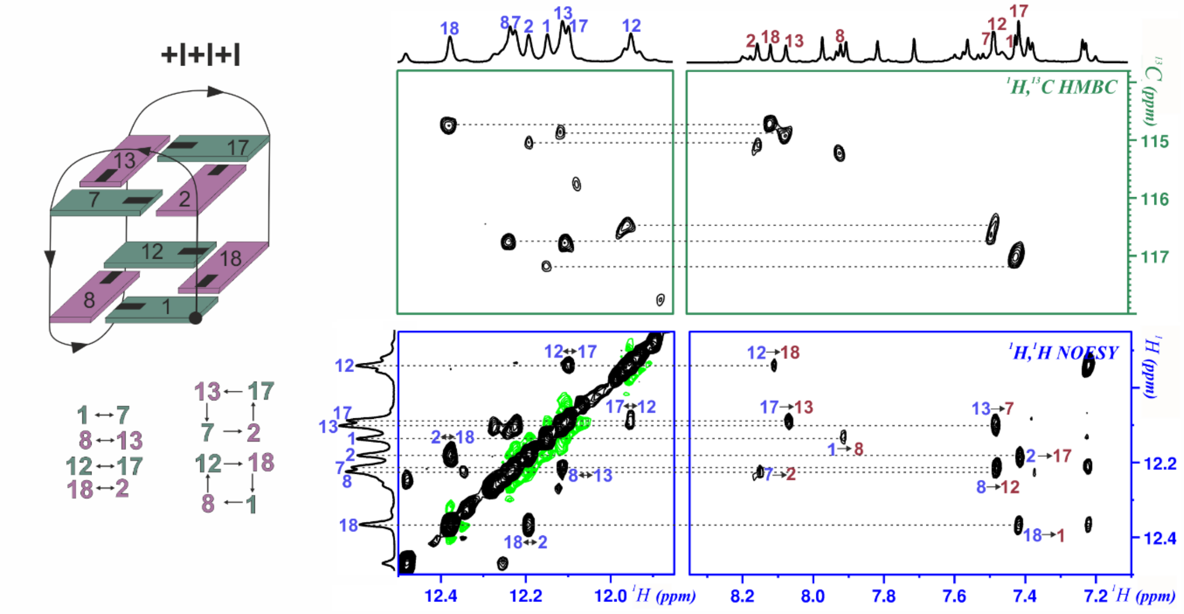

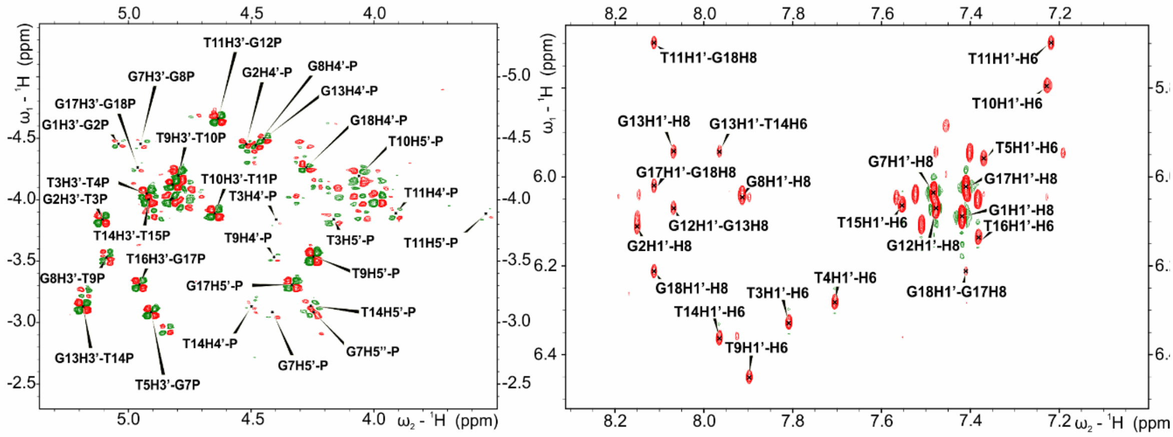

	H1'	H2'	H2''	H3'	H4'	H5'	H5''	H6/H8	Me	P	HN	C5
**G1**	6.088	2.742	2.992	5.038	4.412	3.976	3.976	7.418	-	-	12.14	117.1
**G2**	6.113	2.680	2.410	5.106	4.519	4.266	-	8.149	-	-4.455	12.19	115.1
**T3**	6.327	2.338	2.516	4.924	4.398	4.163	-	7.809	2.016	-3.840	-	-
**T4**	6.281	2.297	2.404	4.827	4.337	4.129	4.086	7.705	1.892	-4.050	-	-
**T5**	5.957	2.375	2.487	4.907	4.169	4.022	3.974	7.370	1.351	-	-	-
**T6**	6.038	1.990	2.406	4.819	4.206	3.984	-	7.408	1.653	-	-	-
**G7**	6.035	3.381	2.835	4.950	-	4.415	4.245	7.484	-	-3.085	12.23	116.6
**G8**	6.045	2.666	2.346	5.087	4.482	4.292	4.231	7.913	-	-4.449	12.22	115.2
**T9**	6.450	2.466	2.639	4.797	4.407	4.246	-	7.898	1.975	-3.533	-	-
**T10**	5.794	-	2.094	4.647	3.763	4.060	3.961	7.228	1.816	-4.196	-	-
**T11**	5.698	2.011	2.507	4.635	3.912	3.545	2.957	7.219	1.771	-3.889	-	-
**G12**	6.070	3.688	2.900	4.901	4.445	4.306	-	7.477	-	-4.661	11.95	116.4
**G13**	5.943	2.703	2.314	5.184	4.447	4.325	4.262	8.068	-	-4.497	12.11	114.9
**T14**	6.362	2.084	2.410	4.917	4.498	4.257	-	7.965	2.132	-3.134	-	-
**T15**	6.063	1.859	2.308	4.817	4.296	4.065	-	7.554	1.744	-4.003	-	-
**T16**	6.136	2.398	2.562	4.954	4.233	4.063	3.972	7.382	1.217	-	-	-
**G17**	6.021	3.458	2.869	4.961	4.454	4.333	-	7.411	-	-3.309	12.10	116.9
**G18**	6.212	2.698	2.466	4.804	4.277	-	-	8.112	-	-4.263	12.38	114.7

IX

G_1G_2**TTT**G_6G_7**TTTT**$G_{12}G_{13}$**TT**$G_{16}G_{17}$ **(342)**

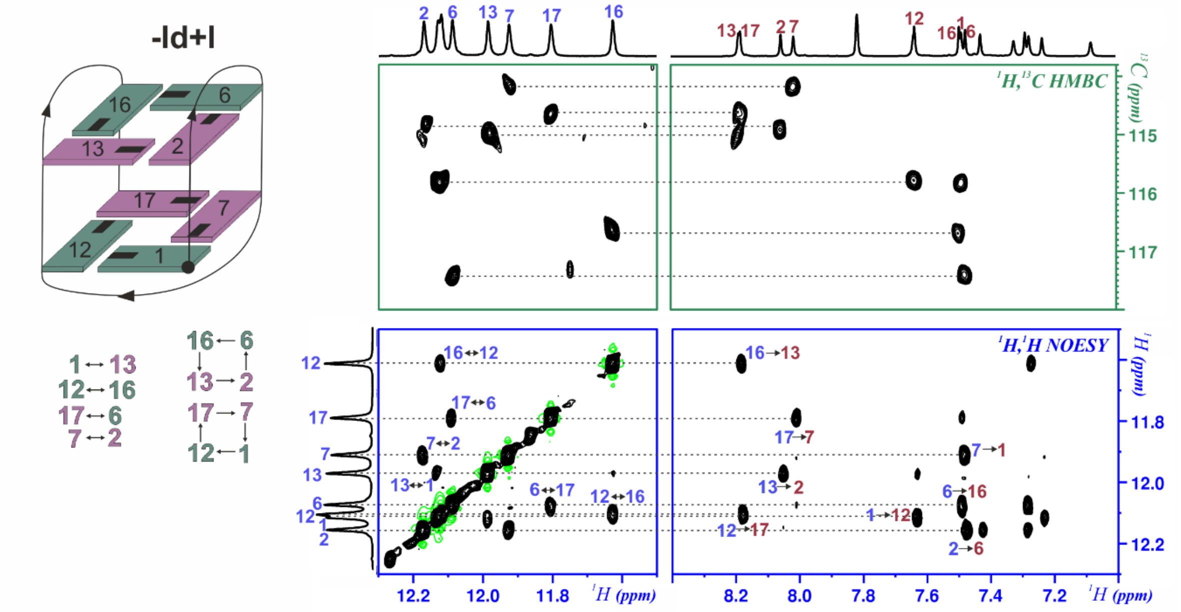

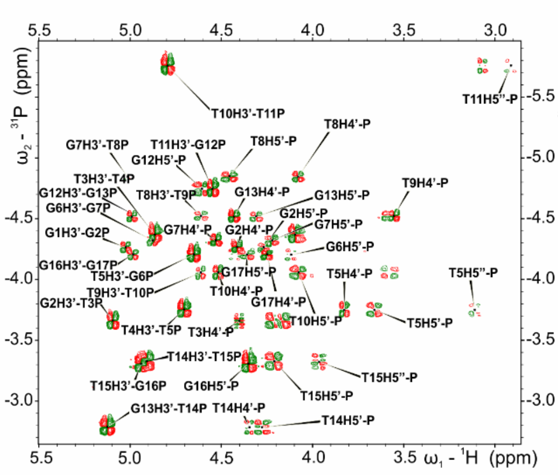

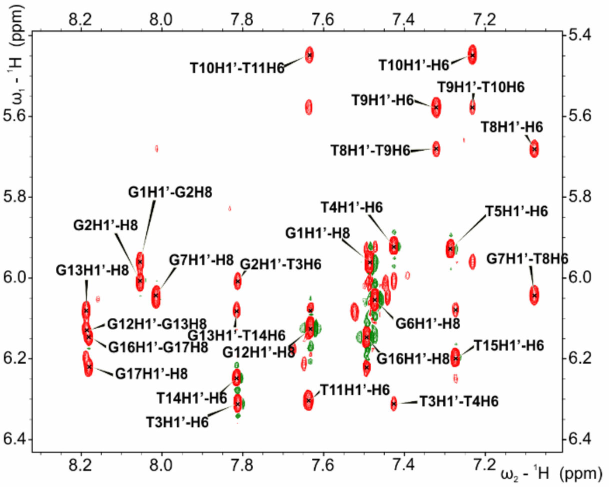

	H1'	H2'	H2''	H3'	H4'	H5'	H5''	H6/H8	Me	P	HN	C5
**G1**	5.961	2.940	3.023	5.024	4.389	4.034	3.939	7.485	-	-	12.13	115.8
**G2**	6.007	2.692	2.431	5.094	4.414	4.268	-	8.054	-	-4.266	12.17	114.9
**T3**	6.311	2.347	2.598	4.866	4.402	4.212	4.172	7.812	1.976	-3.660	-	-
**T4**	5.922	2.045	2.239	4.705	4.097	-	-	7.426	1.747	-4.369	-	-
**T5**	5.928	1.962	2.267	4.652	3.826	3.661	3.110	7.285	1.499	-3.751	-	-
**G6**	6.053	3.434	2.741	4.876	4.423	4.116	-	7.475	-	-4.205	12.09	117.4
**G7**	6.043	2.638	2.677	5.014	4.530	4.220	-	8.015	-	-4.321	11.93	114.2
**T8**	5.680	1.439	2.295	4.611	4.080	4.456	4.151	7.078	1.837	-4.846	-	-
**T9**	5.577	2.116	1.981	4.614	3.552	3.662	3.578	7.321	1.608	-4.523	-	-
**T10**	5.448	1.818	2.438	4.797	4.521	4.076	4.020	7.231	1.572	-4.057	9.01	-
**T11**	6.302	2.159	2.467	4.548	3.063	3.027	2.910	7.636	1.808	-5.758	-	-
**G12**	6.125	3.518	2.967	4.990	4.481	4.607	4.162	7.633	-	-4.747	12.12	115.8
**G13**	6.081	2.906	2.391	5.124	4.431	4.309	-	8.187	-	-4.517	11.98	115.0
**T14**	6.247	2.230	2.570	4.906	4.345	4.280	4.241	7.815	2.002	-2.786	-	-
**T15**	6.197	2.178	2.725	4.939	4.358	4.207	3.965	7.274	1.293	-3.326	-	-
**G16**	6.146	3.687	3.110	4.983	4.475	4.352	-	7.494	-	-3.306	11.63	116.7
**G17**	6.220	2.605	2.401	4.797	4.251	4.359	-	8.181	-	-4.206	11.80	114.6

X G$_1$G$_2$**TTT**G$_6$G$_7$**TTTT**G$_{12}$G$_{13}$**TTT**G$_{17}$G$_{18}$ **(343)**

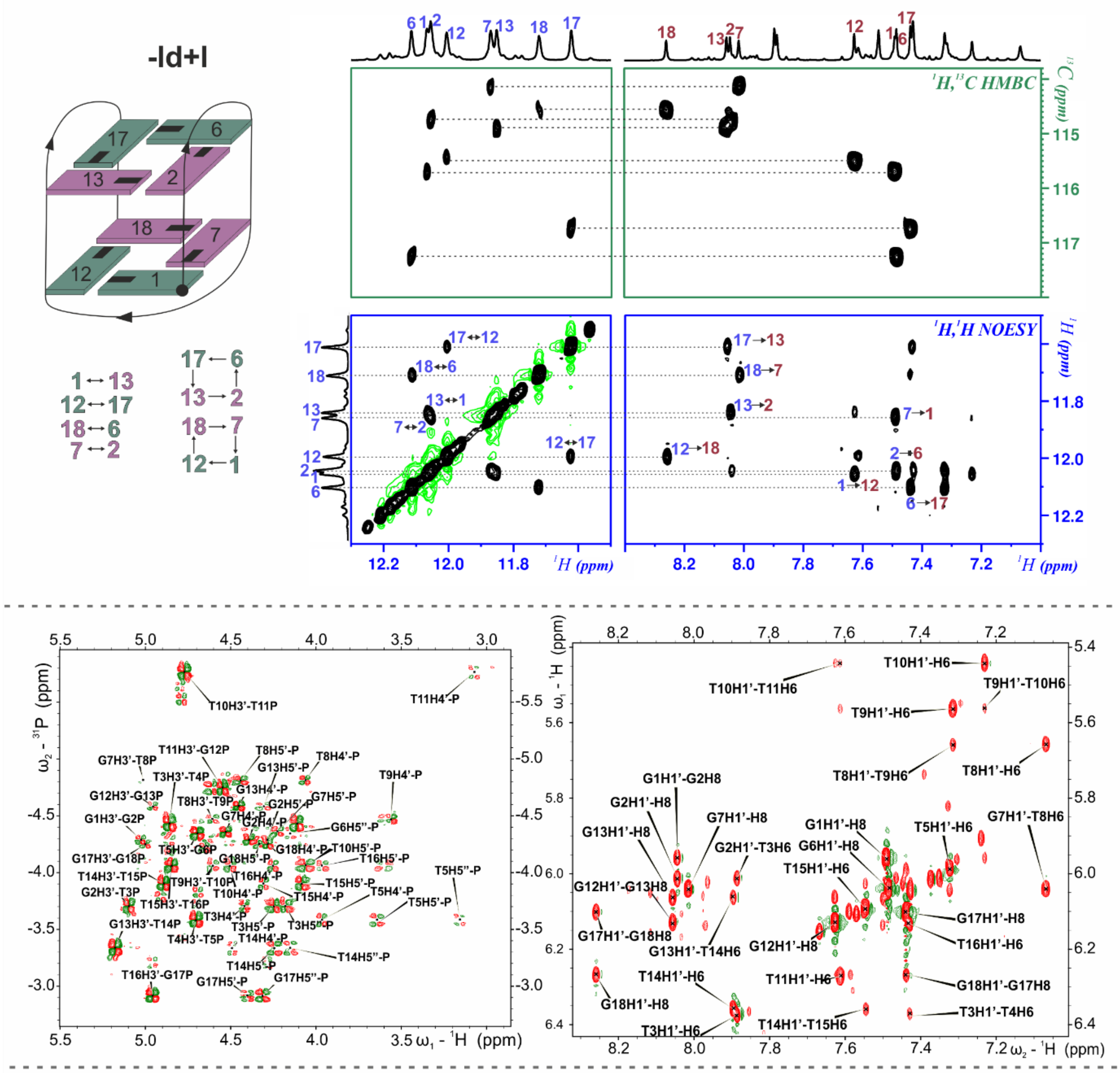

	H1'	H2'	H2"	H3'	H4'	H5'	H5"	H6/H8	Me	P	HN	C5
**G1**	5.959	2.931	3.045	5.025	4.395	4.028	3.944	7.492	-	-	12.07	115.7
**G2**	6.012	2.687	2.488	5.096	4.391	4.286	4.245	8.044	-	-4.289	12.06	114.8
**T3**	6.374	2.361	2.618	4.861	4.419	4.247	4.179	7.886	1.996	-3.704	-	-
**T4**	6.043	2.022	2.314	4.702	4.041	4.116	-	7.428	1.684	-4.434	-	-
**T5**	5.986	2.035	2.360	4.697	3.967	3.643	3.157	7.323	1.599	-3.585	-	-
**G6**	6.039	3.393	2.753	4.871	4.414	4.375	4.151	7.486	-	-4.345	12.11	117.3
**G7**	6.040	2.625	2.685	5.013	4.529	4.226	-	8.015	-	-4.361	11.87	114.1
**T8**	5.658	1.434	2.288	4.606	4.062	4.442	-	7.068	1.833	-4.814	-	-
**T9**	5.561	2.095	1.981	4.604	3.558	3.657	3.587	7.314	1.611	-4.471	-	-
**T10**	5.442	1.800	2.397	4.774	4.506	4.060	4.011	7.231	1.567	-4.061	-	-
**T11**	6.271	2.139	2.462	4.548	3.069	2.973	2.927	7.613	1.796	-5.765	-	-
**G12**	6.129	3.495	2.936	4.957	4.486	4.155	-	7.628	-	-4.748	12.00	115.5
**G13**	6.063	2.680	2.398	5.177	4.453	4.306	-	8.057	-	-4.583	11.85	114.9
**T14**	6.358	2.243	2.493	4.893	4.492	4.244	4.151	7.897	2.086	-3.337	-	-
**T15**	6.091	1.871	2.461	4.845	4.307	4.084	-	7.545	1.695	-3.903	-	-
**T16**	6.132	2.430	2.557	4.957	4.252	3.969	-	7.431	1.448	-4.061	-	-
**G17**	6.101	3.591	3.027	5.000	4.473	4.405	4.313	7.439	-	-2.917	11.62	116.7
**G18**	6.267	2.657	2.420	4.803	4.283	4.332	4.222	8.259	-	-4.250	11.72	114.5

XI

G₁G₂TTTG₆G₇TTTTG₁₂G₁₃TTTTG₁₈G₁₉ (344)

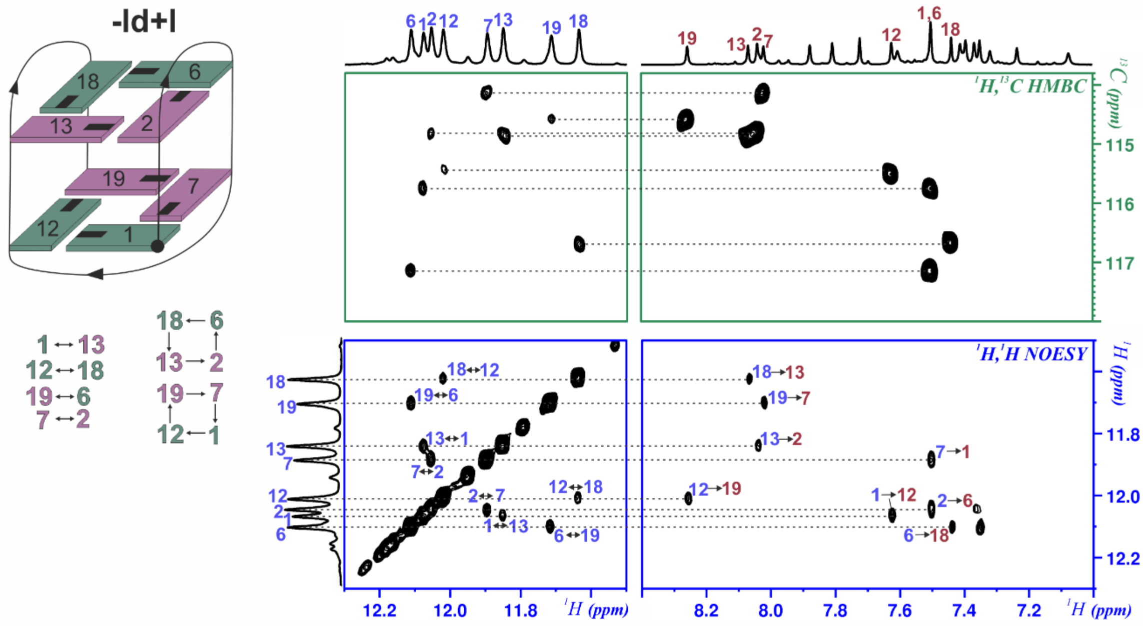

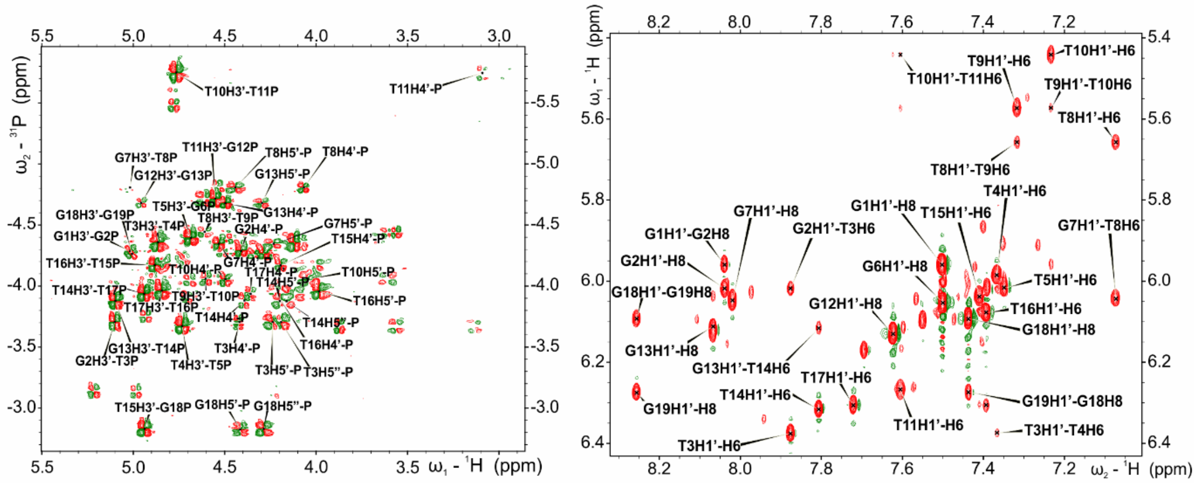

	H1'	H2'	H2''	H3'	H4'	H5'	H5''	H6/H8	P	HN	C5
G1	5.958	2.930	3.035	5.016	4.391	4.026	3.939	7.503	-	12.08	115.7
G2	6.017	2.696	2.462	5.098	4.408	4.290	4.250	8.039	-4.287	12.05	114.8
T3	6.374	2.375	2.619	4.864	4.419	4.238	4.192	7.876	-3.705	-	-
T4	5.983	2.008	2.328	4.730	4.075	4.117	-	7.367	-4.359	-	-
T5	6.014	2.039	2.373	4.685	3.873	3.561	3.132	7.349	-3.672	-	-
G6	6.052	3.418	2.763	4.875	4.421	4.366	4.149	7.501	-4.395	12.11	117.1
G7	6.043	2.630	2.688	5.017	4.531	4.233	-	8.020	-4.347	11.89	114.1
T8	5.657	1.451	2.298	4.607	4.067	4.442	-	7.073	-4.807	-	-
T9	5.571	2.084	1.986	4.602	3.558	3.661	3.607	7.317	-4.443	-	-
T10	5.441	1.807	2.397	4.765	4.498	4.044	-	7.233	-4.058	-	-
T11	6.265	2.140	2.460	4.549	3.091	2.973	2.935	7.605	-5.747	-	-
G12	6.128	3.506	2.929	4.954	4.486	4.152	-	7.624	-4.715	12.01	115.5
G13	6.115	2.572	2.368	5.096	4.496	4.296	-	8.068	-4.680	11.84	114.9
T14	6.315	2.314	2.500	4.939	4.392	4.198	4.162	7.806	-3.878	-	-
T15	6.037	2.433	2.525	4.937	4.193	-	-	7.410	-4.170	-	-
T16	6.077	2.009	2.429	4.880	4.223	3.984	-	7.393	-3.959	-	-
T17	6.304	2.299	2.453	4.842	4.358	-	-	7.721	-3.936	-	-
G18	6.091	3.562	3.010	5.004	4.475	4.417	4.285	7.438	-2.828	11.63	116.7
G19	6.273	2.658	2.426	4.802	4.284	4.333	4.222	8.258	-4.264	11.71	114.6

XII

G_1G_2**TTTT**G_7G_8**TTTT**$G_{13}G_{14}$**TT**$G_{17}G_{18}$ **(442)**

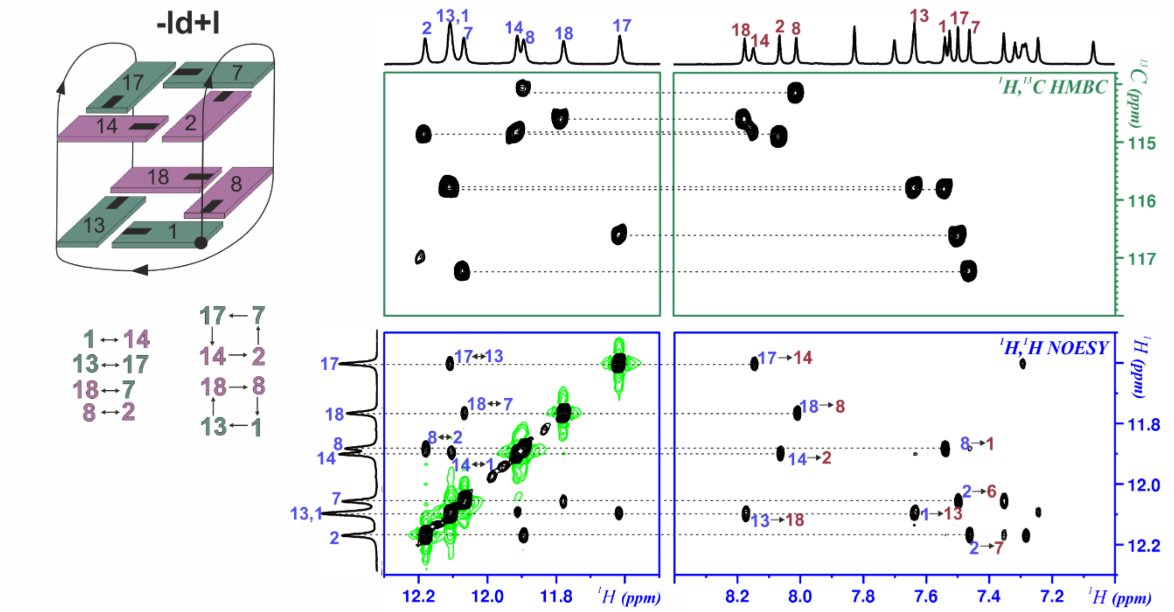

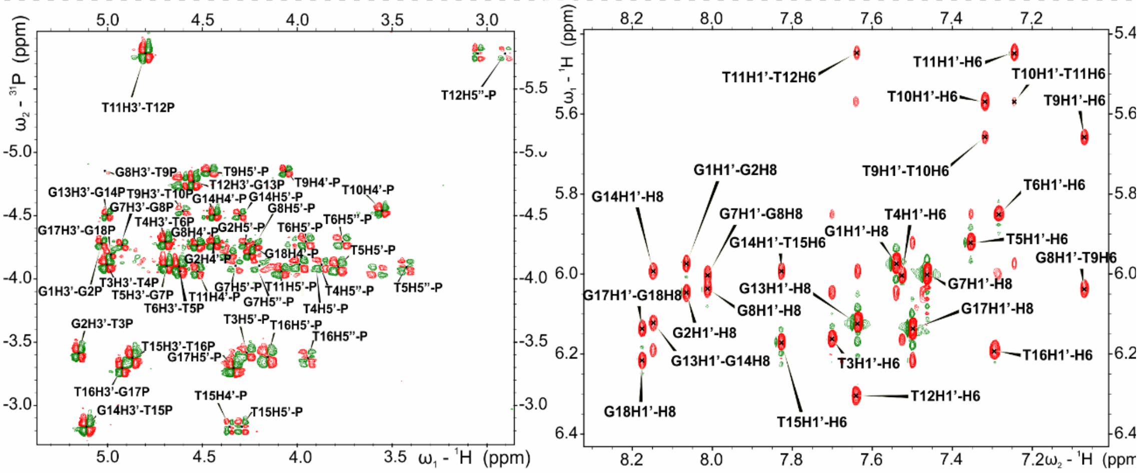

	H1'	H2'	H2''	H3'	H4'	H5'	H5''	H6/H8	Me	P	HN	C5
**G1**	5.975	2.954	3.059	5.032	4.391	4.039	3.941	7.540	-	-	12.11	115.8
**G2**	6.046	2.644	2.724	5.152	4.439	4.269	-	8.065	-	-4.279	12.19	114.9
**T3**	6.162	2.298	2.435	5.004	4.252	4.266	4.159	7.701	2.003	-3.414	-	-
**T4**	6.004	2.153	2.414	4.694	4.171	3.985	3.871	7.526	1.815	-4.113	-	-
**T5**	5.922	2.080	2.399	4.690	4.013	3.787	3.424	7.354	1.598	-4.096	-	-
**T6**	5.851	2.180	2.335	4.624	4.031	3.955	3.763	7.284	1.630	-4.293	-	-
**G7**	6.002	3.364	2.739	4.922	4.390	4.326	4.204	7.463	-	-4.104	12.07	117.2
**G8**	6.038	2.632	2.678	5.014	4.527	4.222	-	8.012	-	-4.266	11.91	114.1
**T9**	5.658	1.422	2.274	4.605	4.060	4.463	4.143	7.070	1.838	-4.851	-	-
**T10**	5.569	2.121	1.975	4.614	3.553	3.657	3.560	7.318	1.604	-4.540	-	-
**T11**	5.447	1.811	2.436	4.803	4.529	4.084	4.028	7.245	1.575	-4.061	-	-
**T12**	6.304	2.157	2.468	4.547	3.045	3.026	2.903	7.640	1.808	-5.783	-	-
**G13**	6.123	3.490	2.949	5.002	4.481	4.164	-	7.636	-	-4.768	12.12	115.8
**G14**	5.993	2.802	2.374	5.109	4.438	4.304	-	8.148	-	-4.506	11.93	114.8
**T15**	6.172	2.052	2.471	4.869	4.357	4.291	4.226	7.827	2.027	-2.832	-	-
**T16**	6.193	2.205	2.691	4.922	4.327	4.154	3.945	7.295	1.309	-3.377	-	-
**G17**	6.137	3.697	3.110	4.983	4.466	4.335	-	7.499	-	-3.295	11.63	116.6
**G18**	6.217	2.598	2.398	4.792	4.252	-	-	8.176	-	-4.194	11.79	114.6

XIII

G₁G₂**TTTT**G₇G₈**TTTT**G₁₃G₁₄**TTT**G₁₈G₁₉ **(443)**

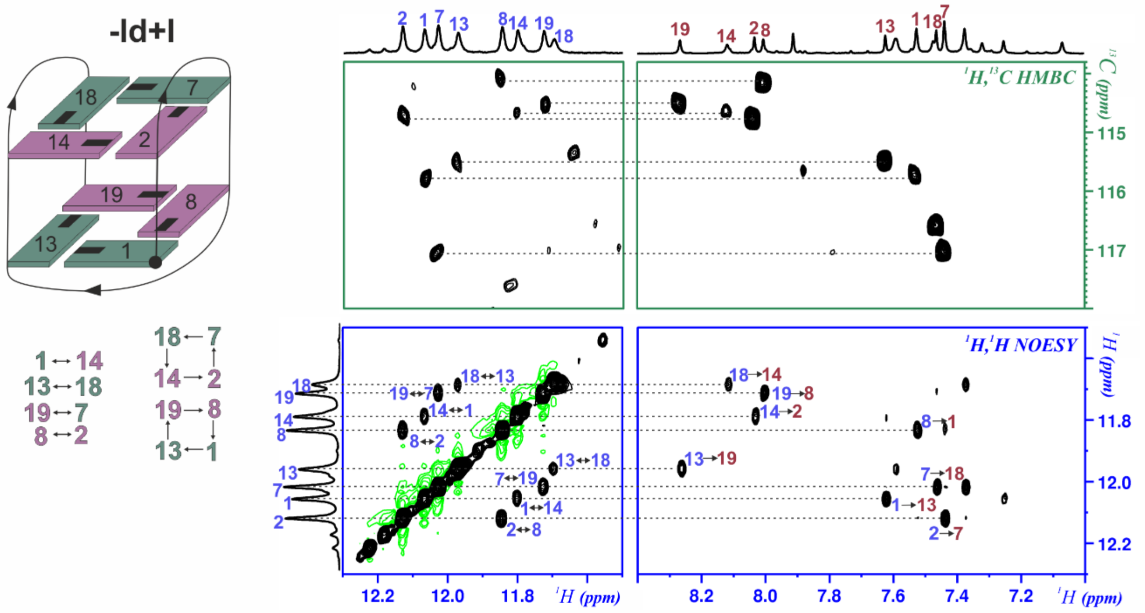

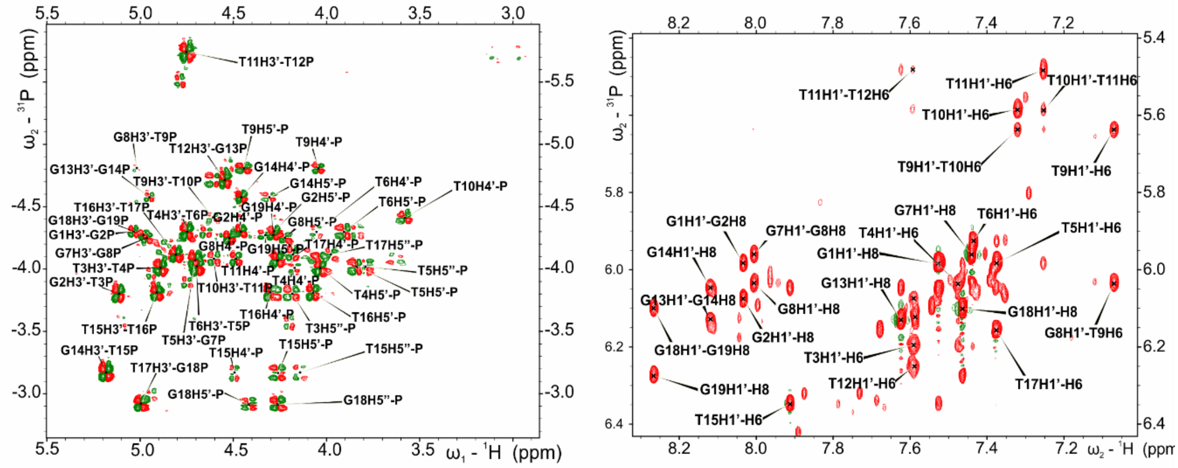

	H1'	H2'	H2''	H3'	H4'	H5'	H5''	H6/H8	Me	P	HN	C5
**G1**	5.982	2.921	3.052	5.033	4.405	4.048	3.951	7.527	-	-	12.06	115.8
**G2**	6.075	2.642	2.626	5.113	4.460	4.281	-	8.034	-	-4.301	12.13	114.7
**T3**	6.196	2.287	2.449	4.888	4.308	4.296	4.185	7.591	1.904	-3.802	-	-
**T4**	6.038	2.112	2.306	4.749	4.171	4.033	-	7.475	1.788	-4.009	-	-
**T5**	5.982	2.284	2.546	4.704	4.180	3.838	3.791	7.375	1.650	-4.020	-	-
**T6**	5.927	2.064	2.332	4.695	4.056	3.907	-	7.437	1.625	-4.298	-	-
**G7**	5.960	3.314	2.744	4.963	-	-	-	7.439	-	-4.070	12.03	117.0
**G8**	6.036	2.619	2.685	5.014	4.521	4.223	-	8.006	-	-4.249	11.84	114.1
**T9**	5.638	1.462	2.272	4.607	4.043	4.443	-	7.070	1.834	-4.808	-	-
**T10**	5.585	2.066	1.991	4.603	3.577	3.667	3.583	7.321	1.614	-4.412	-	-
**T11**	5.483	1.830	2.382	4.751	4.490	4.052	4.007	7.254	1.569	-4.083	-	-
**T12**	6.249	2.130	2.461	4.545	-	-	-	7.591	1.799	-5.740	-	-
**G13**	6.130	3.489	2.920	4.951	-	-	-	7.623	-	-4.713	11.97	115.5
**G14**	6.047	2.687	2.421	5.183	4.457	4.311	4.267	8.119	-	-4.576	11.79	114.6
**T15**	6.348	2.243	2.428	4.910	4.495	4.258	4.141	7.912	2.113	-3.169	-	-
**T16**	6.047	1.820	2.371	4.809	4.306	4.074	-	7.525	1.691	-3.811	-	-
**T17**	6.157	2.479	2.525	4.995	4.181	4.084	3.976	7.376	1.479	-4.116	-	-
**G18**	6.100	3.604	3.021	4.982	4.467	4.418	4.262	7.464	-	-2.917	11.69	116.6
**G19**	6.274	2.658	2.428	4.800	4.286	4.328	4.220	8.267	-	-4.273	11.72	114.5

XIV

G_1G_2**TTTT**G_7G_8**T**$G_{10}G_{11}$**TTTT**$G_{16}G_{17}$ **(414)**

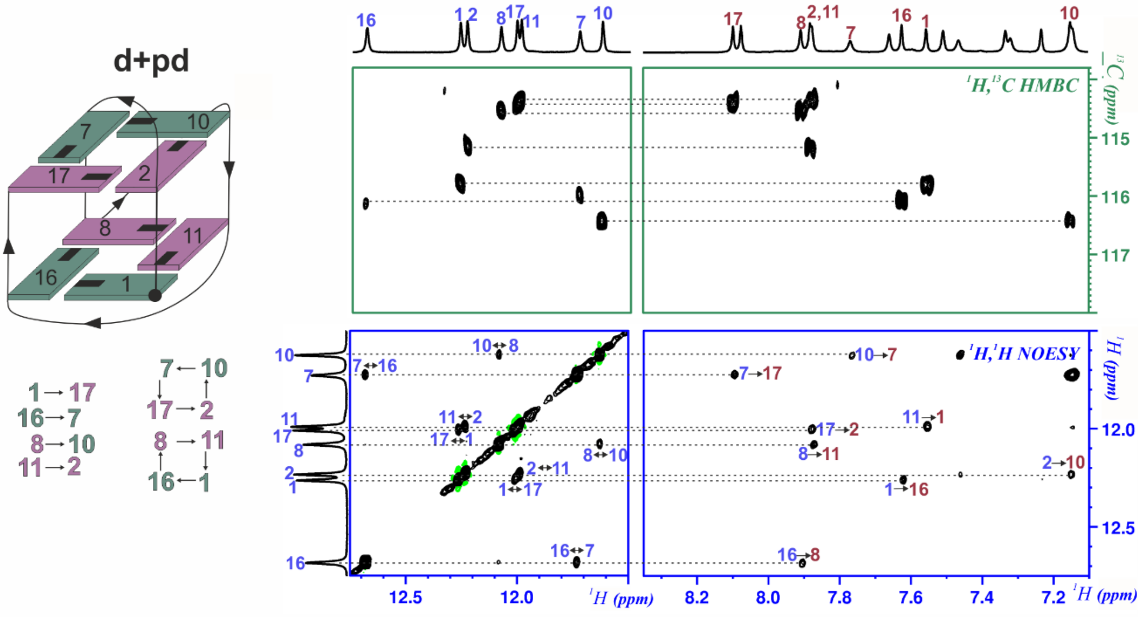

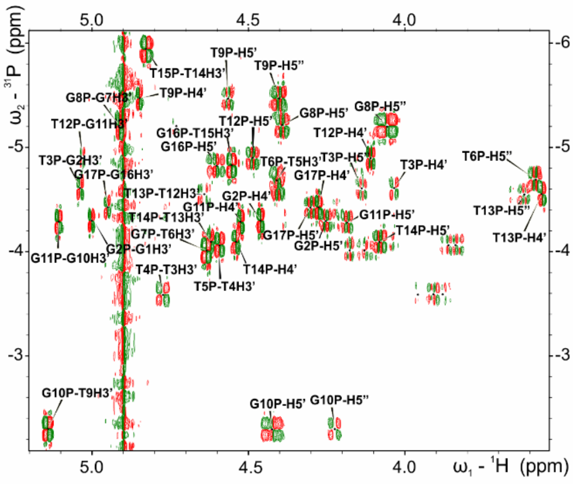

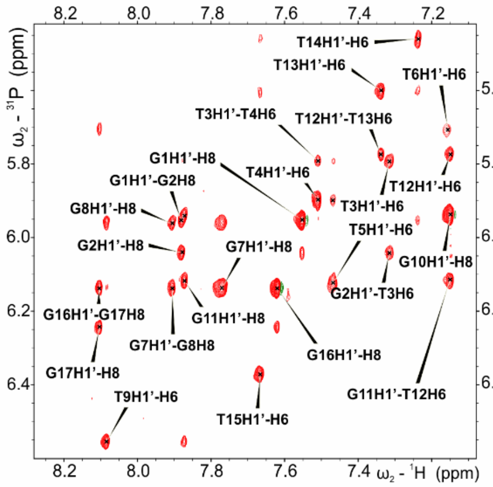

	H1'	H2'	H2''	H3'	H4'	H5'	H5''	H6/H8	Me	P	HN	C5
**G1**	5.955	2.850	2.980	5.000	4.385	4.028	3.932	7.552	-	-	12.27	115.8
**G2**	6.041	2.531	2.665	5.037	4.460	4.253	-	7.879	-	-4.297	12.24	115.1
**T3**	5.793	2.109	2.299	4.772	4.033	4.402	4.136	7.315	1.877	-4.609		-
**T4**	5.900	1.790	2.257	4.590	3.954	3.904	3.869	7.509	1.810	-3.587		-
**T5**	6.125	2.146	2.180	4.407	4.120	3.859	3.837	7.468	1.525	-4.062		-
**T6**	5.709	1.923	2.453	4.631	-	-	3.582	7.157	1.807	-4.688		-
**G7**	6.138	3.745	3.266	4.910	4.457	4.176	4.128	7.770	-	-4.009	11.73	116.0
**G8**	5.961	2.221	2.726	4.900	4.731	4.393	4.057	7.905	-	-5.208	12.08	114.5
**T9**	6.556	2.761	2.636	5.141	4.847	4.565	4.402	8.084	1.929	-5.470		-
**G10**	5.940	2.585	2.676	5.106	4.459	4.424	4.225	7.149	-	-2.299	11.63	116.4
**G11**	6.117	2.742	2.675	5.029	4.524	4.184	-	7.872	-	-4.284	11.99	114.4
**T12**	5.775	1.473	2.372	4.638	4.110	4.487	4.168	7.149	1.881	-4.898		-
**T13**	5.602	2.134	1.997	4.620	3.560	3.666	3.617	7.338	1.628	-4.550		-
**T14**	5.461	1.845	2.442	4.825	4.540	4.080	4.037	7.236	1.569	-4.094		-
**T15**	6.372	2.184	2.463	4.551	3.056	3.042	2.882	7.667	1.800	-5.943		-
**G16**	6.139	3.608	2.966	4.949	4.490	4.597	4.147	7.620	-	-4.826	12.69	116.1
**G17**	6.245	2.647	2.464	4.789	4.276	4.298	4.215	8.103	-	-4.425	12.01	114.4

XV

G_1G_2**TTTT**G_7G_8**TT**$G_{11}G_{12}$**TTTT**$G_{17}G_{18}$ **(424)**

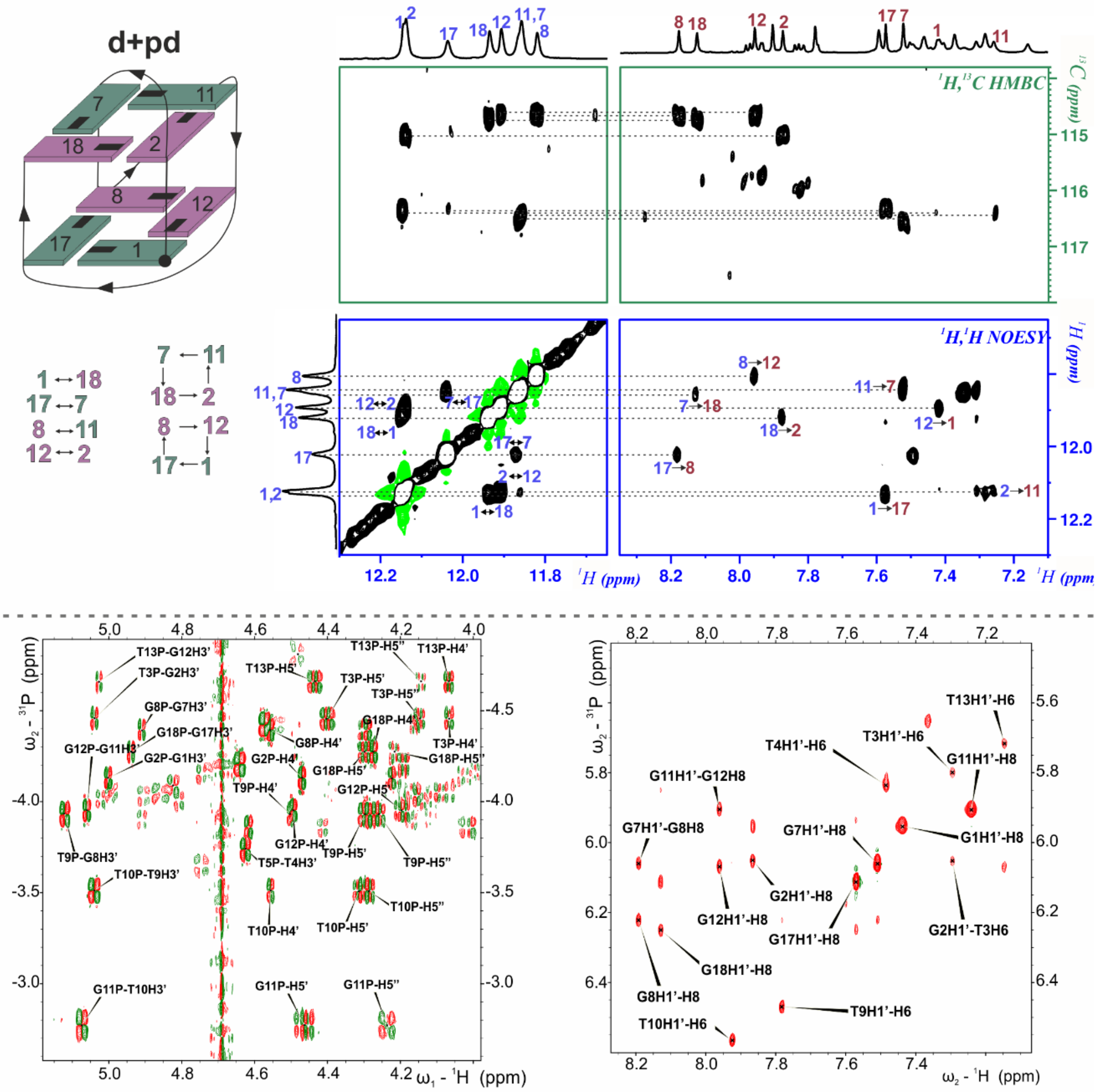

	H1'	H2'	H2''	H3'	H4'	H5'	H5''	H6/H8	P	HN	C5
**G1**	5.953	2.815	2.941	5.002	4.399	4.042	3.932	7.438	-	12.14	116.3
**G2**	6.051	2.546	2.659	5.040	4.470	-	-	7.870	-4.131	12.13	115.0
**T3**	5.799	2.001	2.342	4.745	4.066	4.402	4.148	7.295	-4.457	-	-
**T4**	5.838	-	-	4.625	3.859	3.884	-	7.485	-	-	-
**T5**	-	-	-	4.480	-	-	-	7.339	-3.740	-	-
**G7**	6.061	3.620	3.019	4.909	-	-	-	7.519	-	11.86	116.5
**G8**	6.221	2.648	2.531	5.121	4.556	4.289	-	8.188	-4.393	11.82	114.7
**T9**	6.470	2.458	2.621	5.041	4.502	4.297	4.259	7.783	-3.932	-	-
**T10**	6.565	2.553	2.697	5.072	4.556	4.314	4.286	7.924	-3.510	-	-
**G11**	5.906	3.049	2.781	5.061	-	4.464	4.237	7.251	-2.772	11.85	116.4
**G12**	6.070	2.636	2.659	5.027	4.494	4.199	-	7.960	-3.952	11.91	114.7
**T13**	5.718	1.643	2.319	4.650	4.067	4.435	4.143	7.146	-4.660	-	-
**G17**	6.112	3.558	2.965	4.938	-	-	-	7.572	-	12.03	116.3
**G18**	6.250	2.643	2.454	4.792	4.274	4.299	4.216	8.128	-4.274	11.93	114.7

XVI

G$_1$G$_2$**TTTT**G$_7$G$_8$**TTT**G$_{12}$G$_{13}$**TTTT**G$_{18}$G$_{19}$ **(434)**

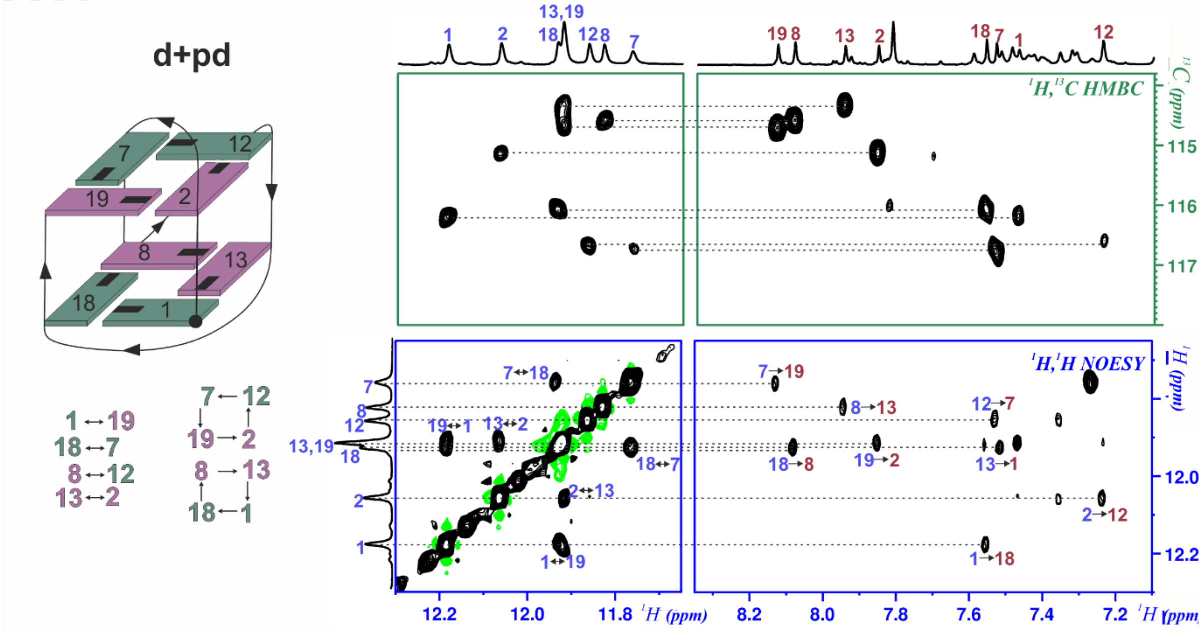

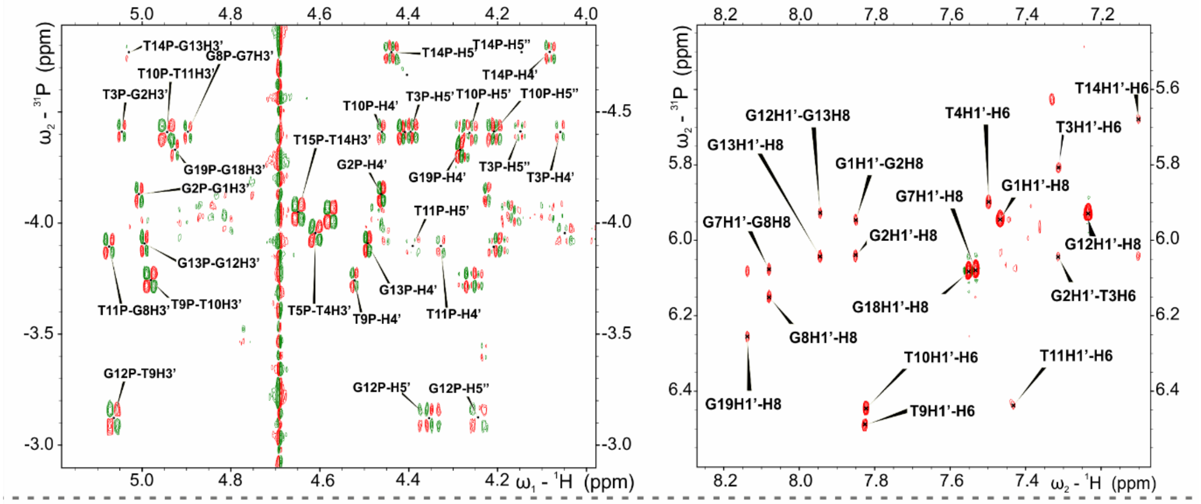

	H1'	H2'	H2"	H3'	H4'	H5'	H5"	H6/H8	P	HN	C5
G1	5.945	2.891	2.956	5.007	4.385	4.027	3.923	7.469	-	12.18	116.2
G2	6.041	2.547	2.666	5.047	4.462	-	-	7.851	-4.130	12.05	115.1
T3	5.806	2.059	2.324	4.765	4.060	4.396	4.150	7.313	-4.411	-	-
T4	5.898	-	-	4.608	3.965	3.897	3.890	7.498	-	-	-
T5	-	-	-	4.406	-	-	-	7.363	-3.954	-	-
G7	6.077	3.594	2.998	4.897	-	-	-	7.531	-	11.76	116.7
G8	6.151	2.625	2.695	5.075	4.408	4.272	4.226	8.081	-4.411	11.82	114.6
T9	6.489	2.530	2.609	5.065	4.522	4.262	-	7.826	-3.742	-	-
T10	6.446	2.516	2.672	4.983	4.461	4.262	4.207	7.823	-4.410	-	-
T11	6.436	2.242	2.551	4.946	4.328	4.393	4.210	7.434	-3.896	-	-
G12	5.927	3.077	2.781	4.995	4.419	4.356	4.246	7.236	-3.123	11.86	116.7
G13	6.043	2.656	-	5.031	4.493	-	-	7.944	-3.909	11.92	114.3
T14	5.677	1.621	2.327	4.647	4.081	4.439	4.146	7.101	-4.769	-	-
T15	-	-	-	-	-	-	-	-	-4.050	-	-
G18	6.083	3.542	2.895	4.925	-	-	-	7.557	-	11.93	116.1
G19	6.257	2.668	2.459	4.790	4.285	4.279	4.205	8.134	-4.330	11.91	114.7

XVII

G$_1$G$_2$**TTT**G$_6$G$_7$**TT**G$_{10}$G$_{11}$**TTT**G$_{15}$G$_{16}$ **(323)**

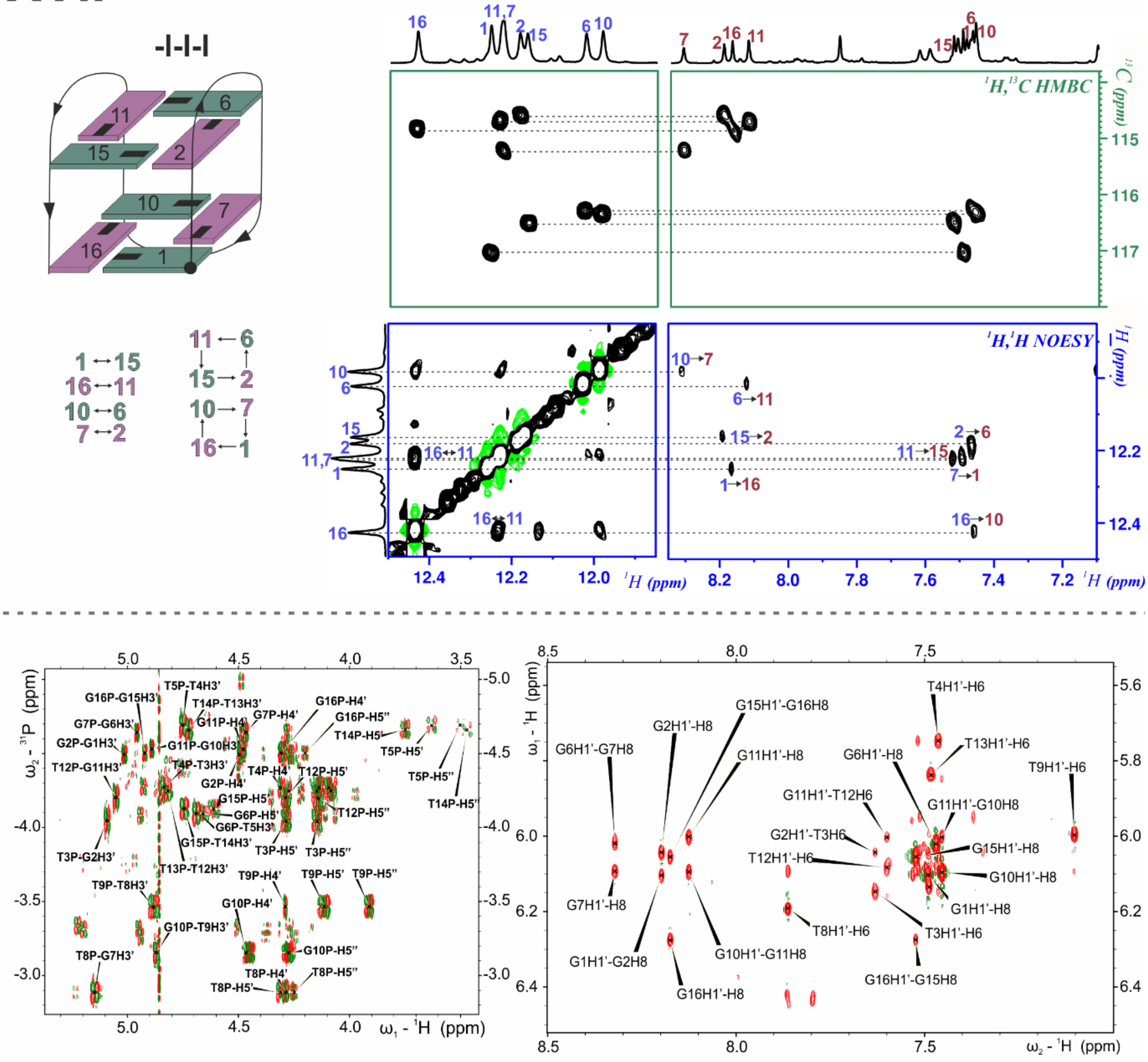

	H1'	H2'	H2''	H3'	H4'	H5'	H5''	H6/ H8	Me	P	HN	C5
**G1**	6.101	2.807	2.966	5.014	4.387	3.956	3.913	7.495	-	-	12.26	117.0
**G2**	6.043	2.679	2.486	5.091	4.497	4.265	4.234	8.197	-	-4.493	12.19	114.6
**T3**	6.147	2.299	2.482	4.835	4.346	4.285	4.144	7.631	1.852	-4.044	-	-
**T4**	5.747	1.895	2.302	4.751	4.312	4.137	4.093	7.464	1.829	-4.271	-	-
**T5**	6.052	2.191	2.436	4.690	3.766	3.628	3.500	7.518	1.731	-4.691	-	-
**G6**	6.018	3.375	2.842	4.958	4.429	4.659	4.111	7.468	-	-4.094	12.02	116.3
**G7**	6.092	3.013	2.455	5.147	4.466	4.282	-	8.317	-	-4.643	12.22	115.2
**T8**	6.193	2.128	2.490	4.882	4.286	4.310	4.248	7.863	1.995	-2.887	-	-
**T9**	5.996	2.051	2.614	4.871	4.288	4.110	3.912	7.103	1.159	-3.465	-	-
**G10**	6.093	3.394	2.934	4.889	4.466	4.456	4.273	7.457	-	-3.155	11.98	116.3
**G11**	6.002	2.597	2.513	5.053	4.477	4.296	4.234	8.125	-	-4.530	12.23	114.7
**T12**	6.083	2.229	2.477	4.818	4.353	4.286	4.140	7.600	1.827	-4.201	-	-
**T13**	5.837	1.922	2.276	4.725	4.273	4.148	4.083	7.482	1.810	-4.246	-	-
**T14**	6.133	2.256	2.487	4.745	3.848	3.748	3.472	7.489	1.718	-4.659	-	-
**G15**	6.054	3.422	2.854	4.922	4.457	4.611	4.133	7.523	-	-4.126	12.16	116.5
**G16**	6.274	2.698	2.396	4.774	4.307	4.294	4.200	8.172	-	-4.504	12.43	114.9

Supplementary Figure S9. Expected imino-mino and imino-aromatic NOEs for each of the 14 possible G4 topologies.

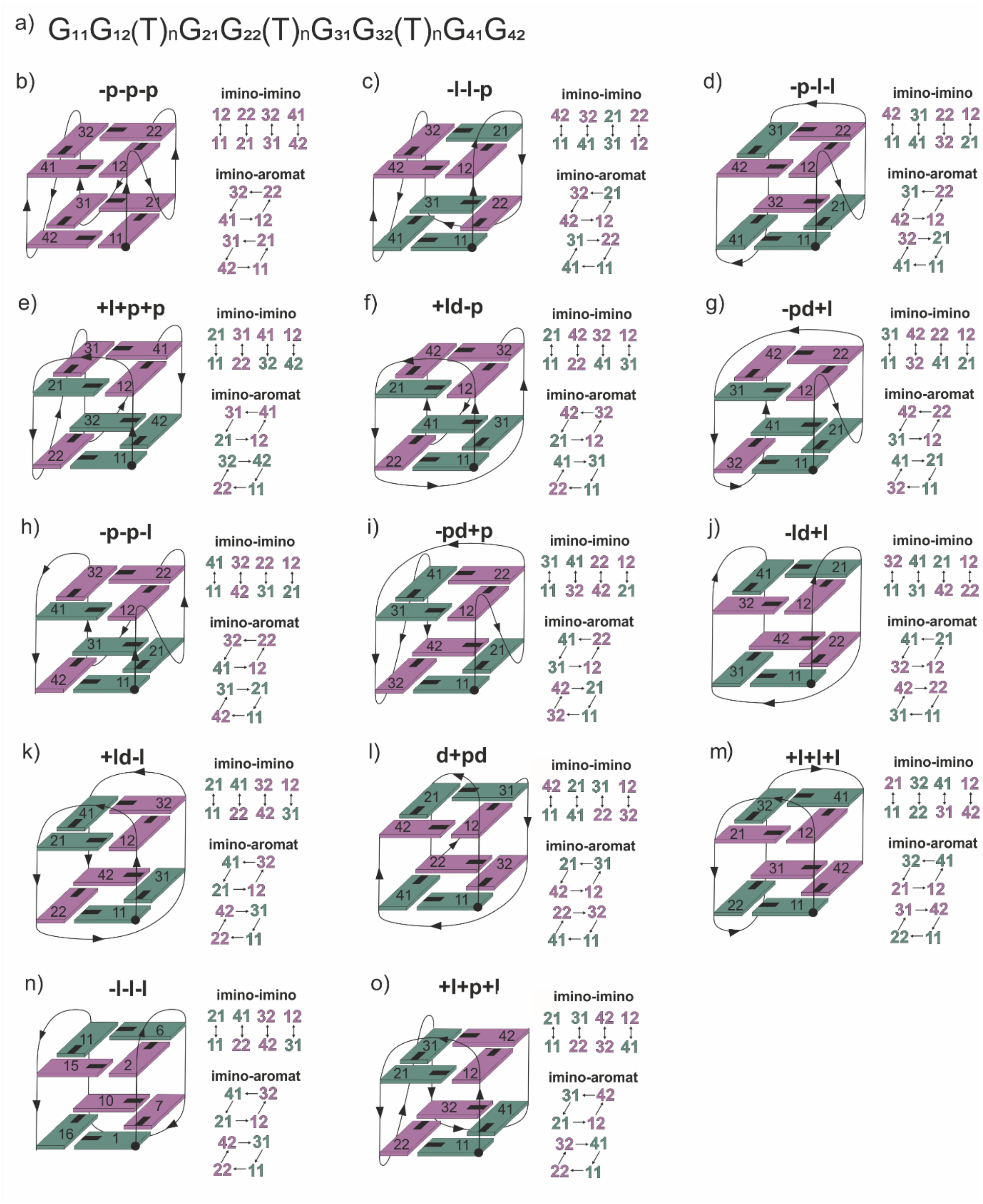

Supplementary Figure S10. Illustration of chemical shifts differences between **232** and TBA. Each assigned non-exchangeable proton and phosphorus atom is marked on the TBA structure (PDB entry 148D) by a sphere color coded according to the chemical shift difference between the two systems (from white = 0.00 ppm to red > 0.20 ppm). The residue G8 of TBA (T8 in **232**) is shown in yellow.

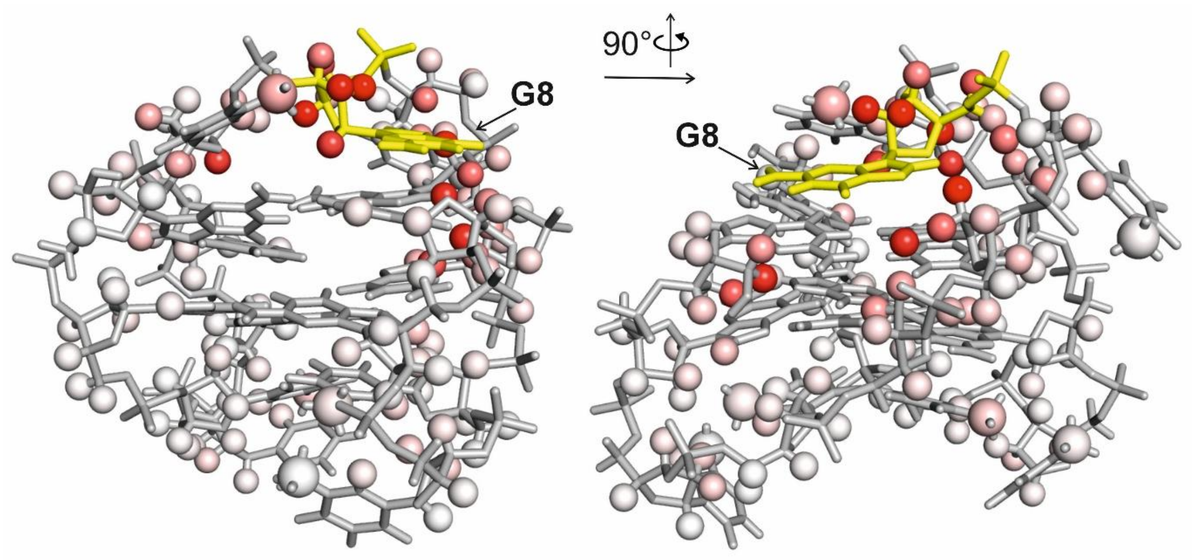

Supplementary Figure S11. The dominant G4 fold under NMR conditions as a function of the three loop lengths. For unimolecular G4s the assigned loop topology is indicated (if known; otherwise marked as unimolecular, “um”), while multimolecular G4s are grouped into a single category (“mm”, gray). Fields corresponding to sequences that produce comparable populations of uni- and multimolecular species are shown split in half into a gray and colored triangle. Systems for which loop topology assignment is tentative – **324** and **423** – are shown in a lighter shade of the appropriate color.

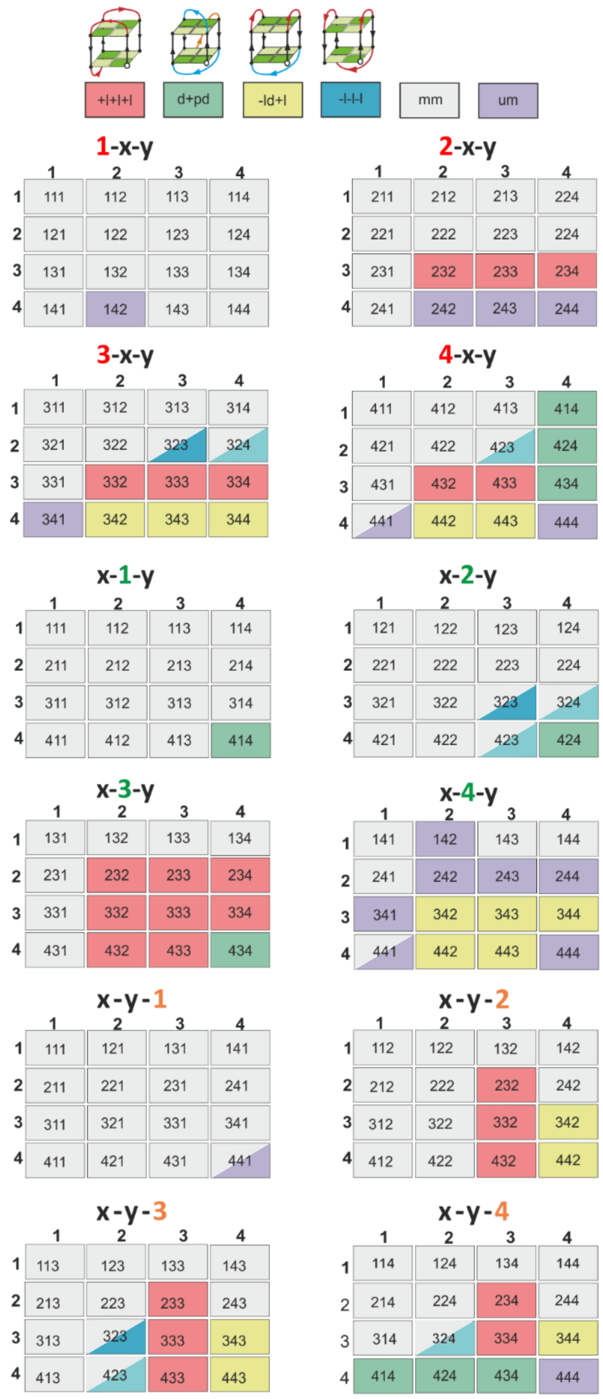

Supplementary Table S1. Classification of the studied molecules based on the imino regions of ^{1}H 1D NMR spectra. Classes A to F correspond to the ones exemplified in the six panels of Figure 4 in the main text.

ID	Group	ID	Group	ID	Group	ID	Group
**111**	E	**211**	F	**311**	F	**411**	F
**112**	E	**212**	A	**312**	A	**412**	A
**113**	F	**213**	D	**313**	D	**413**	B
**114**	D	**214**	D	**314**	D	**414**	B
**121**	E	**221**	D	**321**	A	**421**	B
**122**	F	**222**	F	**322**	A	**422**	C
**123**	A	**223**	D	**323**	B	**423**	C
**124**	D	**224**	D	**324**	B	**424**	A
**131**	F	**231**	D	**331**	D	**431**	D
**132**	D	**232**	A	**332**	A	**432**	B
**133**	D	**233**	A	**333**	A	**433**	B
**134**	D	**234**	A	**334**	A	**434**	A
**141**	D	**241**	D	**341**	B	**441**	D
**142**	F	**242**	B	**342**	A	**442**	A
**143**	C	**243**	C	**343**	B	**443**	B
**144**	D	**244**	B	**344**	B	**444**	C

Supplementary Table S2. List of sequences for which 1D NMR experiments at different DNA and/or KCl concentrations were performed (each applied intervention marked by X). Resulting spectra are provided in appropriate panels of Supplementary Figure 1.

Sequence ID	Applied concentration change[a]			
	0.1x DNA	10x DNA	0.1x K^+	0.1x DNA AND 0.1x K^+
**113**	X		X	X
**123**	X		X	X
**131**	X		X	X
**132**	X		X	
**141**	X		X	
**142**	X		X	
**143**	X		X	
**213**	X		X	X
**222**	X		X	
**223**	X		X	
**224**	X		X	
**231**	X		X	
**241**	X		X	
**322**	X		X	
**323**	X		X	
**324**	X		X	

**331**	X		X	
**341**	X		X	
**414**	X		X	
**421**	X		X	
**422**	X		X	
**423**	X	X	X	
**441**	X		X	

[a]with respect to reference conditions: 0.4 mM DNA and 150 mM KCl

Supplementary Table S3. ^{1}H and ^{31}P chemical shifts of TBA measured at 20 °C. The buffer was 10 mM potassium phosphate, pH 7.6, 150 mM KCl, 0.1 mM EDTA.

	H1'	H2'	H2"	H3'	H4'	H5'	H5"	H6/H8	Me	P	HN
**G1**	6.078	2.981	2.981	5.011	4.415	4.127	4.048	7.442	-	-	12.16
**G2**	6.037	3.032	2.371	5.165	4.438	4.288	-	8.193	-	-4.409	12.18
**T3**	6.211	2.207	2.570	4.900	4.312	3.940	-	7.878	1.994	-2.561	-
**T4**	6.080	2.084	2.656	4.903	4.307	4.217	3.939	7.203	1.073	-	-
**G5**	6.039	3.399	2.905	4.890	4.422	4.306	-	7.467	-	-	12.21
**G6**	5.969	2.793	2.611	5.132	4.463	4.277	4.243	7.715	-	-4.327	12.16
**T7**	6.472	2.510	2.620	4.870	4.443	4.279	4.249	7.908	1.990	-3.364	-
**G8**	5.751	2.010	2.342	4.775	4.011	4.117	4.043	7.483	-	-3.858	-
**T9**	5.844	1.985	2.415	4.643	3.749	3.579	3.034	7.263	1.754	-4.128	-
**G10**	6.064	3.708	2.934	4.927	4.432	4.352	4.155	7.477	-	-4.604	11.95
**G11**	6.028	2.980	2.350	5.152	4.415	4.321	-	8.215	-	-4.580	12.12
**T12**	6.211	2.207	2.570	4.924	4.311	3.939	-	7.885	1.994	-2.518	-
**T13**	6.125	2.116	2.721	4.924	4.310	4.223	3.940	7.263	1.013	-	-
**G14**	6.083	3.536	2.979	4.950	4.445	4.385	4.288	7.507	-	-3.496	12.16
**G15**	6.176	2.709	2.453	4.818	4.269	4.287	4.205	8.084	-	-4.209	12.29

Supplementary References

1. Phan,A.T. (2000) Long-range imino proton-13C J-couplings and the through-bond correlation of imino and non-exchangeable protons in unlabeled DNA. *J. Biomol. NMR*, **16**, 175–178.

2. Dvorkin,S.A., Karsisiotis,A.I. and da Silva,M.W. (2018) Encoding canonical DNA quadruplex structure. *Sci. Adv.*, **4**, eaat3007.
